# Supplementary material for: The role of meteorological factors on mumps incidence among children in Guangzhou, Southern China
Source: PLoS One. 2020 Apr 29;15(4):e0232273. doi: 10.1371/journal.pone.0232273 (PMC7190132; doi:10.1371/journal.pone.0232273)

PLOS ONE

The roles of meteorological factors on mumps incidence among children in Guangzhou, Southern China

--Manuscript Draft--

**Manuscript Number:**	PONE-D-19-31388
**Article Type:**	Research Article
**Full Title:**	The roles of meteorological factors on mumps incidence among children in Guangzhou, Southern China
**Short Title:**	The roles of meteorological factors on mumps incidence among children in Guangzhou
**Corresponding Author:**	Zhoubin Zhang<br>Guangzhou center for disease control and prevention<br>Guangzhou, China, CHINA
**Keywords:**	Distributed Lag Nonlinear Models; Meteorological Factors; Mumps
**Abstract:**	Objective: To explore the association between meteorological factors and the incidence of mumps in Guangzhou, and improve the prevention and intervention measures towards mumps.<br>Methods: Data for daily meteorological factors and mumps cases of children from 2014 to 2018 in Guangzhou were collected from the Guangzhou Meteorological Bureau and the National Notifiable Disease Report System. The distributed lag nonlinear models (DLNMs) were used to evaluate the correlation between meteorological factors and the incidence of mumps in children.<br>Results: The nonlinear lag effects of some meteorological factors were detected. The highest risk was 1.005 (95% CI: 1.001–1.010) at lag day 21, when the daily mean temperature was 25.0ºC. When atmospheric pressure was 1027hPa at lag day 0, the lowest RR was 0.775 (95% CI: 0.655–0.916). For relative humidity, when it was72.0%, the highest RR was 1.014 (95% CI: 1.003–1.026) at lag day 12. For wind velocity, when it was 8.70 m/s, the highest RR was 1.269 (95% CI: 1.033–1.560) at lag day 11. Mean temperature, atmospheric pressure and relative humidity were positively correlated with mumps incidence, which was opposite to the wind speed. Extreme effects of temperature, wind speed, atmospheric pressure and relative humidity on the incidence of mumps in children in Guangzhou were evaluated by a subgroup of gender and age. Conclusion: Our preliminary results offered fundamental information to get a full understanding about epidemic trends of mumps in children in order to develop an early warning system, and strengthen the intervention and prevention of mumps.
**Order of Authors:**	Jianyun Lu
	Zhicong Yang
	Zhoubin Zhang
**Additional Information:**	
**Question**	**Response**
**Financial Disclosure**<br><br>Enter a financial disclosure statement that describes the sources of funding for the work included in this submission. Review the submission guidelines for detailed requirements. View published research articles from *PLOS ONE* for specific examples.<br><br>This statement is required for submission and **will appear in the published article** if	This study was supported by the Medical Health Technology Project for Guangzhou (20181A011051) and the Science and Technology Project of Guangzhou (201804010093) and the Project for Key Medicine Discipline Construction of Guangzhou Municipality (2017-2019-07).

Powered by Editorial Manager® and ProduXion Manager® from Aries Systems Corporation

the submission is accepted. Please make sure it is accurate.<br><br>**Unfunded studies**<br>Enter: *The author(s) received no specific funding for this work.*<br><br>**Funded studies**<br>Enter a statement with the following details:<br>• Initials of the authors who received each award<br>• Grant numbers awarded to each author<br>• The full name of each funder<br>• URL of each funder website<br>• Did the sponsors or funders play any role in the study design, data collection and analysis, decision to publish, or preparation of the manuscript?<br>• **NO** - Include this sentence at the end of your statement: *The funders had no role in study design, data collection and analysis, decision to publish, or preparation of the manuscript.*<br>• **YES** - Specify the role(s) played.<br><br>* typeset	
**Competing Interests**<br><br>Use the instructions below to enter a competing interest statement for this submission. On behalf of all authors, disclose any competing interests that could be perceived to bias this work—acknowledging all financial support and any other relevant financial or non-financial competing interests.<br><br>This statement **will appear in the published article** if the submission is accepted. Please make sure it is accurate. View published research articles from *PLOS ONE* for specific examples.	There is no conflict of interest for the authors.

Powered by Editorial Manager® and ProduXion Manager® from Aries Systems Corporation

**NO authors have competing interests**<br><br>Enter: *The authors have declared that no competing interests exist.*<br><br>**Authors with competing interests**<br><br>Enter competing interest details beginning with this statement:<br><br>*I have read the journal's policy and the authors of this manuscript have the following competing interests: [insert competing interests here]*<br><br>* typeset	
**Ethics Statement**<br><br>Enter an ethics statement for this submission. This statement is required if the study involved:<br><br>• Human participants<br>• Human specimens or tissue<br>• Vertebrate animals or cephalopods<br>• Vertebrate embryos or tissues<br>• Field research<br><br>Write "N/A" if the submission does not require an ethics statement.<br><br>General guidance is provided below. Consult the submission guidelines for detailed instructions. **Make sure that all information entered here is included in the Methods section of the manuscript.**	This work was granted by the Ethics Committee of the Guangzhou Center for Disease Control and Prevention.

**Format for specific study types**<br><br>**Human Subject Research (involving human participants and/or tissue)**<br>• Give the name of the institutional review board or ethics committee that approved the study<br>• Include the approval number and/or a statement indicating approval of this research<br>• Indicate the form of consent obtained (written/oral) or the reason that consent was not obtained (e.g. the data were analyzed anonymously)<br><br>**Animal Research (involving vertebrate animals, embryos or tissues)**<br>• Provide the name of the Institutional Animal Care and Use Committee (IACUC) or other relevant ethics board that reviewed the study protocol, and indicate whether they approved this research or granted a formal waiver of ethical approval<br>• Include an approval number if one was obtained<br>• If the study involved *non-human primates*, add *additional details* about animal welfare and steps taken to ameliorate suffering<br>• If anesthesia, euthanasia, or any kind of animal sacrifice is part of the study, include briefly which substances and/or methods were applied<br><br>**Field Research**<br><br>Include the following details if this study involves the collection of plant, animal, or other materials from a natural setting:<br>• Field permit number<br>• Name of the institution or relevant body that granted permission	
**Data Availability**<br><br>Authors are required to make all data underlying the findings described fully available, without restriction, and from the time of publication. PLOS allows rare exceptions to address legal and ethical concerns. See the PLOS Data Policy and FAQ for detailed information.	Yes - all data are fully available without restriction

A Data Availability Statement describing where the data can be found is required at submission. Your answers to this question constitute the Data Availability Statement and **will be published in the article**, if accepted.<br><br>**Important:** Stating 'data available on request from the author' is not sufficient. If your data are only available upon request, select 'No' for the first question and explain your exceptional situation in the text box.<br><br>Do the authors confirm that all data underlying the findings described in their manuscript are fully available without restriction?	
**Describe where the data may be found in full sentences. If you are copying our sample text, replace any instances of XXX with the appropriate details.**<br><br>• If the data are **held or will be held in a public repository**, include URLs, accession numbers or DOIs. If this information will only be available after acceptance, indicate this by ticking the box below. For example: *All XXX files are available from the XXX database (accession number(s) XXX, XXX.)*.<br>• If the data are all contained **within the manuscript and/or Supporting Information files**, enter the following: *All relevant data are within the manuscript and its Supporting Information files.*<br>• If neither of these applies but you are able to provide **details of access elsewhere**, with or without limitations, please do so. For example:<br><br>*Data cannot be shared publicly because of [XXX]. Data are available from the XXX Institutional Data Access / Ethics Committee (contact via XXX) for researchers who meet the criteria for access to confidential data.*<br><br>*The data underlying the results presented in the study are available from (include the name of the third party*	All relevant data are within the manuscript and its Supporting Information files.

and contact information or URL).<br>• This text is appropriate if the data are owned by a third party and authors do not have permission to share the data.<br><br>* typeset	
Additional data availability information:	

Powered by Editorial Manager® and ProduXion Manager® from Aries Systems Corporation

Manuscript Click here to access/download;Manuscript;manuscript.docx

The roles of meteorological factors on mumps incidence among children in Guangzhou, Southern China

Jian-yun Lu[a#], Zhi-cong Yang [b#], Zhou-bin Zhang[c*]

[a] Department of Infectious Disease Control and Prevention, Guangzhou Center for Disease Control and Prevention, Baiyun District Qi De Road in Guangzhou, Guangdong Province, 510440, China

[b] Director, Guangzhou Center for Disease Control and Prevention, Baiyun District Qi De Road in Guangzhou, Guangdong Province, 510440, China

[c] Vice Director, Guangzhou Center for Disease Control and Prevention, Baiyun District Qi De Road in Guangzhou, Guangdong Province, 510440, China

Their contributions were the same and could be acted as co-first author

* Corresponding author: Guangzhou Center for Disease Control and Prevention, Baiyun District Qi De Road in Guangzhou, Guangdong Province, 510440, China.

Email: zhoubinzhanggz@21cn.com

* Corresponding author at: Guangzhou Center for Disease Control and Prevention, Baiyun District Qi De Road in Guangzhou, Guangdong Province,510440, China.

Email: zhoubinzhanggz@21cn.com

Abstract

Objective: To explore the association between meteorological factors and the incidence of mumps in Guangzhou, and improve the prevention and intervention measures towards mumps.

Methods: Data for daily meteorological factors and mumps cases of children from 2014 to 2018 in Guangzhou were collected from the Guangzhou Meteorological Bureau and the National Notifiable Disease Report System. The distributed lag nonlinear models (DLNMs) were used to evaluate the correlation between meteorological factors and the incidence of mumps in children.

Results: The nonlinear lag effects of some meteorological factors were detected. The highest risk was 1.005 (95% CI: 1.001–1.010) at lag day 21, when the daily mean temperature was 25.0ºC. When atmospheric pressure was 1027hPa at lag day 0, the lowest RR was 0.775 (95% CI: 0.655–0.916). For relative humidity, when it was72.0%, the highest RR was 1.014 (95% CI: 1.003–1.026) at lag day 12. For wind velocity, when it was 8.70 m/s, the highest RR was 1.269 (95% CI: 1.033–1.560) at lag day 11. Mean temperature, atmospheric pressure and relative humidity were positively correlated with mumps incidence, which was opposite to the wind speed. Extreme effects of temperature, wind speed, atmospheric pressure and relative humidity on the incidence of mumps in children in Guangzhou were evaluated by a subgroup of gender and age. **Conclusion:** Our preliminary results offered fundamental information to get a full understanding about epidemic trends of mumps in children in order to develop an early warning system, and strengthen the intervention and prevention of mumps.

Keywords: Distributed Lag Nonlinear Models, Meteorological Factors, Mumps

Abbreviations used:
CI: confidence interval
RR: relative risk
DLNMs: distributed lag nonlinear models

Funding

This study was supported by the Medical Health Technology Project for Guangzhou (20181A011051) and the Science and Technology Project of Guangzhou (201804010093) and the Project for Key Medicine Discipline Construction of Guangzhou Municipality (2017-2019-07).

1. Introduction

Mumps is a common childhood disease focused on the children aged 5-9 years old, which was caused by the mumps virus (1, 2). The clinical feature was inflammatory parotid gland with precursory fever. Most of the patients are mild and self-limiting, but it could cause some severe complications of infections, such as deafness, aseptic meningitis and encephalitis (3). The incubation period of mumps is 12 to 24 days, with a median as 19 (4). Mumps is not only transmitted by droplet spread, but also by direct contact or contaminated fomites (1).

Mumps can be effectively prevented by vaccine, a sharp reduction was observed after the routine vaccination (5), however, many countries, including France (6), Ireland (7) and UK (8) had resurgences of mumps and reported large scale outbreaks over the past decade. Since 2007, the mumps vaccine has been integrated into National Immunization Program in China, and the measles-mumps-rubella (MMR) or measles - mumps (MM) vaccine has been provided for free to children aged 18 -24 months in Guangzhou since 2008 (9). However, the incidence rates of mumps in China rose to 33.9/100,000 and 35.6/ 100,000 during 2011-2012 (10), meanwhile, 10008 and 7856 mumps cases were reported in Guangzhou (9). One dose vaccine program is not available to fully control the epidemic of mumps, even if the immunization coverage rates is about 80% to 90% nationwide (11), the two doses vaccination program was recommended by WHO (12). Hence, mumps is still a serious public health disease for children in Guangzhou, even in China.

The incidence of mumps revealed apparent seasonality in various areas, with the peak in late spring, early summer and winter. Different seasonal distribution was even observed in southern and northern China (10, 11). It hinted that mumps incidence was

influenced by seasonal undulation. Therefore, we indicated that meteorological factors might play a vital role in the spread of mumps. Recently, meteorological effects on some infectious diseases have been extensively explored as early warning signals of possible epidemics, such as hand-foot-mouth disease (13), varicella (14) and influenza (15). Limited studies had been done to explore the association between meteorological factors and the incidence of mumps. A study in Beijing, China showed that rising temperature would cause the increase of mumps incidence (16), whereas a study in Jining exhibited that an increasing trend in the incidence of mumps was observed when the temperature rose above 4 ºC (17). However, a study in Czech Republic showed that the mumps cases had no relationship with drier and warmer weather (18). The discrepancy may be due to different analytical methods, different geographical locations, different climate characteristics and different subpopulations. Although a previous study had shown the correlations between meteorological factors and the overall population incidence of mumps in Guangzhou (19), the effects on the transmission on children are still unclear. Children were the main population of mumps. Thus, it is urgent to explore the relationships on children, the main population of mumps to improve the early warning.

In this study, an ecological methodology was used to analyze the features of mumps in Guangzhou from 2014 to 2018. We built the distributed lag nonlinear models (20), which can flexibly depict the nonlinear correlation and describe delayed effects in time-series study, to analyze the effects of meteorological factors on mumps transmission in children. The findings of our study may be helpful to develop an early warning system for mumps epidemic in children, improve the preventive measurement and reduce the risk of mumps infection for children.

2. Materials and methods

2.1. Study region

Guangzhou, is the third largest city in China with a total of 8.97 million registered inhabitants. It has a typical meteorological feature of humid subtropical, which locates at 112°57'E to 114°3'E and at 22°26'N to 23°56'N. In Guangzhou, the summer season is wet and hot, whereas the winter season is dry and cool.

2.2. Data sources

In this research, the clinical diagnosis cases of mumps under 18 years old were obtained from the National Notifiable Disease Report System from Jan, 2014 to Dec, 2018. The clinical feature of mumps is defined as an acute onset of bilateral or unilateral tender, with self-limited swelling of the parotid or other salivary glands sustaining more than 2 days. Mumps was the notifiable disease in Guangzhou, consequently, compulsory notification is required to report to local health authorities with 24 hours through National Notifiable Disease Surveillance System for all clinicians.

The daily meteorological data, including mean temperature (°C), diurnal temperature range (°C) (it was equal to the highest temperature minus the lowest temperature), atmospheric pressure (hPa), aggregate rainfall (mm), wind speed (m/s), relative humidity (%), and sunshine hours (hours) were collected simultaneously from the Guangzhou Meteorological Bureau.

2.3. Statistic analysis

We used descriptive analysis for meteorological variables and the occurrence of mumps in Guangzhou. According to the scatter plots, we got a conclusion that the correlation between the meteorological factors and mumps were nonlinear. For this reason, the Distributed Lag Nonlinear Models (DLNMs) were applied to detect the relationships between meteorological variables and the occurrence of mumps. Atmospheric pressure had a strongly correlation with average temperature (r=-0.82,

P<0.01) by the result of Spearman's correlation, hence the two variables was not put together into a model to avoid the potential collinearity problem. A Poisson regression, constructed with the quasi-Poisson function, was established to analyze the over-dispersion in the daily cases of mumps in the DLNMs. The calculation is shown as below:

$$Log[E(Y_t)] = \alpha + \sum NS_i(X_i, df_i) + \delta Dow + \delta Holiday_t + NS(Time, 7 \text{ per year})$$

Where Y_t stands for the daily cases of mumps in day t; both α and δ are the intercept; NS represents natural cubic spline; X stands for meteorological variables, such as temperature, diurnal temperature range, rainfall, atmospheric pressure, wind speed, relative humidity and sunshine hours; df means the degree of freedom; Holiday is defined as a binary variable, if day t is public holiday or school vacation, it is marked as 1, otherwise it equals 0; Dow represents the day of week effect; and Time denotes long term trends and seasonality. We select the df of day to control long term trends, seasonality and meteorological variables by the Akaike information criterion (AIC). Based on the AICs, we choose df=3 for NS to the meteorological variables mentioned above. After we combined with the incubation period of mumps, the df=24 was chosen to mean temperature, atmospheric pressure, and wind speed, the df=14 was chosen to relative humidity.

The effect when the value of meteorological variables beyond the 97.5 percentile was defined as an extreme high effect, while the value below the 2.5 percentile, it was defined as an extreme low effect.

All statistical tests were two-sided, if the ~~*P-value*~~ <0.05, it had statistical significance. R software (version 3.5.3) was applied to all analyses, and the DLNMs were built by package named dlnm.

We conducted the sensitivity analyses by shifting the df (5–9) per year for time to

control for seasonality and long-term trends, and changing the df (4–7) for mean temperature, atmospheric pressure, diurnal temperature range, wind speed, accumulated rainfall, relative humidity, and sunshine hours.

2.4. Ethical permission

This work was granted by the Ethics Committee of the Guangzhou Center for Disease Control and Prevention.

3. Results

From Jan1st, 2014 to Dec 31st, 2018, a total amount of 9842 mumps cases under 18 years old were reported in Guangzhou. During 2014 to 2018, the description of mumps cases was 1962, 2077, 1838, 1977, and 1988, respectively. The ratio of gender was 1.83 (6362 males to 3480 females). Among the groups aged 0-3 years, 4-6 years, 7-14 years and 15-17 years, the cases were 2197 (22.32%), 3773 (38.34%), 3672 (37.31%), and 200 (2.03%), respectively. The peak of mumps in the study period occurred from May to July, with a total of 3087 cases (31.37%). **(Figure 1)**.

Fig 1. The distribution of daily mumps cases among children and meteorological variables in Guangzhou, 2014–2018.

The description of meteorological variables and mumps cases were shown in Table 1. The daily mean temperature was 22.24℃, and the mean of diurnal temperature range, aggregate rainfall, atmospheric pressure, wind speed, relative humidity and hours of sunshine were 8.20℃, 6.04mm, 1005.41hPa, 2.16m/s, 79.19%, and 4.42 hours, respectively. **(Table 1).**

Table 1

Daily meteorological variables and mumps cases among children in Guangzhou, 2014–2018.

Variables	Mean	Std. deviation	Percentile			Min.	Max.
			25	50	75		
Cases	5.00	3.00	3.00	5.00	7.00	0.00	16.00
Mean temperature	22.24	6.15	17.65	23.60	27.45	3.49	31.80
Diurnal temperature range	8.20	3.47	6.90	9.30	11.60	0.90	24.40
Aggregate rainfall	5.56	14.15	0.00	0.00	3.90	0.00	222.00
Atmospheric pressure	1005.41	7.36	1000.05	1005.20	1010.52	954.72	1027.84
Wind speed	2.16	0.90	1.50	1.90	2.58	0.83	8.70
Relative humidity	79.19	10.73	73.89	80.27	87.00	27.75	99.79
Sunshine hours	4.42	3.79	0.30	4.00	8.18	0.00	12.00

Analysis of Spearman correlations (**Table 2)** revealed that the incidence of mumps was positively correlated with mean temperature, relative humidity, aggregate rainfall, and hours of sunshine. To the contrary, wind speed, atmospheric pressure showed a negative correlation with the incidence of mumps. ~~The **Table 2** meanwhile, provided the matrix of Spearman's correlation coefficients with the meteorological factors.~~ Mean temperature had a strong correlation to ~~Atmospheric pressure~~ (*r*=-0.82, *P*<0.01).

Table 2

Spearman's correlation coefficient matrix for mumps cases and meteorological variables in Guangzhou, 2014–2018.

Variables	Cases	Temperature	DTR	Rainfall	Air pressure	Wind speed	Relative humidity	Sunshine hours
Cases	1.00							
Temperature	0.33**	1.00						
DTR	-0.02	0.10**	1.00					
Rainfall	0.09**	0.12**	-0.41**	1.00				
Air Pressure	-0.29**	-0.82**	0.13**	-0.28**	1.00			
Wind speed	-0.08**	-0.22**	-0.21**	-0.07**	0.10**	1.00		
Relative humidity	0.10**	0.15**	-0.53**	0.64**	-0.37**	-0.30**	1.00	
Sunshine hours	0.07**	0.35**	0.72**	-0.46**	-0.10**	0.01	-0.58**	1.00

$^{**}P<0.01$

The **Figure 2** illustrated the correlation between mumps cases and meteorological variables with different lag days in Guangzhou. Specific feature was observed in various variables with a nonlinear curve. The highest risk was 1.005 (95% CI: 1.001–1.010) at lag day 21, when the daily mean temperature was 25.0ºC. When atmospheric pressure was 1027hPa at lag day 0, the lowest RR was 0.775 (95% CI: 0.655–0.916). For relative humidity, when it was72.0%, the highest RR was 1.014 (95% CI: 1.003–1.026) at lag day 12. For wind velocity, when it was 8.70 m/s, the highest RR was 1.269 (95% CI: 1.033–1.560) at lag day 11.

Fig 2. Plots of the relative risk of climatic factors on the incidence of mumps in children, including mean temperature, diurnal temperature range, atmospheric pressure, wind speed, relative humidity and sunshine hours.

For a better explanation, the one-dimensional curves were plotted about the overall effects of meteorological factors over the corresponding lag days (**Figure 3**). In a word, mean temperature, atmospheric pressure, and relative humidity was associated with mumps cases, which was on the contrary to the wind speed. The relative risk (RR) of mean temperature significantly increased from 10 ºC to 23.6 ºC, whereas the RR of wind speed significantly declined from 1.9 m/s to 4.5 m/s. Atmospheric pressure and relative humidity both showed a reverse U-shape curve relationship with mumps. However, the RRs just had a significant rising trend before 992 pHa and 60%, respectively. There was no significant relationship between other meteorological variables in our study and the incidence of mumps.

Fig 3. The estimated overall effects of mean temperature, diurnal temperature range, atmospheric pressure, wind speed, relative humidity, and sunshine hours along corresponding lag days. The Y lab represents the value of relative risk, the X lab

represents the value of relevant variables. The red lines represent mean relative risks and the grey regions represent 95% CIs. The black vertical line represents the medians of the climatic factors, and the dotted lines represent the 2.5 percentile and the 97.5 percentile for the meteorological factors, respectively.

The cumulative extreme effects of meteorological factors on mumps along the corresponding lag days were drawn in **Figure 4**. The **Table 3** showed that the extreme low effect of temperature was significant and the lowest RR was 0.88 (95% CI: 0.83–0.93) at lag day 0, whereas the extreme high effect of temperature was not significant. On the contrary, the extreme high effects of atmospheric pressure and wind speed were statistically significant, but the extreme low effects were not. For atmospheric pressure, the lowest RR was 0.88 (95% CI: 0.82–0.94) at lag day 0. For wind speed, the lowest RR was 0.94 (95% CI: 0.89–0.98) at lag day 0. Regarding relative humidity, the RR of extremely high effect was 0.96 (95% CI: 0.94–0.99) at lag day 2, and the RR of extremely low effect was 0.96 (95% CI: 0.93–0.99) at lag day 2. For the extremely high and low effect of sunshine hours, the RR was 1.03 (95% CI: 1.02–1.04) at lag day 2 and 1.02 (95% CI: 1.01–1.03) at lag day 17, respectively.

Fig 4. The extreme effects of mean temperature, diurnal temperature range, atmospheric pressure, wind speed, relative humidity, and sunshine hours with extreme high effects (97.5%) and extreme low effects (2.5%). The Y lab represents the value of relative risk, the X lab represents the value of lag day. The red lines represent mean relative risks and grey regions represent 95% CIs.

Table 3

The extreme effects of meteorological variables on the incidence of mumps among children along the lag days in Guangzhou, 2014–2018.

Meteorological variables	Lag day	High effect (97.5%)		Lag day	Low effect (2.5%)	
		RR	95% CI		RR	95% CI
Mean temperature	/	/	/	0	0.88	0.83–0.93
Atmospheric pressure	0	088	0.82-0.94	/	/	/
Wind speed	0	0.94	0.89-0.98	/	/	/
Relatively humidity	2	0.96	0.94-0.99	2	0.96	0.93–0.99

In the analyses by subpopulation, the extreme low effects of temperature, atmospheric pressure, and relative humidity, and the extreme high effect of wind speed showed statistical significance in the female population, but no significant extreme effect was observed in the male population. The extreme low effect of temperature was observed between 4-6 years group (RR=0.25, 95%CI: 0.11-0.60) and 15-17 years group (RR=0.01, 95%CI: 0.00-0.57). The extreme high effect of atmospheric pressure was revealed at the 4-6years group (RR=0.46, 95%CI: 0.21-0.98). (**Table 4**)

Sensitivity analyses indicated alike results after the changes in the df for long-term trends and seasonality. Altering the df for temperature, atmospheric pressure, wind speed and relative humidity, the original models revealed a better robustness than

alternative ones.

Table 4

The cumulative extreme effects of meteorological variables on mumps cases of children by sex and age.

Variables	Mean temperature				Wind speed				Relative humidity				Atmospheric pressure			
	low effect		high effect		low effect		high effect		low effect		high effect		low effect		high effect	
	RR	95%CI	RR	95%CI	RR	95%CI	RR	95%CI	RR	95%CI	RR	95%CI	RR	95%CI	RR	95%CI
total	**0.56**	**0.32-0.99**	0.99	0.70-1.40	0.77	0.47-1.28	0.64	0.40-1.01	**0.76**	**0.61-0.94**	0.89	0.67-1.17	**0.70**	**0.49-0.99**	0.75	0.45-1.24
male	0.88	0.44-1.73	0.91	0.59-1.39	0.76	0.41-1.41	0.88	0.50-1.56	0.95	0.73-1.23	1.06	0.75-1.49	0.87	0.56-1.34	0.81	0.43-1.50
female	**0.23**	**0.10-0.62**	1.12	0.66-2.02	0.77	0.74-1.71	**0.34**	**0.16-0.72**	**0.50**	**0.35-0.72**	0.64	0.41-1.00	**0.47**	**0.26-0.83**	0.64	0.28-1.45
< 4years	1.83	0.60-5.65	1.40	0.69-2.83	0.99	0.36-2.71	0.74	0.29-1.87	0.73	0.48-1.11	0.70	0.39-1.24	0.54	0.26-1.09	0.97	0.37-2.55
4-6 years	**0.25**	**0.11-0.60**	1.01	0.58-1.75	0.68	0.31-1.49	0.51	0.25-1.05	0.77	0.56-1.07	1.28	0.83-1.96	0.81	0.46-1.42	**0.46**	**0.21-0.98**
7-13 years	0.82	0.32-2.10	0.83	0.48-1.44	0.79	0.35-1.80	0.81	0.37-1.76	0.76	0.52-1.11	0.72	0.45-1.14	0.73	0.41-1.30	1.25	0.51-3.05
14-17 years	**0.01**	**0.00-0.57**	0.18	0.01-2.74	0.35	0.01-16.28	0.05	0.00-2.18	0.48	0.07-3.29	0.38	0.05-3.09	0.20	0.01-2.66	0.08	0.00-3.08

4. Discussion

In the study, the mumps cases of children in Guangzhou fluctuated seasonally with the epidemic peak from May to July. The trough of mumps cases in children occurred in February, coinciding with the winter holiday in school. However, our result was different from the study in Ireland, which dedicated a relatively low mumps cases in summer and peaked in autumn (7), and a study in the Netherlands with two seasonality peaks in spring and autumn (21). It indicated that the effect of meteorological factors on the occurrence of mumps may be distinct in various regions with particularly climatic features.

Climatic variables such as temperature, relative humidity and wind speed have been proven to play a key role in the occurrence of certain infectious diseases (22-24). Recently, it drew more and more attention to the effects of meteorological factors on mumps incidence. DLNMs were widely applied to evaluate these nonlinear correlations. For example, the previous DLNMs studies reported nonlinear and lag effects of meteorological factors on the overall incidence of mumps (25).

As far as we know, it is the first research to apply DLNMs to detect the effects of meteorological variables on the mumps cases of children in a subtropical city in China. The nonlinear relationships were observed in some meteorological variables. When the mean temperature ranged from 10 ºC to 23.6 ºC, the RR showed an increased trend. Our finding was not fully consistent with previous studies. The study in Taiwan (26) showed that the mumps cases began to increase at the temperature of 20 ºC, and started to decline after 25 ºC, whereas a study in Jining, China (17) and a study focused on children in Japan (27) both reported linear positive association between mumps cases and temperature. A previous DLNM study in Guangzhou (19) also revealed that mean

temperature positively correlated to the RR of the overall population incidence of mumps. The discrepancy may be due to the different methodologies, geographic location and climatic characteristics. The incidence of mumps depended on the contact rate, which might be influenced by social behavior of children. A previous epidemiology study showed that adolescents usually join more physical activities in the warmer months than in the colder months (28). Children could contact with each other in warmer weather, which might result in the increase of mumps cases. When the temperature became much higher, the children might decrease the outdoor activities and reduce the risk of mumps infection. These might be the explanation for our finding in children in Guangzhou.

The relative humidity was dedicated to have a negative impact on mumps cases with a threshold of 54% in Jining, China (17), but a study in Taiwan (26) pointed out that no correlation was found between relative humidity and mumps cases. Our finding in children, which was inconsistent with previous studies mentioned before, showed a U-shape curve between relative humidity and mumps in Guangzhou, although the RR just had a statistically significant increase before the 60% of relative humidity. Meanwhile the nonlinear extreme high or low effects of relative humidity on mumps cases were both found in children. Seldom research was available to the mechanism about the effect of relative humidity on mumps, it should be urged to carry out in the future. One possible reason might be as follows. Droplet spread is one of the transmission way of mumps virus (1). When the relative humidity was low, the water in the droplets evaporated quickly. The longer the droplet with mumps viruses remains airborne, the easier the mumps infect the children (29). As the relative humidity increases, the effects became insignificant, however an extreme high effect of relative humidity was observed with the RR as 0.96 (95%CI: 0.93-0.99) at lag day 2, it may be

explained that high relative humidity often coincides with heavy rain, which will make children more time to stay at home (30) and reduce the contact rate. More studies should be carried out to look for the biological evidence.

Wind could affect the suspension time of viruses and their diffuse distance (31, 32). Our finding revealed that the RR decreased as the wind speed passed 1.9 m/s till 4.9m/s. Moreover, the high effect of wind speed was in our study, which showed a RR as 0.94 (95%CI: 0.89-0.98). Similarly, the study in Fujian province (25) showed a negative relationship between wind speed and mumps cases. In view of the resuspension phenomenon, the concentration of local virus may be diluted by higher wind velocity (31, 33), which could give a possible explanation for our finding.

In our study, the extreme low effect of mean temperature, atmospheric pressure, and relative humidity, and the extreme high effect of wind speed were found in females with the RRs under 1. However, no extreme effect was detected in males. The reason may be due to the factors such as physiological gender differences, and the discrepancy in behavior patterns (34). For example, in the extreme weather condition, the males were more active will be more likely to play with friends, which will increase the contact rate with mumps patient.

Our results showed a positive correlation before 1000.05hPa, after which point, the correlation was not statistically significant. However, the previous studies showed different results. For instance, a study in 10 cities in Guangxi province in China (35) dedicated that no statistical relationship was found between atmospheric pressure and the incidence of mumps, whereas a study in Beijing (16) showed that atmospheric pressure had a negative association with mumps cases. Unfortunately, limited study was available for the underlying mechanism. An explanation may be as below. As the atmospheric pressure increased in the beginning, the cold air would enter and promote

the spread of the virus, which leading to a high virus abundance in the atmosphere. After exceeding certain value of atmospheric pressure, the effect mentioned above was offset by high wind speed. However, further studies should be done to explore the foundational mechanism in future.

Several limitations of our study should be acknowledged. At first, the data were only obtained in Guangzhou, southern China, hence it cannot stand for other cities or countries. Secondly, we used the meteorological data instead of the individual exposure condition, so it may lead to bias our results. What's more, it was incapable for us to remove the possibility that some potential confounding factors existed in our research, including host susceptibility, preventive measures, geographic factors, and population density. Finally, the data of human serum antibody of mumps and the mumps vaccination information was not available in our study. Therefore, more studies should be undertaken to take these variables into consideration.

5. Conclusions

In general, we revealed the significant nonlinear association between certain climatic variables and mumps cases in children in Guangzhou. At a certain corresponding range, mean temperature, atmospheric pressure, and relative humidity was positively correlated with the incidence of mumps in children, which was opposite to the wind speed. Extreme effects of variable mentioned above in mumps cases were detected by a subgroup of gender and age. These findings provide the underlying information to get a better understanding of the epidemical tendency of mumps. It will help to improve the early warning system, reinforce the prevention and intervention for risk factors of mumps.

Acknowledgments

We thanked the Guangzhou Meteorological Bureau for offering climatic data for our work.

Reference

1. Hviid A, Rubin S, Muhlemann K. Mumps. Lancet (London, England). 2008;371(9616):932-44.https://doi.org/10.1016/s0140-6736(08)60419-5.

2. Rubin S, Eckhaus M, Rennick LJ, Bamford CG, Duprex WP. Molecular biology, pathogenesis and pathology of mumps virus. The Journal of pathology. 2015;235(2):242-52.https://doi.org/10.1002/path.4445.

3. Grennan D. Mumps. Jama. 2019;322(10):1022.https://doi.org/10.1001/jama.2019.10982.

4. Richardson M, Elliman D, Maguire H, Simpson J, Nicoll A. Evidence base of incubation periods, periods of infectiousness and exclusion policies for the control of communicable diseases in schools and preschools. The Pediatric infectious disease journal. 2001;20(4):380-91.https://doi.org/10.1097/00006454-200104000-00004.

5. Galazka AM, Robertson SE, Kraigher A. Mumps and mumps vaccine: a global review. Bulletin of the World Health Organization. 1999;77(1):3-14

6. Vygen S, Fischer A, Meurice L, Mounchetrou Njoya I, Gregoris M, Ndiaye B, et al. Waning immunity against mumps in vaccinated young adults, France 2013. Euro Surveill. 2016;21(10):30156.https://doi.org/10.2807/1560-7917.es.2016.21.10.30156.

7. Whyte D, O'Dea F, McDonnell C, O'Connell NH, Callinan S, Brosnan E, et al. Mumps epidemiology in the mid-west of Ireland 2004-2008: increasing disease burden in the university/college setting. Euro Surveill. 2009;14(16)

8. Aasheim ET, Inns T, Trindall A, Emmett L, Brown KE, Williams CJ, et al. Outbreak of mumps in a school setting, United Kingdom, 2013. Hum Vaccin Immunother. 2014;10(8):2446-9.https://doi.org/10.4161/hv.29484.

9. Fu C, Xu J, Cai Y, He Q, Zhang C, Chen J, et al. Effectiveness of one dose of mumps vaccine against clinically diagnosed mumps in Guangzhou, China, 2006-2012. Hum Vaccin Immunother. 2013;9(12):2524-8.https://doi.org/10.4161/hv.26113.

10. Su QR, Liu J, Ma C, Fan CX, Wen N, Luo HM, et al. Epidemic profile of mumps in China during 2004-2013. Zhonghua yu fang yi xue za zhi [Chinese journal of preventive medicine]. 2016;50(7):611-4.https://doi.org/10.3760/cma.j.issn.0253-9624.2016.07.009.

11. Cui A, Zhu Z, Hu Y, Deng X, Sun Z, Zhang Y, et al. Mumps Epidemiology and Mumps Virus Genotypes Circulating in Mainland China during 2013-2015. PLoS One. 2017;12(1):e0169561.https://doi.org/10.1371/journal.pone.0169561.

12. WHO. Mumps virus vaccines: WHO position paper. Wkly Epidemiol Rec. 2017;82:51-60
13. Yu G, Li Y, Cai J, Yu D, Tang J, Zhai W, et al. Short-term effects of meteorological factors and air pollution on childhood hand-foot-mouth disease in Guilin, China. Sci Total Environ. 2019;646:460-70.https://doi.org/10.1016/j.scitotenv.2018.07.329.
14. Yang Y, Geng X, Liu X, Wang W, Zhang J. Association between the incidence of varicella and meteorological conditions in Jinan, Eastern China, 2012-2014. BMC Infect Dis. 2016;16:179.https://doi.org/10.1186/s12879-016-1507-1.
15. Pan M, Yang HP, Jian J, Kuang Y, Xu JN, Li TS, et al. Association of meteorological factors with seasonal activity of influenza A subtypes and B lineages in subtropical western China. Epidemiol Infect. 2019;147:e72.https://doi.org/10.1017/s0950268818003485.
16. Hao Y, Wang RR, Han L, Wang H, Zhang X, Tang QL, et al. Time series analysis of mumps and meteorological factors in Beijing, China. BMC Infect Dis. 2019;19(1):435.https://doi.org/10.1186/s12879-019-4011-6.
17. Li R, Lin H, Liang Y, Zhang T, Luo C, Jiang Z, et al. The short-term association between meteorological factors and mumps in Jining, China. Sci Total Environ. 2016;568:1069-75.https://doi.org/10.1016/j.scitotenv.2016.06.158.
18. Hubalek Z. North Atlantic weather oscillation and human infectious diseases in the Czech Republic, 1951-2003. European Journal of Epidemiology. 2005;20(3):263-70.https://doi.org/10.1007/s10654-004-6518-3.
19. Yang Q, Yang Z, Ding H, Zhang X, Dong Z, Hu W, et al. The relationship between meteorological factors and mumps incidence in Guangzhou, China, 2005-2012. Hum Vaccin Immunother. 2014;10(8):2421-32.https://doi.org/10.4161/hv.29286.
20. Gasparrini A. Distributed Lag Linear and Non-Linear Models in R: The Package dlnm. J Stat Softw. 2011;43(8):1-20
21. Sane J, Gouma S, Koopmans M, de Melker H, Swaan C, van Binnendijk R, et al. Epidemic of mumps among vaccinated persons, The Netherlands, 2009-2012. Emerg Infect Dis. 2014;20(4):643-8.https://doi.org/10.3201/eid2004.131681.
22. Kaffenberger BH, Shetlar D, Norton SA, Rosenbach M. The effect of climate change on skin disease in North America. Journal of the American Academy of Dermatology. 2017;76(1):140-7.https://doi.org/10.1016/j.jaad.2016.08.014.
23. Cheng Q, Bai L, Zhang Y, Zhang H, Wang S, Xie M, et al. Ambient temperature, humidity and hand, foot, and mouth disease: A systematic review and meta-analysis. Sci Total Environ. 2018;625:828-36.https://doi.org/10.1016/j.scitotenv.2018.01.006.
24. Peci A, Winter AL, Li Y, Gnaneshan S, Liu J, Mubareka S, et al. Effects of Absolute Humidity, Relative Humidity, Temperature, and Wind Speed on Influenza Activity in Toronto, Ontario, Canada. Applied and environmental microbiology. 2019;85(6).https://doi.org/10.1128/aem.02426-18.
25. Hu W, Li Y, Han W, Xue L, Zhang W, Ma W, et al. Meteorological factors and the

incidence of mumps in Fujian Province, China, 2005-2013: Non-linear effects. Sci Total Environ. 2018;619-620:1286-98.https://doi.org/10.1016/j.scitotenv.2017.11.108.

26. Ho YC, Su BH, Su HJ, Chang HL, Lin CY, Chen H, et al. The association between the incidence of mumps and meteorological parameters in Taiwan. Hum Vaccin Immunother. 2015;11(6):1406-12.https://doi.org/10.1080/21645515.2015.1029687.

27. Onozuka D, Hashizume M. Effect of weather variability on the incidence of mumps in children: a time-series analysis. Epidemiol Infect. 2011;139(11):1692-700.https://doi.org/10.1017/s0950268810002967.

28. Belanger M, Gray-Donald K, O'Loughlin J, Paradis G, Hanley J. Influence of weather conditions and season on physical activity in adolescents. Ann Epidemiol. 2009;19(3):180-6.https://doi.org/10.1016/j.annepidem.2008.12.008.

29. Tang JW. The effect of environmental parameters on the survival of airborne infectious agents. J R Soc Interface. 2009;6 Suppl 6:S737-46.https://doi.org/10.1098/rsif.2009.0227.focus.

30. Guo Q, Dong Z, Zeng W, Ma W, Zhao D, Sun X, et al. The effects of meteorological factors on influenza among children in Guangzhou, China. Influenza and other respiratory viruses. 2019;13(2):166-75.https://doi.org/10.1111/irv.12617.

31. Zhong X, Qi, J., Li, H., Dong, L., & Gao, D. Seasonal distribution of microbial activity in bioaerosols in the outdoor environment of the Qingdao coastal region. Atmospheric Environment. 2016;140(06):506-13.https://doi.org/doi.org/10.1016/j.atmosenv.2016.06.034.

32. Jones AM, Harrison RM. The effects of meteorological factors on atmospheric bioaerosol concentrations--a review. Sci Total Environ. 2004;326(1-3):151-80.https://doi.org/10.1016/j.scitotenv.2003.11.021.

33. Sabariego S, Diaz de la Guardia C, Alba F. The effect of meteorological factors on the daily variation of airborne fungal spores in Granada (southern Spain). Int J Biometeorol. 2000;44(1):1-5

34. Yu L, Winter S, Xie D. The child play behavior and activity questionnaire: a parent-report measure of childhood gender-related behavior in China. Archives of sexual behavior. 2010;39(3):807-15.https://doi.org/10.1007/s10508-008-9403-4.

35. Yu G, Yang R, Yu D, Cai J, Tang J, Zhai W, et al. Impact of meteorological factors on mumps and potential effect modifiers: An analysis of 10 cities in Guangxi, Southern China. Environmental research. 2018;166:577-87.https://doi.org/10.1016/j.envres.2018.06.047.

Fig 1

Click here to access/download;Figure;Fig 1.tiff

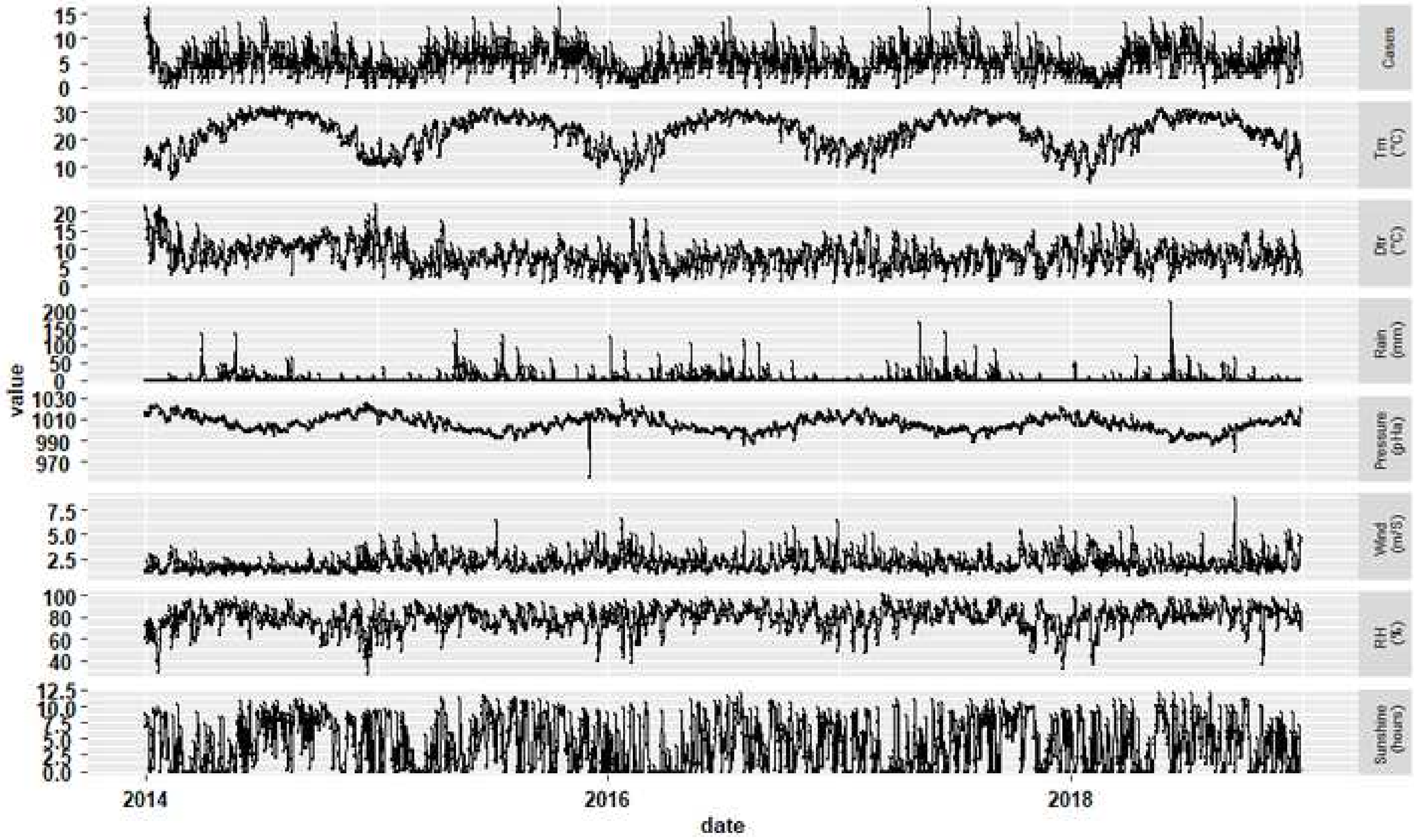

Fig 2

Click here to access/download;Figure;Fig 2.tiff

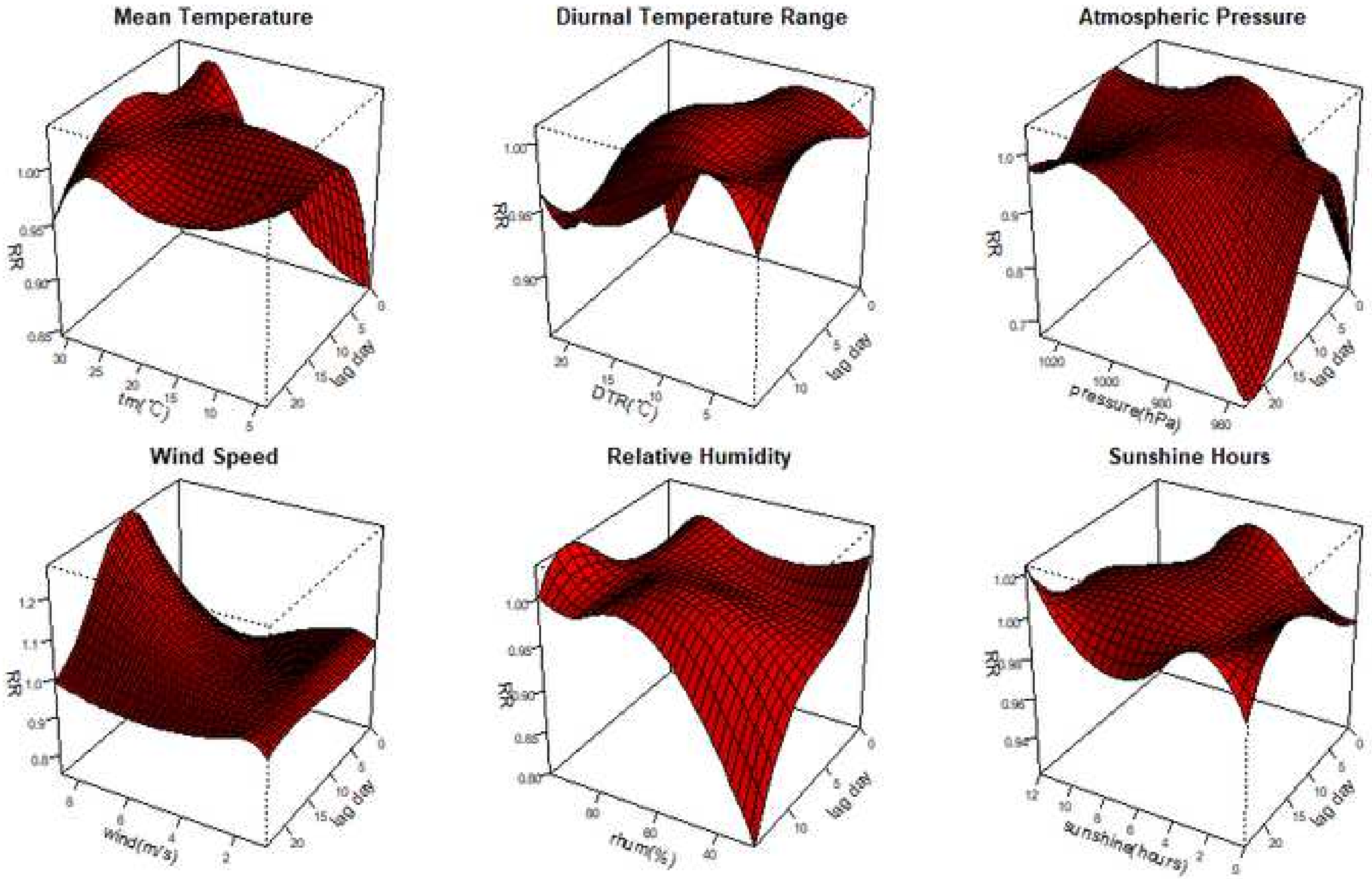

Fig 3

Click here to access/download;Figure;Fig 3.tiff

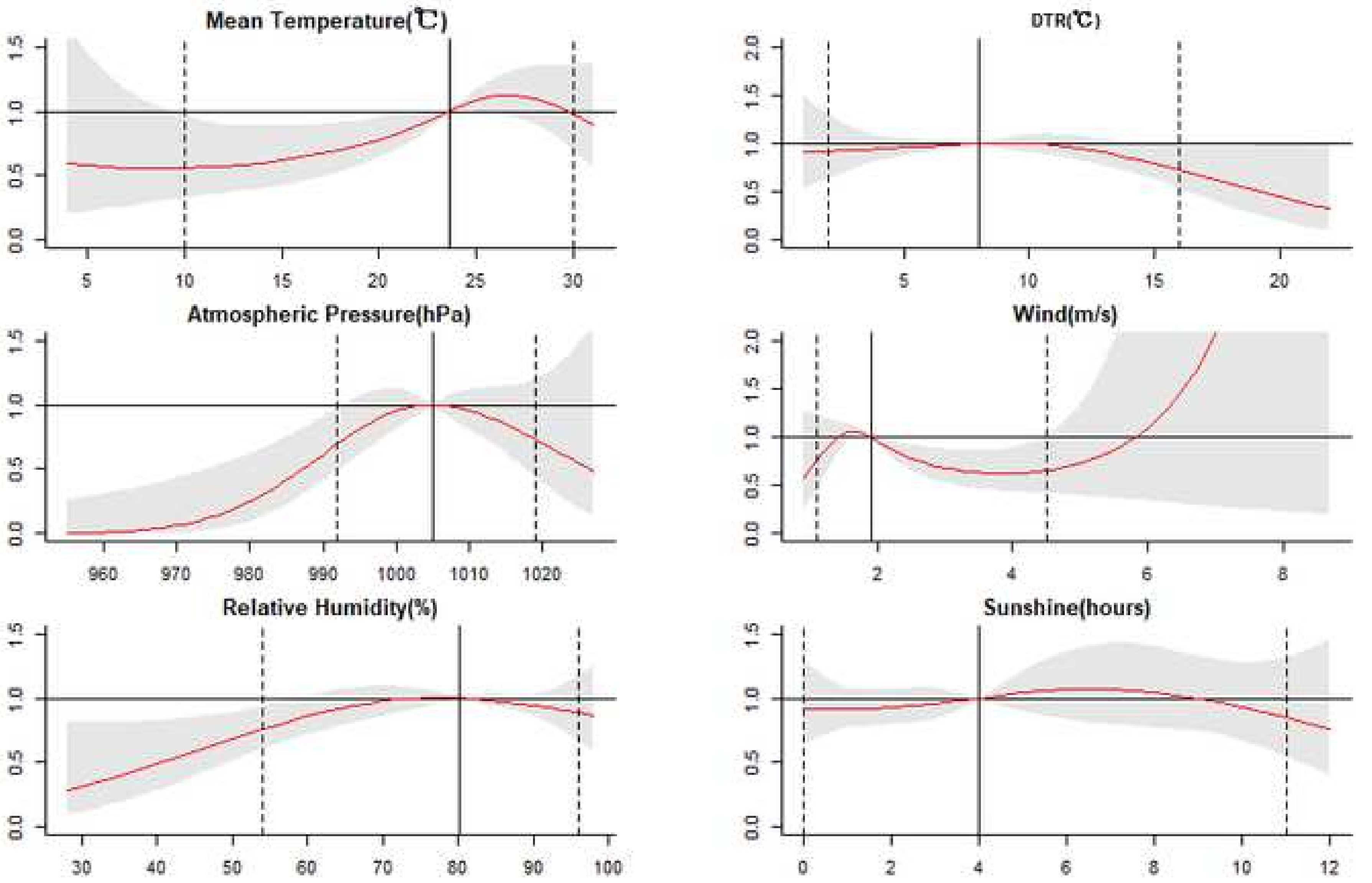

Fig 4

Click here to access/download;Figure;Fig 4.tif

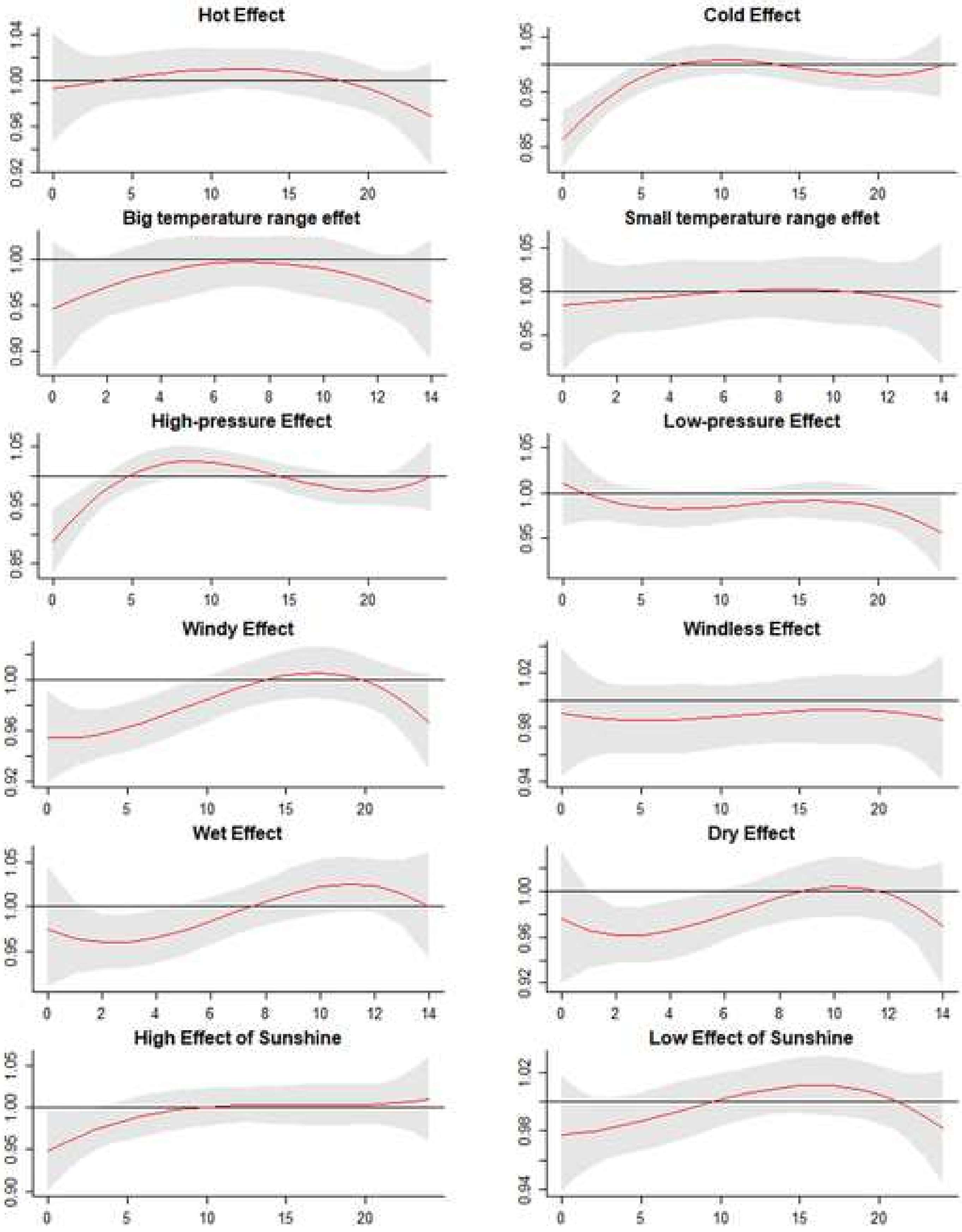

Supplement: Attachment — Submitted filename: PONE-D-19-31388_R1.pdf [file pone.0232273.s002.pdf]
