# Supplementary material for: The role of meteorological factors on mumps incidence among children in Guangzhou, Southern China
Source: PLoS One. 2020 Apr 29;15(4):e0232273. doi: 10.1371/journal.pone.0232273 (PMC7190132; doi:10.1371/journal.pone.0232273)
Supplement: Attachment — Submitted filename: PONE-D-19-31388_R1_RV.pdf [file pone.0232273.s004.pdf]

PLOS ONE

The roles of meteorological factors on mumps incidence among children in Guangzhou, Southern China

--Manuscript Draft--

**Manuscript Number:**	PONE-D-19-31388R1
**Article Type:**	Research Article
**Full Title:**	The roles of meteorological factors on mumps incidence among children in Guangzhou, Southern China
**Short Title:**	The roles of meteorological factors on mumps incidence among children in Guangzhou
**Corresponding Author:**	Zhoubin Zhang<br>Guangzhou center for disease control and prevention<br>Guangzhou, China, CHINA
**Keywords:**	distributed lag nonlinear models; climatic factors; effect
**Abstract:**	Mumps, a common childhood disease, has a high incidence in Guangzhou city, China. It has been proven that mumps is influenced by seasonality. However, the role of meteorological factors among children is yet to be fully ascertained. This study explored the association between meteorological factors and the incidence of mumps among children in Guangzhou. Distributed lag nonlinear models were used to evaluate the correlation between meteorological factors and the incidence of mumps among children from 2014–2018. The nonlinear lag effects of some meteorological factors were detected. Mean temperature, atmospheric pressure, and relative humidity were positively correlated with mumps incidence, contrary to that of wind speed. Extreme effects of temperature, wind speed, atmospheric pressure, and relative humidity on the incidence of mumps among children in Guangzhou were evaluated in a subgroup analysis according to gender and age. Our preliminary results offered fundamental information to better understand the epidemic trends of mumps among children to develop an early warning system, and strengthen the intervention and prevention of mumps.
**Order of Authors:**	Jianyun Lu
	Zhicong Yang
	Mengmeng Ma
	Zhoubin Zhang
**Response to Reviewers:**	Dear Editor,<br>We acknowledge with thanks the receipt of the comments of the reviewer. These comments/requirements were highly beneficial in the modification of the manuscript. The manuscript was revised according to all these comments. All modifications and/or corrections are highlighted by using the track changes mode. We would like to thank Editage (www.editage.cn) for English language editing. A point-to-point response to the reviewers' comments was attached. We appreciate you kindly offer the opportunity to transfer our manuscript.<br>Additional Editor Comments<br>Question No1: Abstract should be written according to Plos One models;<br>Response to the question No.1: We re-wrote the Abstract according to Plos One models.<br>Question No.2：It is necessary to include at the beginning of the Abstract and at the end of the Introduction what are the hypotheses of this research. This can be done in the form of a question;<br>Response to the question No.2: We added the hypotheses of this research in the article ,L81-86"However, the nonlinear and lag effects of climatic factors on the transmission among children, who are the main population affected by mumps, are still unclear at present. A study conducted in Japan explored the linear relationship between meteorological factors and mumps incidence among children via negative binomial regression analysis (24). What are the roles of meteorological factors on mumps incidence among children? There is an urgent need to explore these nonlinear relationships using lag effects.."

Powered by Editorial Manager® and ProduXion Manager® from Aries Systems Corporation

Question No.3： Keywords should be better chosen. They should not contain words that are in the title of the manuscript;
Response to the question No.3: Thanks for your question. As your suggestion, the Keywords were revised as following:"distributed lag nonlinear models, climatic factors, effect".
Question No.4：There are several manuscripts addressing the theme "meteorological factors on mumps". In a quick search (https://www.sciencedirect.com/search/advanced?qs=meteorological%20factors%20mumps) I found at least seven similar manuscripts. Therefore, the authors should make clear in the Introduction what is the difference in the work done for papers that are already published. This needs to be improved in the Introduction;
Response to the question No.4: Thanks for your question. As your suggestion, we described the difference of the similar researches on mumps, and the section were revised as L68-86.
Question No.5： In the Material and Methods is necessary a map of the study area location;
Response to the question No.5: The map of the study area location was added in the Material and Methods.
Question No.6： Although the authors employed robust statistical analyzes, further testing is still needed to improve the discussion of results. As the authors worked with an 18-year time series, it is necessary to check for changes in this time series with the nonparametric Man-Kendall and Pettitt tests. These tests are easily performed in the software R and may show if there was a tendency to increase or decrease in any meteorological variable and in the cases;
Response to the question No.6: We use the nonparametric Man-Kendall and Pettitt tests to check for changes in our time series data. And the result was shown as Table 1
Question No.7：Table 1 is important, but I would like to see a boxplot for these variables. Use the ggplot2 package;
Response to the question No.7：Thanks for your suggestion. We used the boxplot (Fig 3.) to describe these meteorological variables instead of the original table.
Question No.8：It is necessary to inform which test applied the Sperman correlations. In addition, I invite the authors to graphically express the results in Table 2 through the correlation network. It is a didactic way of representing this matrix. Can be done with the "qgraph" package in R;
Response to the question No.8: Thanks for your suggestion. We applied the correlation network(Fig 5.)to graphically express the results in the original Table 2.
Question No.9：It is necessary to inform in the legend of Figures 2, 3 and 4 if the adjusted models were significant and which test was used for this.
Response to the question No.9: Based on the AICs, we selected the smallest AIC to different meteorological variables to structure the best models. We showed the RR and 95% confidence interval (95%CI) of the original figure 2，3 and 4. Meantime, we could see the RR and 95% CI in the original figure 3 and 4 (The red lines represent mean relative risks and the grey regions represent 95%CIs.) If the 95% CI include "1" as the RR value, there is not significant difference. Otherwise, it means that it's significantly different. It equals to the P value <0.05.
Question No.10：Discussion is very poor and needs to be improved. Authors should include findings from recent manuscripts I mentioned;
Response to the question No.10: Thanks for your advice. We got some recent manuscripts the reviewer mentioned and other new article into reference added to 43 articles, and revised the discussion.
Question No.11：In the conclusions, it is necessary to inform readers about the scientific advances that this manuscript has obtained in relation to the works already published. What are the proposals to minimize the occurrence of mumps in children by knowing the behavior of meteorological variables?
Response to the question No.11:
In the conclusions, scientific advance was informed as L358-361 "In general, compared to previous studies that focused on the effect of meteorological factors on mumps incidence, for the first time, we identified a significant nonlinear association between certain climatic variables and mumps incidence among children in Guangzhou with lag effects." And L363-365"Moreover, extreme effects of the variables mentioned above among mumps cases were detected in a subgroup analysis according to gender and age." Due to the results of our study, some proposals were recommended to minimize the occurrence of mumps among children. L367-371 "For

Powered by Editorial Manager® and ProduXion Manager® from Aries Systems Corporation

example, preventative measures and health education should be strengthened in the temperature range of 10–23.6ºC, and proper ventilation might be helpful in protecting children against infection with mumps. We also implied that children should cut down on going to crowded places such as playgrounds in the active period of mumps from May to July in Guangzhou."

Question No.12：The authors used only 35 references and most of them are old. In addition to being very little, recent references need to be included to demonstrate to readers that while there are many published manuscripts, the topic still needs to be studied.

Response to the question No.12: Thanks for your kindly reminder, We reviewed the recent references and revised the introduction, and demonstrated the scientific advance and the difference between our study and previous researches. Firstly our study use the DLNMs to explore the nonlinear relationships with lag effects between meteorological variables and mumps cases. Secondly we firstly used the DLNMs to analyze the meteorological effects on mumps cases among children. Finally, we analyze the effect by subpopulation by sex and age among children. This detail could be seen in the "introduction and discussion".

"

Reviewer #1:

The subject of the manuscript is interesting and topical, especially for the return of diseases previously eradicated or controlled in the last century. However, I found several weaknesses in the manuscript, for example, presentation of the results needs to improve, the discussion of the results does not cover other continents, only being restricted to Asia and Europe. As well as several gross errors throughout the manuscript, implying that the manuscript was not revised correctly. It is necessary to improve the quality of the figures to make them self explanatory. It is necessary to improve the conclusions of the manuscript.

Response to the question of reviewer 1：Thanks for your kindly suggestion. We review the recent article and add some researches from Canada and Mexico into the discussion. We reviewed English by Editage company to avoid gross errors in our article. We also revised and improved the conclusions of the manuscript, the scientific advance was mentioned in the conclusion(L358-361, L363-365), and the control measures to reduce the occurrence of mumps among children were recommended too(L367-371).

Reviewer #2:

The manuscript makes an interesting based on interaction between mumps and meteorological variables. It is believed that more information on the subject can still be extracted, even in face of the limitations described by authors. On this aspect, some points need to be improved throughout the text, I believe they should include the references of R software, as well as dlnm package, both described on manuscript. The introduction and materials and methods are well described. Regarding the results, I have some suggestions for improvement:

Question No.1：Replacement of Figure 1 with a boxplot of mumps cases on an annual and monthly basis.

Response to the Question No.1：Thanks for your suggestion, we replaced the figure 1 with a boxplot of mumps cases on annual and monthly basis(Fig 2.).

Question No.2：

The other variables would be displayed in Figure 2, with monthly boxplots, making the visualization of the results much more understandable in some passages of the text, exploring the maximum and minimum.

Response to the Question No.2：Thanks for your suggestion, we replaced the figure 2 with monthly boxplots, exploring the maximum and minimum(Fig 3.).

Question No.3：

The old Figure 2 (which will be for Figure 3) could include the intensities, not only leaving the color red, this would show the interaction between them.

Response to the Question No.3：Thanks for your suggestion, we tried to change the old figure 3 with the intensities, however, we thought the original one would be better.

Question No.4：

Improve the image resolution of all images, as they are of low quality.

Response to the Question No.4：thanks for your suggestion, we fix the image resolution of all images due to the guideline in Plos One Journal.

Question No.5：

Paragraph preceding Figure 3, correct the pHa for hPa, on page 11. Well, from these

	improvements and implementations, the article will be able to be approved.<br>Response to the Question No.5：Thanks for your advice, We correct the pHa for hPa, on original page 11.
**Additional Information:**	
**Question**	**Response**
**Financial Disclosure**<br><br>Enter a financial disclosure statement that describes the sources of funding for the work included in this submission. Review the submission guidelines for detailed requirements. View published research articles from *PLOS ONE* for specific examples.<br><br>This statement is required for submission and **will appear in the published article** if the submission is accepted. Please make sure it is accurate.<br><br>**Unfunded studies**<br>Enter: *The author(s) received no specific funding for this work.*<br><br>**Funded studies**<br>Enter a statement with the following details:<br>• Initials of the authors who received each award<br>• Grant numbers awarded to each author<br>• The full name of each funder<br>• URL of each funder website<br>• Did the sponsors or funders play any role in the study design, data collection and analysis, decision to publish, or preparation of the manuscript?<br>• **NO** - Include this sentence at the end of your statement: *The funders had no role in study design, data collection and analysis, decision to publish, or preparation of the manuscript.*<br>• **YES** - Specify the role(s) played.<br><br>* typeset	This study was supported by the Medical Health Technology Project for Guangzhou (20181A011051) and the Science and Technology Project of Guangzhou (201804010093) and the Project for Key Medicine Discipline Construction of Guangzhou Municipality (2017-2019-07).
**Competing Interests**<br><br>Use the instructions below to enter a competing interest statement for this submission. On behalf of all authors, disclose any competing interests that could be perceived to bias this work—acknowledging all financial support	There is no conflict of interest for the authors.

and any other relevant financial or non-financial competing interests.<br><br>This statement **will appear in the published article** if the submission is accepted. Please make sure it is accurate. View published research articles from *PLOS ONE* for specific examples.<br><br>**NO authors have competing interests**<br><br>Enter: *The authors have declared that no competing interests exist.*<br><br>**Authors with competing interests**<br><br>Enter competing interest details beginning with this statement:<br><br>*I have read the journal's policy and the authors of this manuscript have the following competing interests: [insert competing interests here]*<br><br>* typeset	
**Ethics Statement**<br><br>Enter an ethics statement for this submission. This statement is required if the study involved:<br><br>• Human participants<br>• Human specimens or tissue<br>• Vertebrate animals or cephalopods<br>• Vertebrate embryos or tissues<br>• Field research<br><br>Write "N/A" if the submission does not require an ethics statement.<br><br>General guidance is provided below. Consult the submission guidelines for detailed instructions. **Make sure that all information entered here is included in the Methods section of the manuscript.**	This work was approved by the Ethics Committee of the Guangzhou Center for Disease Control and Prevention.<br>All data were fully anonymized before we accessed them.

**Format for specific study types**<br><br>**Human Subject Research (involving human participants and/or tissue)**<br>• Give the name of the institutional review board or ethics committee that approved the study<br>• Include the approval number and/or a statement indicating approval of this research<br>• Indicate the form of consent obtained (written/oral) or the reason that consent was not obtained (e.g. the data were analyzed anonymously)<br><br>**Animal Research (involving vertebrate animals, embryos or tissues)**<br>• Provide the name of the Institutional Animal Care and Use Committee (IACUC) or other relevant ethics board that reviewed the study protocol, and indicate whether they approved this research or granted a formal waiver of ethical approval<br>• Include an approval number if one was obtained<br>• If the study involved *non-human primates*, add *additional details* about animal welfare and steps taken to ameliorate suffering<br>• If anesthesia, euthanasia, or any kind of animal sacrifice is part of the study, include briefly which substances and/or methods were applied<br><br>**Field Research**<br><br>Include the following details if this study involves the collection of plant, animal, or other materials from a natural setting:<br>• Field permit number<br>• Name of the institution or relevant body that granted permission	
**Data Availability**<br><br>Authors are required to make all data underlying the findings described fully available, without restriction, and from the time of publication. PLOS allows rare exceptions to address legal and ethical concerns. See the PLOS Data Policy and FAQ for detailed information.	Yes - all data are fully available without restriction

A Data Availability Statement describing where the data can be found is required at submission. Your answers to this question constitute the Data Availability Statement and **will be published in the article**, if accepted.<br><br>**Important:** Stating 'data available on request from the author' is not sufficient. If your data are only available upon request, select 'No' for the first question and explain your exceptional situation in the text box.<br><br>Do the authors confirm that all data underlying the findings described in their manuscript are fully available without restriction?	
**Describe where the data may be found in full sentences. If you are copying our sample text, replace any instances of XXX with the appropriate details.**<br><br>• If the data are **held or will be held in a public repository**, include URLs, accession numbers or DOIs. If this information will only be available after acceptance, indicate this by ticking the box below. For example: *All XXX files are available from the XXX database (accession number(s) XXX, XXX.).*<br>• If the data are all contained **within the manuscript and/or Supporting Information files**, enter the following: *All relevant data are within the manuscript and its Supporting Information files.*<br>• If neither of these applies but you are able to provide **details of access elsewhere**, with or without limitations, please do so. For example:<br><br>*Data cannot be shared publicly because of [XXX]. Data are available from the XXX Institutional Data Access / Ethics Committee (contact via XXX) for researchers who meet the criteria for access to confidential data.*<br><br>*The data underlying the results presented in the study are available from (include the name of the third party*	All relevant data are within the manuscript and its Supporting Information files.

and contact information or URL).<br>• This text is appropriate if the data are owned by a third party and authors do not have permission to share the data.<br><br>* typeset	
Additional data availability information:	

Powered by Editorial Manager® and ProduXion Manager® from Aries Systems Corporation

Manuscript Click here to access/download;Manuscript;manuscript.doc

The role of meteorological factors on mumps incidence among children in Guangzhou, Southern China

Jian-yun Lu[a#], Zhi-cong Yang[b#], Meng-meng Ma[a], Zhou-bin Zhang[c*]

[a]**Department of Infectious Disease Control and Prevention, Guangzhou Center for Disease Control and Prevention, Baiyun District Qi De Road in Guangzhou, Guangdong Province, China**

[b] **Director, Guangzhou Center for Disease Control and Prevention, Baiyun District Qi De Road in Guangzhou, Guangdong Province, China**

[c] **Vice Director, Guangzhou Center for Disease Control and Prevention, Baiyun District Qi De Road in Guangzhou, Guangdong Province, China**

[*]Corresponding author:

Email: **zhoubinzhanggz@21cn.com**

Their contributions were the same and could be acted as co-first author

[*]Corresponding author at: Guangzhou Center for Disease Control and Prevention, Baiyun District Qi De Road in Guangzhou, Guangdong Province, 510440, China.

Email:zhoubinzhanggz@21cn.com

// Abstract

Mumps, a common childhood disease, has a high incidence in Guangzhou city, China. It has been proven that mumps is influenced by seasonality. However, the role of meteorological factors among children is yet to be fully ascertained. This study explored the association between meteorological factors and the incidence of mumps among children in Guangzhou. Distributed lag nonlinear models were used to evaluate the correlation between meteorological factors and the incidence of mumps among children from 2014–2018. The nonlinear lag effects of some meteorological factors were detected. Mean temperature, atmospheric pressure, and relative humidity were positively correlated with mumps incidence, contrary to that of wind speed. Extreme effects of temperature, wind speed, atmospheric pressure, and relative humidity on the incidence of mumps among children in Guangzhou were evaluated in a subgroup analysis according to gender and age. Our preliminary results offered fundamental information to better understand the epidemic trends of mumps among children to develop an early warning system, and strengthen the intervention and prevention of mumps.

Keywords: distributed lag nonlinear models, climatic factors, effect

Introduction

Mumps is a common childhood disease caused by the mumps virus that mostly affects children aged 5–9 years (1, 2). It manifests clinically as an inflammation of the parotid gland with precursory fever. Most cases are mild and self-limiting, but can result in some severe complications such as deafness, aseptic meningitis, and encephalitis (3). The incubation period of mumps is 12–24 days, with a median of 19 days (4). Mumps is not only

transmitted by droplet spread, but also via direct contact or contaminated fomites (1).

Mumps can be effectively prevented by vaccination. A sharp reduction in incidence was observed after routine vaccination (5); however, many countries, including France (6), Ireland (7), UK (8), and USA (9) have experienced resurgences of mumps and reported large scale outbreaks over the past decade. Since 2007, the mumps vaccine has been integrated into the National Immunization Program in China, and the measles, mumps, and rubella (MMR) or measles and mumps (MM) vaccine has been provided free of charge to children aged 18–24 months in Guangzhou since 2008 (10). However, the incidence rates of mumps in China rose to 33.9/100,000 and 35.6/ 100,000 during 2011–2012 (11); meanwhile, 10008 and 7856 mumps cases were reported in Guangzhou (10). A one dose vaccine program is unavailable to fully control the epidemic of mumps, although the immunization coverage rate is about 80% to 90% nationwide (12). The two doses vaccination program was recommended by the World Health Organization (13) and the polyvalent mumps vaccine was recommended in the USA (9). Hence, mumps is still a serious public health concern among children not only in Guangzhou, China, but also worldwide.

The incidence of mumps showed apparent seasonality in various areas, with the peak in late spring, early summer, and winter. Different seasonal distributions were even observed in southern and northern China (11, 12). This hinted that mumps incidence was influenced by seasonal undulation. Therefore, we hypothesized that meteorological factors might play a vital role in the spread of mumps. Recently, the effects of meteorological factors on some infectious diseases have been explored extensively as early warning signals of possible epidemics, such as those of hand, foot, and mouth disease (14), varicella (15), and influenza (16). Limited studies have been conducted to explore the association between meteorological factors and the incidence of mumps. A study using the ARIMAX model in Beijing, China showed that increasing temperature would cause an increase in the incidence of mumps (17),

whereas a study that used a generalized additive model in Jining showed that an increasing trend in the incidence of mumps was observed when the temperature rose above 4ºC (18). However, a study which applied Pearson's correlation analysis in Czech Republic showed that there was no relationship between mumps incidence and drier and warmer weather (19). Besides, a Poisson regression model was applied in a study on mumps in 10 cities in Guangxi Province (20). However, most of the previous studies only focused on linear effects but not lag effects. A study conducted in Shenzhen city applied the distributed lag nonlinear model (DLNM) in exploring the relationship between wind velocity and mumps cases (21). Studies conducted in Fujian Province and Guangzhou city applied the DLNM in exploring the nonlinear correlations between meteorological factors and the incidence of mumps (22, 23). However, the nonlinear and lag effects of climatic factors on the transmission among children, who are the main population affected by mumps, are still unclear at present. A study conducted in Japan explored the linear relationship between meteorological factors and mumps incidence among children via negative binomial regression analysis (24). What are the roles of meteorological factors on mumps incidence among children? There is an urgent need to explore these nonlinear relationships using lag effects.

In this study, an ecological methodology was used to analyze the features of mumps in Guangzhou from 2014 to 2018. We built DLNMs (25), which can flexibly depict the nonlinear correlation and describe delayed effects in time-series studies, to analyze the effects of meteorological factors on mumps transmission among children. The findings of our study may be helpful in developing an early warning system for mumps epidemics among children, improving preventative measures, and reducing the risk of mumps infection among children.

Materials and methods

Study region

Guangzhou is the third largest city in China with a total of 8.97 million registered inhabitants. It is a typical meteorologically humid subtropical area and is located within 112°57′E to 114°3′E and 22°26′N to 23°56′N (**Fig 1)**. In Guangzhou, the summer ~~season~~ is wet and hot, whereas the winter ~~season~~ is dry and cool.

Fig 1. The geographical location of study area in Guangzhou city, Southern China.

Data sources

In this study, clinically diagnosed cases of mumps among persons younger than 18 years were obtained from the National Notifiable Disease Report System from January 2014 to December 2018. Clinically, mumps was defined as an acute onset of bilateral or unilateral tender, self-limited swelling of the parotid or other salivary glands lasting for more than 2 days. Mumps is a notifiable disease in Guangzhou; consequently, all clinicians are required to compulsorily report cases to local health authorities within 24 hours via the National Notifiable Disease Surveillance System.

Daily meteorological data, including mean temperature (°C), diurnal temperature range (°C) (highest temperature minus lowest temperature), atmospheric pressure (hPa), aggregate rainfall intensity (mm), wind speed (m/s), relative humidity (%), and sunshine duration (hours) were collected simultaneously from the Guangzhou Meteorological Bureau.

Statistical analysis

We performed a descriptive analysis of meteorological variables and the occurrence of mumps in Guangzhou. Based on scatter plots, we concluded that the correlations between the meteorological factors and mumps incidence were nonlinear. The Mann–Kendall and Pettitt tests were applied to initially analyze the tendency of the meteorological variables and mumps cases among children. Most of the variables tended to increase or decrease. Due to

the reasons mentioned above, DLNMs were applied to detect the relationships between meteorological variables and the occurrence of mumps. Atmospheric pressure was strongly correlated with average temperature (r=-0.82, P<0.01) as per the Spearman's correlation analysis. Hence, the two variables were not combined in a model to avoid the potential problem of collinearity. Poisson regression, performed with the quasi-Poisson function, was used to assess over-dispersion of daily cases of mumps in the DLNMs. The calculation is performed using the following equation:

$$\log[E(Y_t)] = \alpha + \sum NS_i\,(X_i, df_i) + \delta Dow + \delta Holiday_t + NS(Time, 7 \text{ per year}),$$

where Y_t represents the number of cases of mumps reported on day t; both α and δ are the intercepts; NS represents natural cubic spline; X represents meteorological variables, such as temperature, diurnal temperature range, rainfall, atmospheric pressure, wind speed, relative humidity, and sunshine hours; df is the degrees of freedom; Holiday is defined as a binary variable (if day t is a public holiday or school vacation, it is marked as 1, otherwise it is marked as 0); Dow represents the day of week effect; and Time denotes long term trends and seasonality. We selected the df of day to control long term trends, seasonality, and meteorological variables using the Akaike information criterion (AIC). Based on the AICs, we chose df=3 for NS according to the meteorological variables mentioned above. After combining the incubation period of mumps, df=24 was chosen for mean temperature, atmospheric pressure, and wind speed, and df=14 was chosen for relative humidity.

Extreme high effects were defined as when the value of meteorological variables were beyond the 97.5th percentile; when the value was below the 2.5th percentile, it was defined as an extreme low effect.

All statistical tests were two-sided. Analysis items with P<0.05 were considered statistically significant. All statistical analyses were performed using R version 3.5.3 (The R Development Core Team, Vienna, Austria) and the DLNMs were built using the *dlnm*

package in the R environment.

We performed the sensitivity analyses by shifting the df (5–9) per year to control for seasonality and long-term trends using time, and changing the df (4–7) for mean temperature, atmospheric pressure, diurnal temperature range, wind speed, accumulated rainfall, relative humidity, and sunshine hours.

Ethical considerations

The study protocol was approved by the Ethics Committee of the Guangzhou Center for Disease Control and Prevention. All data were fully anonymized before we accessed them.

Results

From January 1st, 2014 to December 31st, 2018, a total of 9842 mumps cases among persons younger than 18 years were reported in Guangzhou. During 2014 to 2018, the breakdown of mumps cases was 1962, 2077, 1838, 1977, and 1988, respectively. The gender ratio was 1.83 (6362 males to 3480 females). Among the groups aged 0–3, 4–6, 7–14, and 15–17 years, there were 2197 (22.32%), 3773 (38.34%), 3672 (37.31%), and 200 (2.03%) cases, respectively. The peak of mumps occurrence in the study period occurred from May to July, with a total of 3087 cases (31.37%) **(Fig 2)**.

Fig 2. The boxplot of mumps cases among children in Guangzhou, 2014–2018.

A description of meteorological variables and mumps cases is given as follows **(Fig 3)**. The mean daily temperature was 22.24°C, and the means of diurnal temperature, aggregate rainfall, atmospheric pressure, wind speed, relative humidity, and hours of sunshine were 8.20°C, 6.04 mm, 1005.41 hPa, 2.16 m/s, 79.19%, and 4.42 hours, respectively. The monthly distribution is shown in the monthly boxplot of meteorological factors **(Fig 4)**. The results of

the Mann–Kendall and Pettitt tests are shown in Table 1. Except for cases, temperature and sunshine hours, most of the variables tended to increase or decrease.

Fig 3. The boxplot of meteorological factors and mumps cases among children in Guangzhou, 2014–2018.

Fig 4. The monthly boxplot of meteorological factors in Guangzhou, 2014–2018.

Table 1. The results of the Man-Kendall and Pettitt tests for the meteorological variables and mumps cases among children in Guangzhou, 2014-2018

variables	P	S	change-point number	change-point	P
case	0.3	+	1542	2018/3/22	<**0.01**
temperature	0.19	+	129	2014/5/9	<**0.01**
DTR	<**0.01**	-	414	2015/2/18	<**0.01**
rain	<**0.01**	-	986	2016/9/12	<**0.01**
pressure	<**0.01**	-	482	2015/4/27	<**0.01**
wind	<**0.01**	+	371	2015/1/6	<**0.01**
relative humidity	<**0.01**	+	794	2016/3/4	<**0.01**
Sunshine hours	0.88	-	674	2015/11/5	**0.02**

S: '+' means a rise tendency , '-'means a decrease tendency

Fig 5 shows the correlation network between meteorological factors and mumps cases among children. Spearman's correlation analyses revealed that the incidence of mumps was positively correlated with mean temperature, relative humidity, aggregate rainfall, and hours of sunshine. On the contrary, wind speed and atmospheric pressure were negatively correlated with the incidence of mumps. Mean temperature was strongly correlated with atmospheric pressure (r=-0.82, P<0.01).

Fig 5. The correlation network between meteorological factors and mumps cases among children in Guangzhou, 2014–2018.

*cas represents cases; tm represents temperature; prs represents atmospheric pressure;

wnd represents wind; dtr represents diurnal temperature range; ran represents rain; rhm represents relative humidity; sun represents sunshine hours. The green line means positive relationship, whereas the red one means negative relationship. The width of the lines dedicates the degree of *r.* The thicker the line, the greater the correlation.

Fig 6 illustrates the correlation between mumps cases and meteorological variables with different lag periods in Guangzhou. Specific features were observed in various variables with a nonlinear curve. The highest relative risk (RR) was 1.005 (95% confidence interval [CI]: 1.001–1.010) at lag day 21, when the daily mean temperature was 25.0ºC. When atmospheric pressure was 1027 hPa at lag day 0, the lowest RR was 0.775 (95% CI: 0.655–0.916). When relative humidity was 72.0%, the highest RR was 1.014 (95% CI: 1.003–1.026) at lag day 12. When wind velocity was 8.70 m/s, the highest RR was 1.269 (95% CI: 1.033–1.560) at lag day 11.

Fig 6. Plots of the relative risk of climatic factors on the incidence of mumps in children, including mean temperature, diurnal temperature range, atmospheric pressure, wind speed, relative humidity and sunshine hours.

For a better explanation, the one-dimensional curves were plotted about the overall effects of meteorological factors over the corresponding lag days (**Fig 7)**. In a word, mean temperature, atmospheric pressure, and relative humidity were associated with mumps incidence, unlike wind speed. The RR of mean temperature significantly increased from 10ºC to 23.6ºC, whereas that of wind speed significantly declined from 1.9 to 4.5 m/s. Atmospheric pressure and relative humidity both showed a reverse U-shaped curve relationship with mumps. However, the RRs just had a significantly increasing trend before 992 hPa and 60%, respectively. There were no significant relationships between other meteorological variables and the incidence of mumps in our study.

Fig 7. The estimated overall effects of mean temperature, diurnal temperature range, atmospheric pressure, wind speed, relative humidity, and sunshine hours along corresponding lag days. The Y lab represents the value of relative risk, the X lab represents the value of relevant variables. The red lines represent mean relative risks and the grey regions represent 95% CIs.The black vertical line represents the medians of the climatic factors, and the dotted lines represent the 2.5 percentile and the 97.5 percentile for the meteorological factors, respectively.

The cumulative extreme effects of meteorological factors on mumps along the corresponding lag days are shown in **Fig 8**. As shown in **Table 2,** the extreme low effect of temperature was significant and the lowest RR was 0.88 (95% CI: 0.83–0.93) at lag day 0, whereas the extreme high effect of temperature was not significant. On the contrary, the extreme high effects of atmospheric pressure and wind speed were statistically significant, whereas the extreme low effects were not. For atmospheric pressure, the lowest RR was 0.88 (95% CI: 0.82–0.94) at lag day 0. For wind speed, the lowest RR was 0.94 (95% CI: 0.89–0.98) at lag day 0. Regarding relative humidity, the RR of the extreme high effect was 0.96 (95% CI: 0.94–0.99) at lag day 2, and the RR of the extreme low effect was 0.96 (95% CI: 0.93–0.99) at lag day 2. For the extreme high and low effects of sunshine hours, the RRs were 1.03 (95% CI: 1.02–1.04) at lag day 2 and 1.02 (95% CI: 1.01–1.03) at lag day 17, respectively.

Fig 8. The extreme effects of mean temperature, diurnal temperature range, atmospheric pressure, wind speed, relative humidity, and sunshine hours with extreme high effects (97.5%) and extreme low effects (2.5%).The Y lab represents the value of relative risk, the X lab represents the value of lag day. The red lines represent mean relative risks and grey regions represent 95%CIs.

Table 2

The extreme effects of meteorological variables on the incidence of mumps among children along the lag days in Guangzhou, 2014–2018.

Meteorological variables	Lag day	High effect (97.5%)		Lag day	Low effect (2.5%)	
		RR	95%CI		RR	95%CI
Mean temperature	/	/	/	0	0.88	0.83–0.93
Atmospheric pressure	0	088	0.82-0.94	/	/	/
Wind speed	0	0.94	0.89-0.98	/	/	/
Relatively humidity	2	0.96	0.94-0.99	2	0.96	0.93–0.99

In the analyses by subpopulation, the extreme low effects of temperature, atmospheric pressure, and relative humidity, and the extreme high effect of wind speed were statistically significant in the female population, but no significant extreme effect was observed in the male population. The extreme low effect of temperature was observed in the 4–6 year old group (RR=0.25, 95% CI: 0.11–0.60) and 15–17 year old group (RR=0.01, 95% CI: 0.00–0.57). The extreme high effect of atmospheric pressure was observed in the 4–6 year age group (RR=0.46, 95% CI: 0.21–0.98) **(Table 3)**.

Sensitivity analyses indicated similar results after the changes in the df for long-term trends and seasonality. Altering the dfs for temperature, atmospheric pressure, wind speed, and relative humidity, the original models showed better robustness compared to their alternatives.

Table 3

The cumulative extreme effects of meteorological variables on mumps cases of children by sex and age.

Varia bles	Mean temperature		Wind speed		Relative humidity		Atmospheric pressure	
	low	high	low	high	low	high	low	high

	effect		effect		effect		effect		effect		effect		effect		effect	
	RR	95% CI	RR	95% CI	RR	95% CI	RR	95% CI	RR	95% CI	RR	95% CI	RR	95% CI	RR	95% CI
total	**0.56**	**0.32-0.99**	0.99	0.70-1.40	0.77	0.47-1.28	0.64	0.40-1.01	**0.76**	**0.61-0.94**	0.89	0.67-1.17	**0.70**	**0.49-0.99**	0.75	0.45-1.24
male	0.88	0.44-1.73	0.91	0.59-1.39	0.76	0.41-1.41	0.88	0.50-1.56	0.95	0.73-1.23	1.06	0.75-1.49	0.87	0.56-1.34	0.81	0.43-1.50
female	**0.23**	**0.10-0.62**	1.12	0.66-2.02	0.77	0.74-1.71	**0.34**	**0.16-0.72**	**0.50**	**0.35-0.72**	0.64	0.41-1.00	**0.47**	**0.26-0.83**	0.64	0.28-1.45
< 4years	1.83	0.60-5.65	1.40	0.69-2.83	0.99	0.36-2.71	0.74	0.29-1.87	0.73	0.48-1.11	0.70	0.39-1.24	0.54	0.26-1.09	0.97	0.37-2.55
4-6 years	**0.25**	**0.11-0.60**	1.01	0.58-1.75	0.68	0.31-1.49	0.51	0.25-1.05	0.77	0.56-1.07	1.28	0.83-1.96	0.81	0.46-1.42	**0.46**	**0.21-0.98**
7-13 years	0.82	0.32-2.10	0.83	0.48-1.44	0.79	0.35-1.80	0.81	0.37-1.76	0.76	0.52-1.11	0.72	0.45-1.14	0.73	0.41-1.30	1.25	0.51-3.05
14-17 years	**0.01**	**0.00-0.57**	0.18	0.01-2.74	0.35	0.01-16.28	0.05	0.00-2.18	0.48	0.07-3.29	0.38	0.05-3.09	0.20	0.01-2.66	0.08	0.00-3.08

Discussion

In this study, the incidence of mumps among children in Guangzhou fluctuated seasonally with the epidemic peak from May to July. The trough of mumps incidence among children occurred in February, coinciding with the winter holiday in school. However, our result differed from that of a study conducted in Ireland, which detected a relatively low mumps incidence in summer and a peak in autumn (7), and a study conducted in the Netherlands with two seasonality peaks in spring and autumn (26). In China, the epidemic pattern in Guanghzou also differed from that of Beijing city, Zibo city in Shandong province, and Chongqing city, which showed two epidemic peaks from April to July and October to January, with an emphasis on the former (17, 27, 28). It indicated that the effect of meteorological factors on the occurrence of mumps may be distinct in various regions with particular climatic features.

Climatic variables such as temperature, relative humidity, and wind speed have been proven to play a key role in the occurrence of certain infectious diseases (29-31). Recently, more and more attention has been drawn to the effects of meteorological factors on mumps

incidence. Some studies have been conducted to explore the relationships between meteorological factors and mumps cases; however, most of the studies just detected linear relationships without lag effects. Recently, DLNMs have been applied widely to evaluate these nonlinear correlations with lag effects (10, 21, 23). However, these relationships among children still need to be explored.

To the best of our knowledge, this is the first study to apply DLNMs to detect the effects of meteorological variables on mumps incidence among children in a subtropical city in China. Nonlinear relationships were observed in some meteorological variables in our study. When the mean temperature ranged from 10–23.6ºC, the RR showed an increased trend. Our finding was not fully consistent with previous studies. A study conducted in Mexico showed that the mumps incidence declined markedly during the summer (32). However, a study conducted in Taiwan (33) reported that the incidence of mumps started to increase at a temperature of 20°C, and started to decline after 25°C, whereas a study conducted in Jining, China (18) and a study focused on children in Japan (24) both reported linearly positive associations between mumps incidence and temperature. A previous study conducted in Guangzhou (22) also revealed a positive correlation between mean temperature and the incidence of mumps in the overall population. However, another study conducted in Jining showed a U-shaped relationship curve between mean temperature and mumps incidence (34). The discrepancy may be due to the differences in methodology, geographic location, and climatic characteristics. The incidence of mumps depended on the contact rate, which might be influenced by social behavior (35). A previous epidemiologic study conducted in Canada showed that adolescents usually engage in more physical activity in the warmer months than in the colder months (36). Children are more likely to come into contact with each other in warmer weather, which might result in an increase in the incidence of mumps. When the temperature continued to increase, outdoor activities among children

might decrease, reducing the risk of mumps infection; however, the extreme high effect was not statistically significant. For the same reason, the risk of mumps infection will be reduced in extreme cold conditions; however, the accumulated extreme cold effect was only observed significantly among females. These might explain our finding among children in Guangzhou and shows that preventative measures and health education should be strengthened in the temperature range mentioned above in our study.

The results of some studies were inconsistent with ours. For example, relative humidity was found to have a negative impact on mumps incidence with a threshold of 54% in Jining, China (18). However, another study conducted in Jining showed that the effect of relative humidity on mumps increased slightly and then decreased quickly, with a threshold of 64% (34). Besides, a study conducted in Taiwan (33) found no correlation between relative humidity and mumps incidence. Our study showed a U-shaped relationship between relative humidity and mumps incidence among children in Guangzhou, with a statistically significant increase before a relative humidity of 60%. Meanwhile, nonlinear extreme high and low effects of relative humidity on mumps incidence were both found among children. Studies on the mechanism underlying the effect of relative humidity on mumps incidence are rare and should be carried out in the future. One possible mechanism might be as follows. Droplet spread is one of the modes of transmission of the mumps virus (1). When the relative humidity is low, water in the droplets evaporates quickly. The longer the droplet with mumps viruses remains airborne, the easier it is the mumps infect the children (37). As the relative humidity increases, the effects become insignificant. However, an extremely high effect of relative humidity was observed with an RR of 0.96 (95% CI: 0.93–0.99) at lag day 2, which may be explained by the fact that high relative humidity often coincides with heavy rain, which will make children more likely to stay at home (38) and reduce the contact rate. More studies should be carried out to identify biological evidence.

Wind could affect the suspension time of viruses and their diffusion distance (39, 40). Our study revealed that the RR decreased as the wind speed surpassed 1.9 m/s till 4.9 m/s. Moreover, the high effect of wind speed yielded an RR of 0.94 (95% CI: 0.89–0.98). Our study result was similar to that of a study conducted in Shenzhen, which depicted a reverse V-shaped relationship between wind velocity and mumps incidence, with the peak at 2 m/s (21), and a study conducted in Fujian province (23), which showed a negative relationship between wind speed and mumps incidence. In view of the resuspension phenomenon, the concentration of local viruses may be diluted by higher wind velocity (39, 41), which could possibly explain our finding. In the subpopulation analysis, an accumulated high effect of wind speed was observed among females. This may be explained by the reduction in outdoor activity among females in the extreme high wind velocity weather. Based on our findings, regular ventilation might be helpful in protecting children against infection with mumps.

In our study, extreme low effects of mean temperature, atmospheric pressure, and relative humidity, and an extreme high effect of wind speed were found among females with RR values less than 1, whereas no extreme effect was detected among males. This may be due to factors such as physiological gender differences and the discrepancy in behavior patterns (42). For example, in extreme weather conditions, males are more active and will be more likely to play with friends, which will increase the contact rate with children infected with mumps. This shows that children should cut down on going to crowded places such as playgrounds in the active period of mumps from May to July in Guangzhou.

Our results showed a positive correlation before 1000.05 hPa, after which point the correlation was not statistically significant. However, previous studies showed different results. For instance, a study conducted in 10 cities in Guangxi province in China (20) reported that no significant relationship was found between atmospheric pressure and the incidence of mumps, whereas a study conducted in Beijing (17) showed that atmospheric

pressure was negatively associated with mumps incidence. Unfortunately, limited studies are available regarding the underlying mechanism. This may be explained as follows. As the atmospheric pressure increases in the beginning, the cold air would enter and promote the spread of the virus, leading to high virus abundance in the atmosphere. After exceeding a certain value of atmospheric pressure, the effect mentioned above is offset by high wind speed. However, further studies should be conducted to explore the foundational mechanism in future.

Several limitations of our study should be acknowledged. At first, the data were only obtained in Guangzhou, southern China; hence, it cannot stand for other cities or countries. Secondly, we used meteorological data instead of the exposure conditions of individuals; this may lead to bias in our results. Further, we could not exclude the possibility that some potential confounding factors existed in our research, including host susceptibility, preventive measures, geographic factors, and population density. Moreover, data regarding human serum antibodies against mumps and information regarding mumps vaccination were unavailable in our study. Finally, we did not assess factors regarding air pollution which have been proven to affect the incidence of mumps (43). Therefore, more studies should be undertaken to take these variables into consideration.

Conclusions

In general, compared to previous studies that focused on the effect of meteorological factors on mumps incidence, for the first time, we identified a significant nonlinear association between certain climatic variables and mumps incidence among children in Guangzhou with lag effects. At a certain corresponding range, mean temperature, atmospheric pressure, and relative humidity were positively correlated with the incidence of mumps among children, opposite to that of wind speed. Moreover, extreme effects of the

variables mentioned above among mumps cases were detected in a subgroup analysis according to gender and age. These findings provide the underlying information to better understand the epidemic tendency of mumps. Based on our results, some measures could be implemented to minimize the occurrence of mumps. For example, preventative measures and health education should be strengthened in the temperature range of 10–23.6ºC, and proper ventilation might be helpful in protecting children against infection with mumps. We also implied that children should cut down on going to crowded places such as playgrounds in the active period of mumps from May to July in Guangzhou. Our findings in this study will help improve the early warning system and reinforce the prevention and intervention of risk factors for mumps among children in Guangzhou.

Acknowledgments

We thank the Guangzhou Meteorological Bureau for offering climatic data for our study. We would like to thank Editage (www.editage.cn) for English language editing.

Supporting information files

The data and the R program of our study were uploaded as the supporting information file, which also included the data of subpopulation analyses.

References

1. Hviid A, Rubin S, Muhlemann K. Mumps. Lancet (London, England). 2008;371(9616):932-44.https://doi.org/10.1016/s0140-6736(08)60419-5.

2. Rubin S, Eckhaus M, Rennick LJ, Bamford CG, Duprex WP. Molecular biology, pathogenesis and pathology of mumps virus. The Journal of pathology. 2015;235(2):242-52.https://doi.org/10.1002/path.4445.

3. Grennan D. Mumps. Jama. 2019;322(10):1022.https://doi.org/10.1001/jama.2019.10982.

4. Richardson M, Elliman D, Maguire H, Simpson J, Nicoll A. Evidence base of incubation periods, periods of infectiousness and exclusion policies for the control of communicable diseases in schools and preschools. The Pediatric infectious disease journal. 2001;20(4):380-

91.https://doi.org/10.1097/00006454-200104000-00004.

5. Galazka AM, Robertson SE, Kraigher A. Mumps and mumps vaccine: a global review. Bulletin of the World Health Organization. 1999;77(1):3-14

6. Vygen S, Fischer A, Meurice L, Mounchetrou Njoya I, Gregoris M, Ndiaye B, et al. Waning immunity against mumps in vaccinated young adults, France 2013. Euro Surveill. 2016;21(10):30156.https://doi.org/10.2807/1560-7917.es.2016.21.10.30156.

7. Whyte D, O'Dea F, McDonnell C, O'Connell NH, Callinan S, Brosnan E, et al. Mumps epidemiology in the mid-west of Ireland 2004-2008: increasing disease burden in the university/college setting. Euro Surveill. 2009;14(16)

8. Aasheim ET, Inns T, Trindall A, Emmett L, Brown KE, Williams CJ, et al. Outbreak of mumps in a school setting, United Kingdom, 2013. Hum Vaccin Immunother. 2014;10(8):2446-9.https://doi.org/10.4161/hv.29484.

9. May M, Rieder CA, Rowe RJ. Emergent lineages of mumps virus suggest the need for a polyvalent vaccine. International Journal of Infectious Diseases. 2018;66:1-4.https://doi.org/https://doi.org/10.1016/j.ijid.2017.09.024.

10. Fu C, Xu J, Cai Y, He Q, Zhang C, Chen J, et al. Effectiveness of one dose of mumps vaccine against clinically diagnosed mumps in Guangzhou, China, 2006-2012. Hum Vaccin Immunother. 2013;9(12):2524-8.https://doi.org/10.4161/hv.26113.

11. Su QR, Liu J, Ma C, Fan CX, Wen N, Luo HM, et al. Epidemic profile of mumps in China during 2004-2013. Zhonghua yu fang yi xue za zhi [Chinese journal of preventive medicine]. 2016;50(7):611-4.https://doi.org/10.3760/cma.j.issn.0253-9624.2016.07.009.

12. Cui A, Zhu Z, Hu Y, Deng X, Sun Z, Zhang Y, et al. Mumps Epidemiology and Mumps Virus Genotypes Circulating in Mainland China during 2013-2015. PLoS One. 2017;12(1):e0169561.https://doi.org/10.1371/journal.pone.0169561.

13. WHO. Mumps virus vaccines: WHO position paper. Wkly Epidemiol Rec. 2017;82:51-60

14. Yu G, Li Y, Cai J, Yu D, Tang J, Zhai W, et al. Short-term effects of meteorological factors and air pollution on childhood hand-foot-mouth disease in Guilin, China. Sci Total Environ. 2019;646:460-70.https://doi.org/10.1016/j.scitotenv.2018.07.329.

15. Yang Y, Geng X, Liu X, Wang W, Zhang J. Association between the incidence of varicella and meteorological conditions in Jinan, Eastern China, 2012-2014. BMC Infect Dis. 2016;16:179.https://doi.org/10.1186/s12879-016-1507-1.

16. Pan M, Yang HP, Jian J, Kuang Y, Xu JN, Li TS, et al. Association of meteorological factors with seasonal activity of influenza A subtypes and B lineages in subtropical western China. Epidemiol Infect. 2019;147:e72.https://doi.org/10.1017/s0950268818003485.

17. Hao Y, Wang RR, Han L, Wang H, Zhang X, Tang QL, et al. Time series analysis of mumps and meteorological factors in Beijing, China. BMC Infect Dis. 2019;19(1):435.https://doi.org/10.1186/s12879-019-4011-6.

18. Li R, Lin H, Liang Y, Zhang T, Luo C, Jiang Z, et al. The short-term association between meteorological factors and mumps in Jining, China. Sci Total Environ. 2016;568:1069-

75.https://doi.org/10.1016/j.scitotenv.2016.06.158.

19. Hubalek Z. North Atlantic weather oscillation and human infectious diseases in the Czech Republic, 1951-2003. European Journal of Epidemiology. 2005;20(3):263-70.https://doi.org/10.1007/s10654-004-6518-3.

20. Yu G, Yang R, Yu D, Cai J, Tang J, Zhai W, et al. Impact of meteorological factors on mumps and potential effect modifiers: An analysis of 10 cities in Guangxi, Southern China. Environmental research. 2018;166:577-87.https://doi.org/10.1016/j.envres.2018.06.047.

21. Zhou Z, Mei S, Chen J, Li X, Yin L, Liang J, et al. Nonlinear effect of wind velocity on mumps in Shenzhen, China, 2013–2016. Public health. 2020;179:178-85.https://doi.org/https://doi.org/10.1016/j.puhe.2019.10.023.

22. Yang Q, Yang Z, Ding H, Zhang X, Dong Z, Hu W, et al. The relationship between meteorological factors and mumps incidence in Guangzhou, China, 2005-2012. Hum Vaccin Immunother. 2014;10(8):2421-32.https://doi.org/10.4161/hv.29286.

23. Hu W, Li Y, Han W, Xue L, Zhang W, Ma W, et al. Meteorological factors and the incidence of mumps in Fujian Province, China, 2005-2013: Non-linear effects. Sci Total Environ. 2018;619-620:1286-98.https://doi.org/10.1016/j.scitotenv.2017.11.108.

24. Onozuka D, Hashizume M. Effect of weather variability on the incidence of mumps in children: a time-series analysis. Epidemiol Infect. 2011;139(11):1692-700.https://doi.org/10.1017/s0950268810002967.

25. Gasparrini A. Distributed Lag Linear and Non-Linear Models in R: The Package dlnm. J Stat Softw. 2011;43(8):1-20

26. Sane J, Gouma S, Koopmans M, de Melker H, Swaan C, van Binnendijk R, et al. Epidemic of mumps among vaccinated persons, The Netherlands, 2009-2012. Emerg Infect Dis. 2014;20(4):643-8.https://doi.org/10.3201/eid2004.131681.

27. Xu Q, Li R, Liu Y, Luo C, Xu A, Xue F, et al. Forecasting the Incidence of Mumps in Zibo City Based on a SARIMA Model. Int J Environ Res Public Health. 2017;14(8).https://doi.org/10.3390/ijerph14080925.

28. Zhu H, Zhao H, Ou R, Xiang H, Hu L, Jing D, et al. Epidemiological Characteristics and Spatiotemporal Analysis of Mumps from 2004 to 2018 in Chongqing, China. Int J Environ Res Public Health. 2019;16(17).https://doi.org/10.3390/ijerph16173052.

29. Kaffenberger BH, Shetlar D, Norton SA, Rosenbach M. The effect of climate change on skin disease in North America. Journal of the American Academy of Dermatology. 2017;76(1):140-7.https://doi.org/10.1016/j.jaad.2016.08.014.

30. Cheng Q, Bai L, Zhang Y, Zhang H, Wang S, Xie M, et al. Ambient temperature, humidity and hand, foot, and mouth disease: A systematic review and meta-analysis. Sci Total Environ. 2018;625:828-36.https://doi.org/10.1016/j.scitotenv.2018.01.006.

31. Peci A, Winter AL, Li Y, Gnaneshan S, Liu J, Mubareka S, et al. Effects of Absolute Humidity, Relative Humidity, Temperature, and Wind Speed on Influenza Activity in Toronto, Ontario, Canada. Applied and environmental microbiology. 2019;85(6).https://doi.org/10.1128/aem.02426-18.

32. Mahmud AS, Metcalf CJ, Grenfell BT. Comparative dynamics, seasonality in transmission, and predictability of childhood infections in Mexico. (1469-4409 (Electronic))

33. Ho YC, Su BH, Su HJ, Chang HL, Lin CY, Chen H, et al. The association between the incidence of mumps and meteorological parameters in Taiwan. Hum Vaccin Immunother. 2015;11(6):1406-12.https://doi.org/10.1080/21645515.2015.1029687.

34. Zhang D, Guo Y, Rutherford S, Qi C, Wang X, Wang P, et al. The relationship between meteorological factors and mumps based on Boosted regression tree model. Sci Total Environ. 2019;695:133758.https://doi.org/10.1016/j.scitotenv.2019.133758.

35. Phung D, Nguyen HX, Nguyen HLT, Luong AM, Do CM, Tran QD, et al. The effects of socioecological factors on variation of communicable diseases: A multiple-disease study at the national scale of Vietnam. PloS one. 2018;13(3):e0193246-e.https://doi.org/10.1371/journal.pone.0193246.

36. Belanger M, Gray-Donald K, O'Loughlin J, Paradis G, Hanley J. Influence of weather conditions and season on physical activity in adolescents. Ann Epidemiol. 2009;19(3):180-6.https://doi.org/10.1016/j.annepidem.2008.12.008.

37. Tang JW. The effect of environmental parameters on the survival of airborne infectious agents. J R Soc Interface. 2009;6 Suppl 6:S737-46.https://doi.org/10.1098/rsif.2009.0227.focus.

38. Guo Q, Dong Z, Zeng W, Ma W, Zhao D, Sun X, et al. The effects of meteorological factors on influenza among children in Guangzhou, China. Influenza and other respiratory viruses. 2019;13(2):166-75.https://doi.org/10.1111/irv.12617.

39. Zhong X, Qi, J., Li, H., Dong, L., & Gao, D. Seasonal distribution of microbial activity in bioaerosols in the outdoor environment of the Qingdao coastal region. Atmospheric Environment. 2016;140(06):506-13.https://doi.org/doi.org/10.1016/j.atmosenv.2016.06.034.

40. Jones AM, Harrison RM. The effects of meteorological factors on atmospheric bioaerosol concentrations--a review. Sci Total Environ. 2004;326(1-3):151-80.https://doi.org/10.1016/j.scitotenv.2003.11.021.

41. Sabariego S, Diaz de la Guardia C, Alba F. The effect of meteorological factors on the daily variation of airborne fungal spores in Granada (southern Spain). Int J Biometeorol. 2000;44(1):1-5

42. Yu L, Winter S, Xie D. The child play behavior and activity questionnaire: a parent-report measure of childhood gender-related behavior in China. Archives of sexual behavior. 2010;39(3):807-15.https://doi.org/10.1007/s10508-008-9403-4.

43. Hao J, Yang Z, Huang S, Yang W, Zhu Z, Tian L, et al. The association between short-term exposure to ambient air pollution and the incidence of mumps in Wuhan, China: A time-series study. Environmental research. 2019;177:108660.https://doi.org/10.1016/j.envres.2019.108660.

Fig 1

Click here to access/download;Figure;Fig1.tif

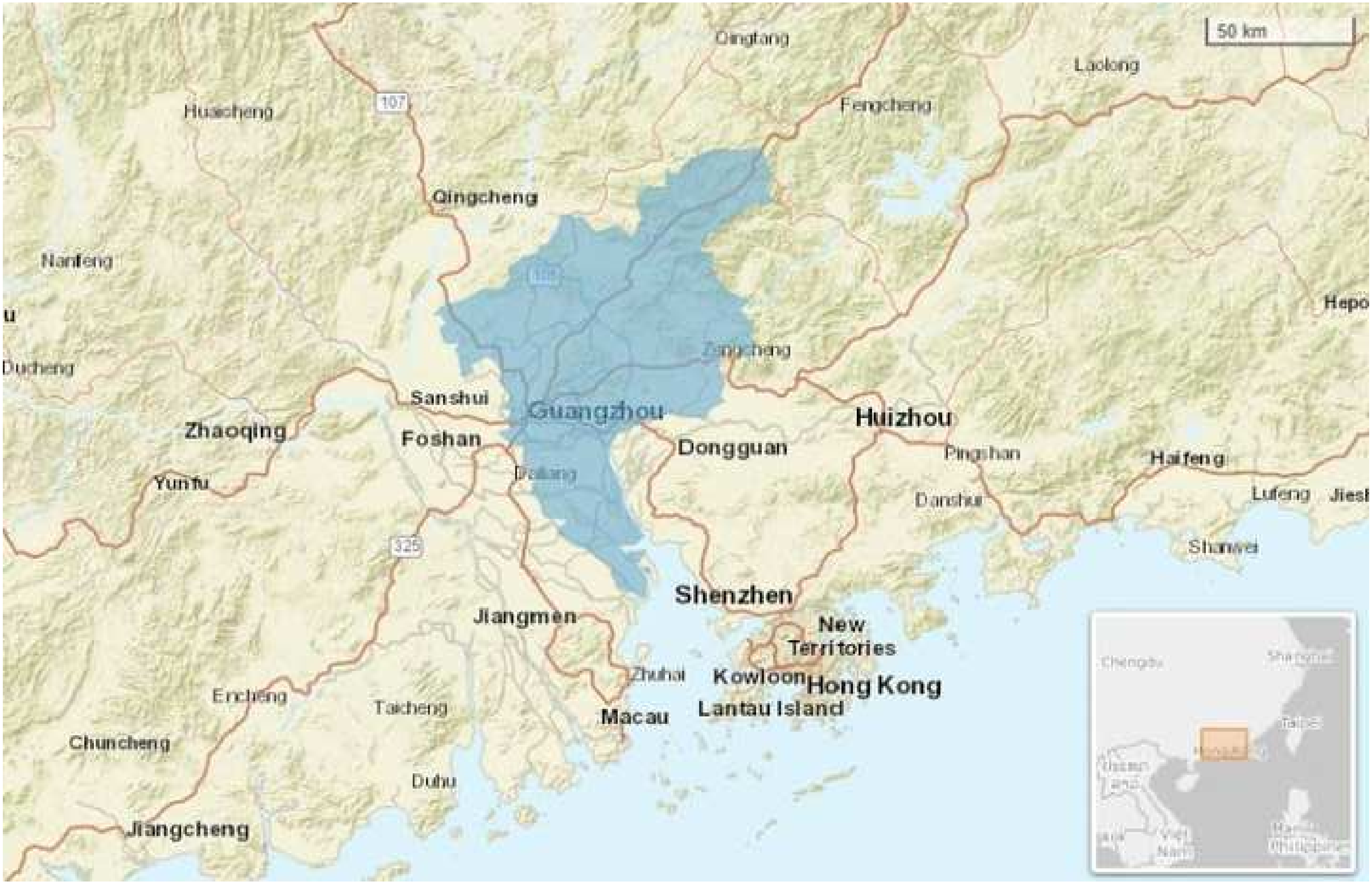

Fig 2

Click here to access/download;Figure;Fig 2.tif

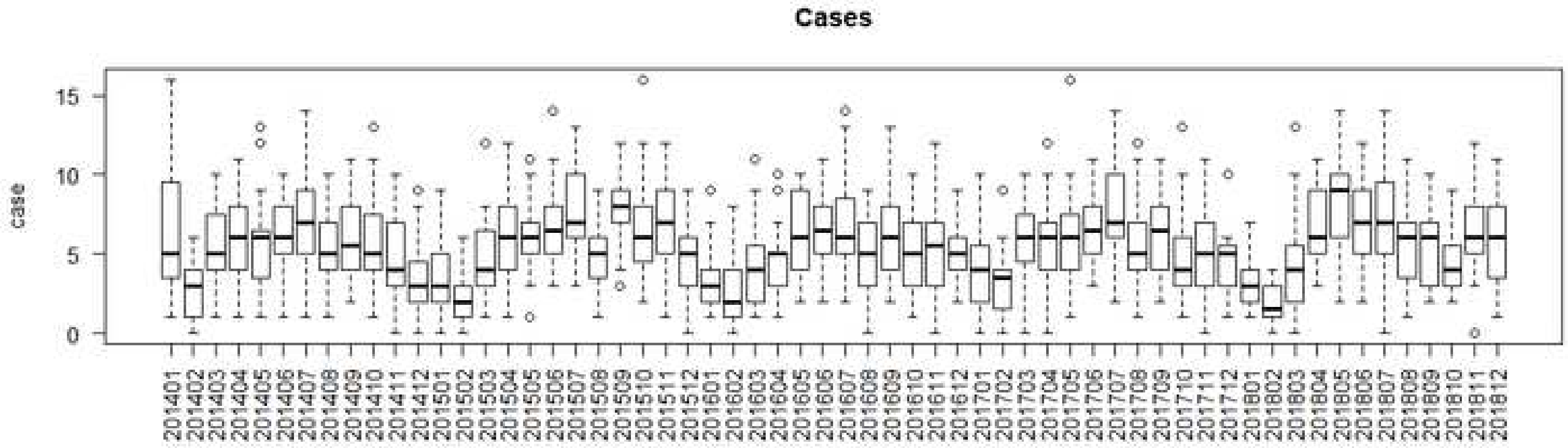

Fig 3

Click here to access/download;Figure;Fig 3.tif

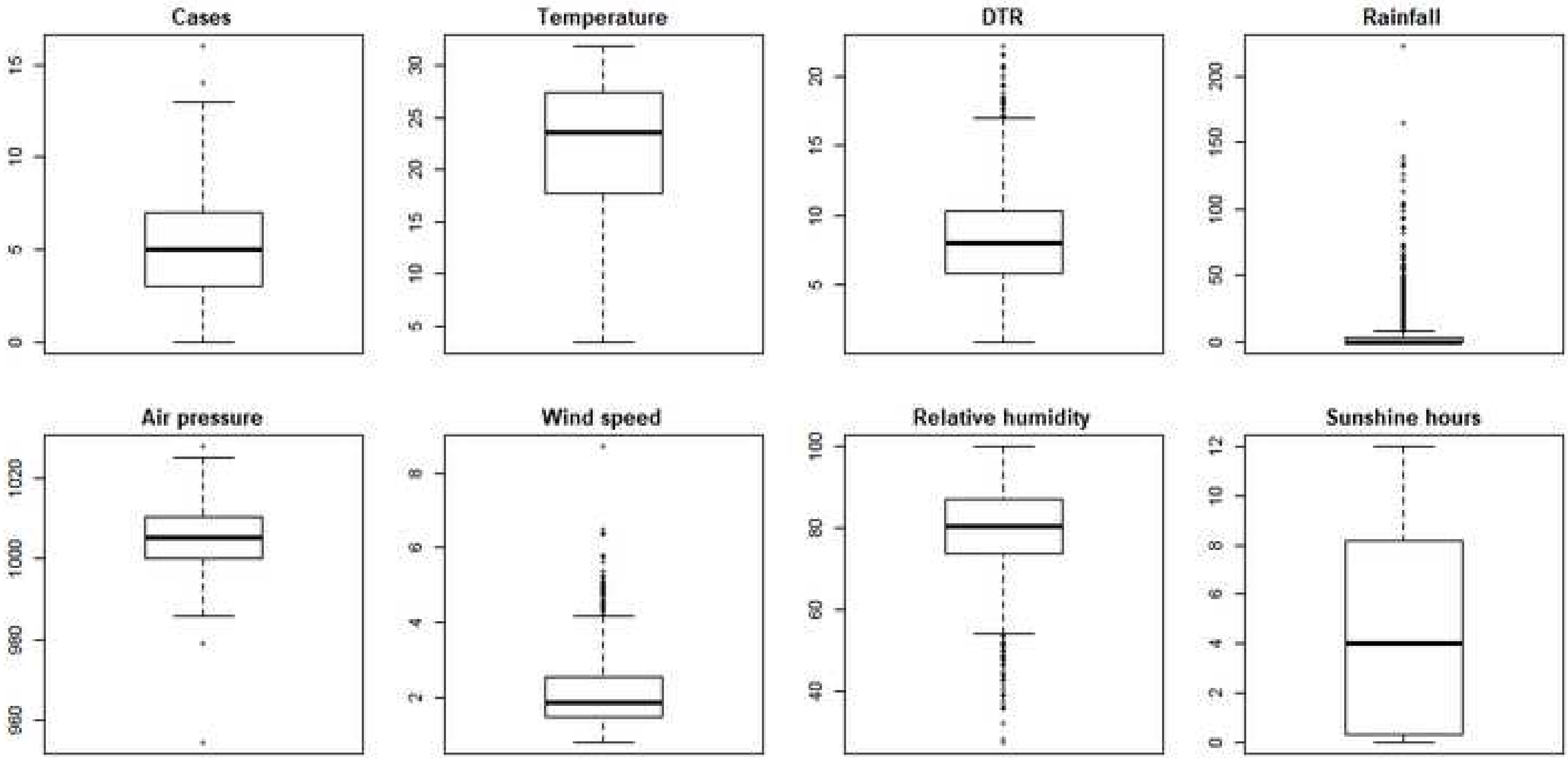

Fig 4

Click here to access/download;Figure;Fig 4.tif

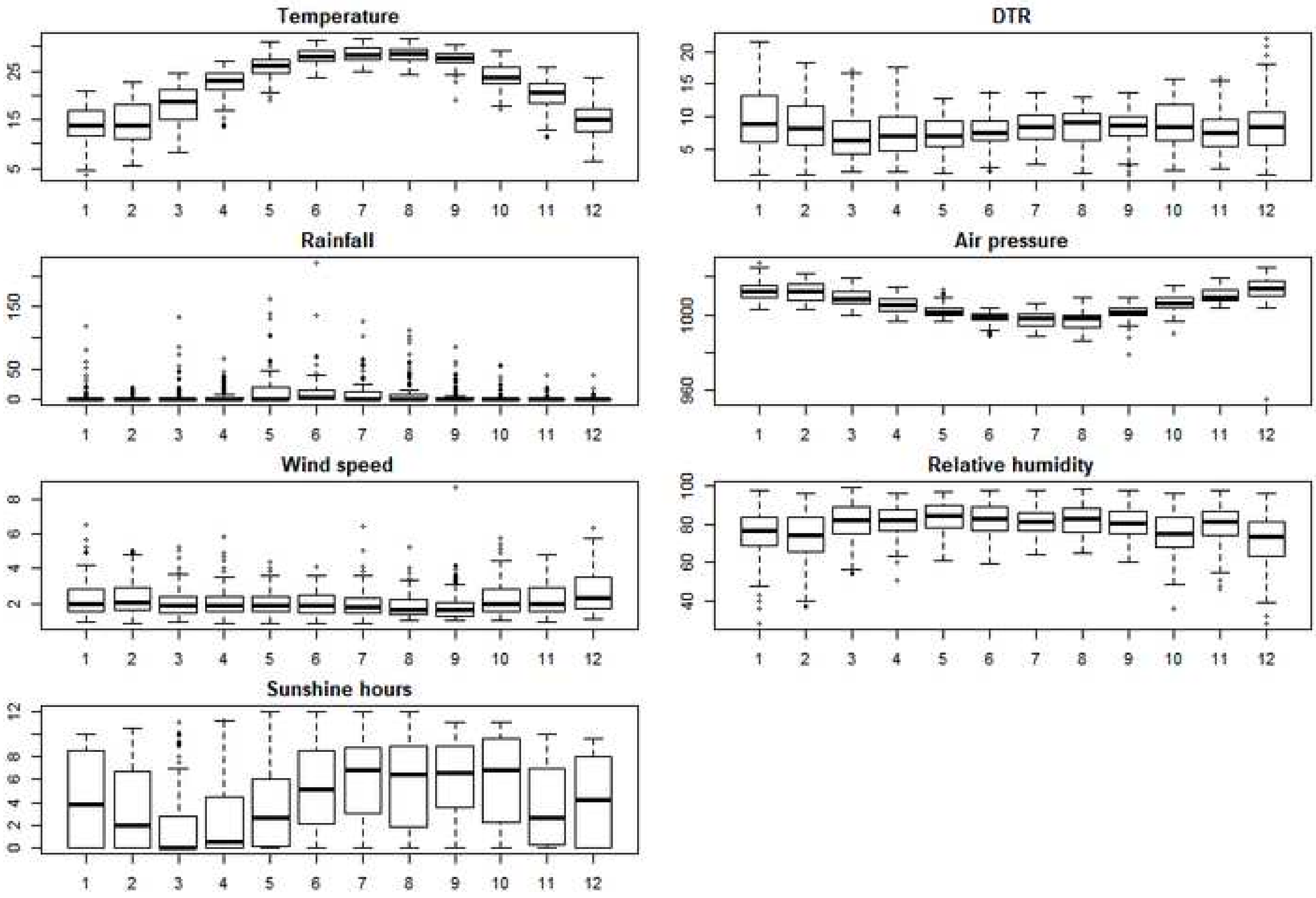

Click here to access/download;Figure;Fig 5.tif

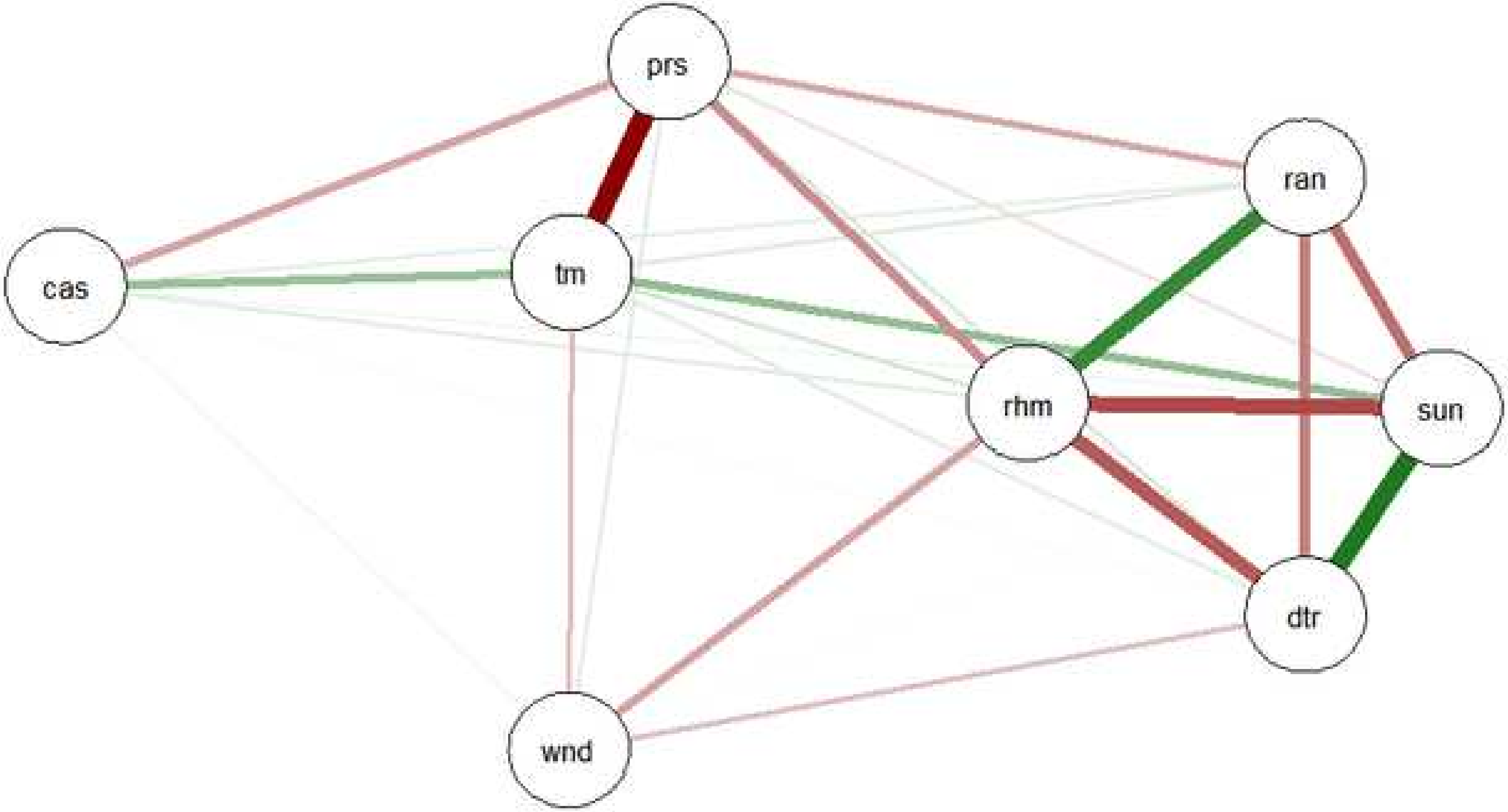

Fig 6

Click here to access/download;Figure;Fig 6.tif

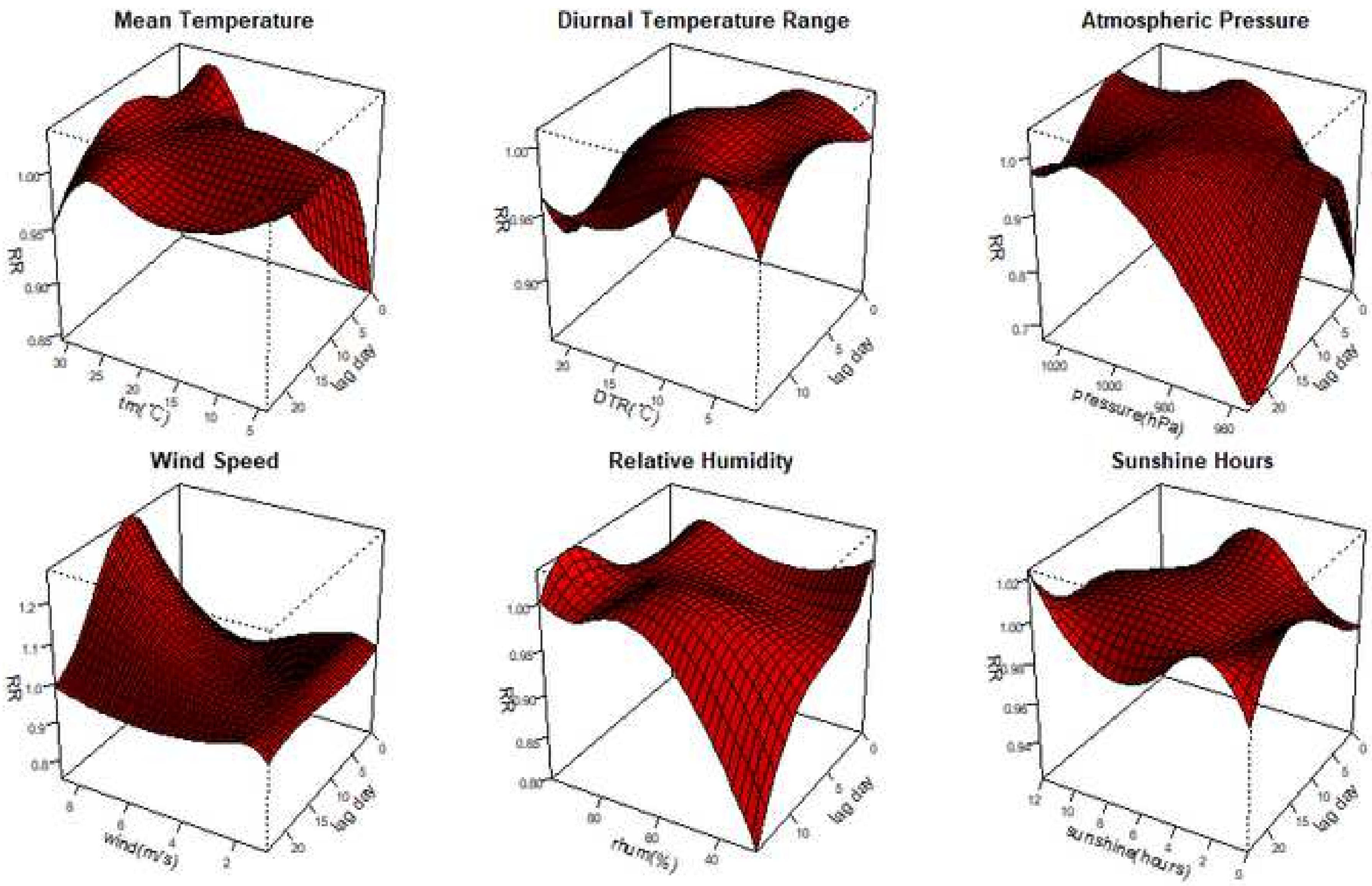

Fig 7

Click here to access/download;Figure;Fig 7.tif

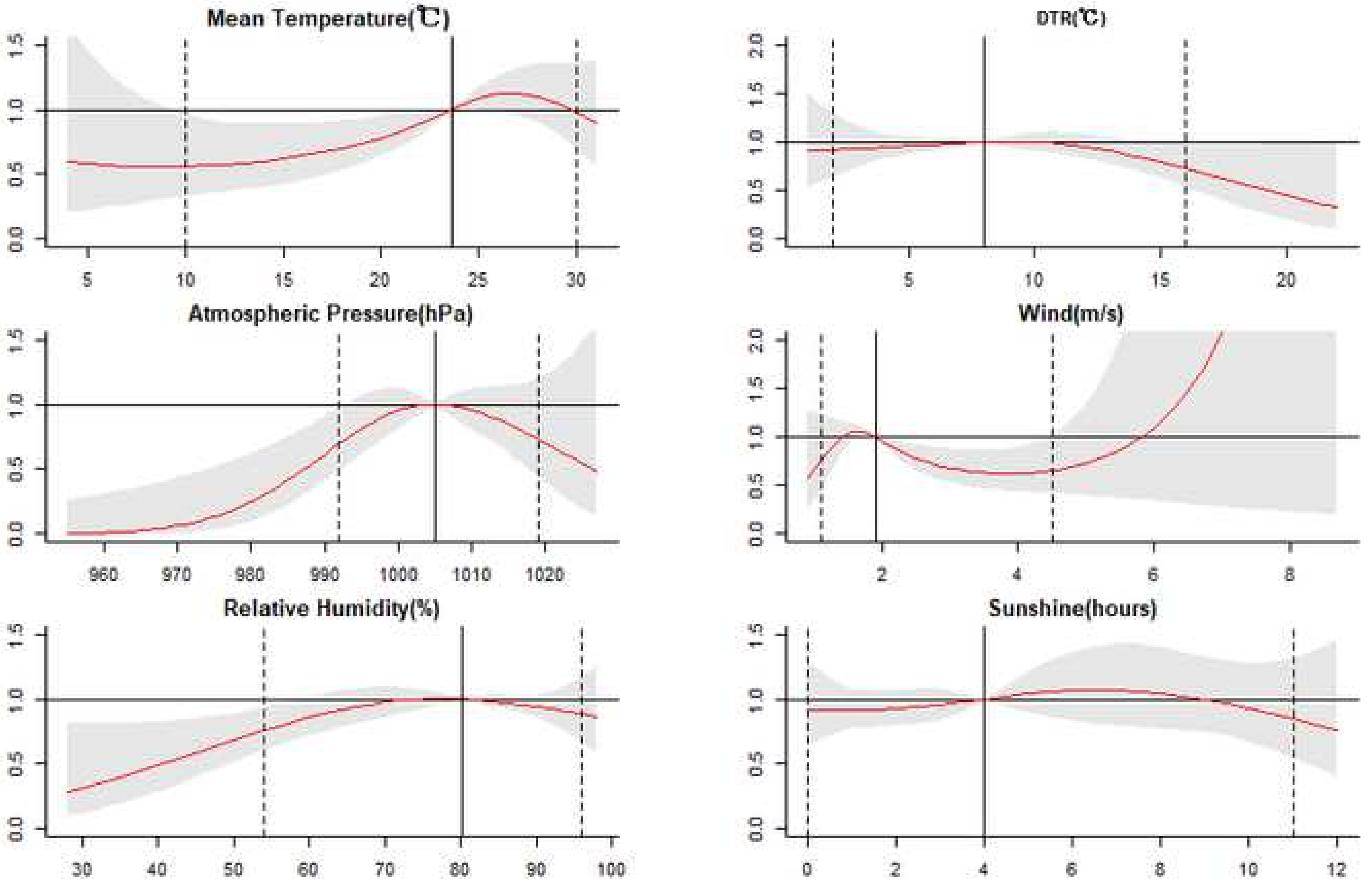

Fig 8

Click here to access/download;Figure;Fig 8.tif

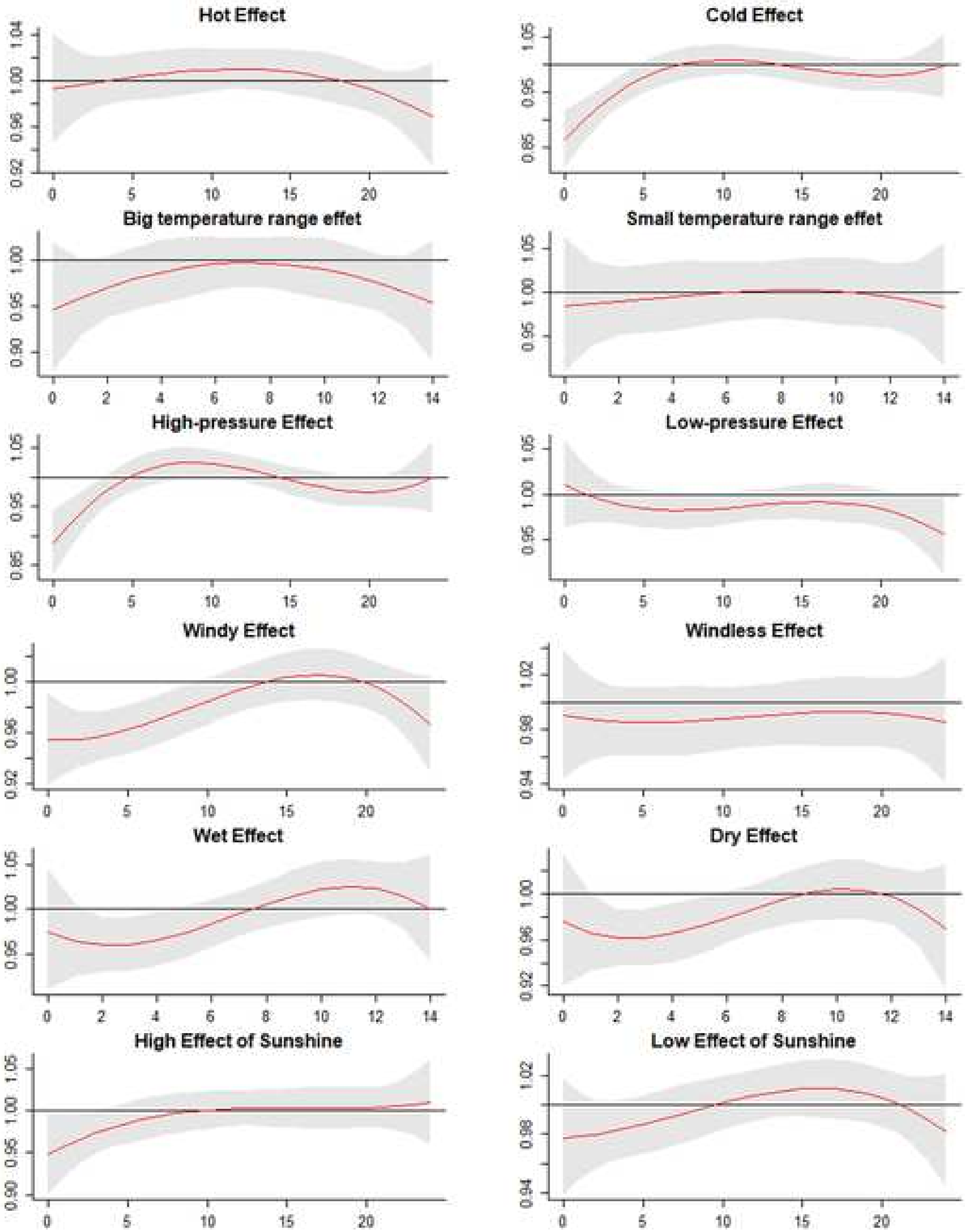

Click here to access/download
Supporting Information - Compressed/ZIP File Archive
mumps data and r project.zip

~~The roles~~~~Effects~~The role of meteorological factors on mumps incidence among children in Guangzhou, Southern China

Jian-yun Lu[a#], Zhi-cong Yang[b#], Meng-meng Ma[a], Zhou-bin Zhang[c*]

[a]Department of Infectious Disease Control and Prevention, Guangzhou Center for Disease Control and Prevention, Baiyun District Qi De Road in Guangzhou, Guangdong Province, China

[b] Director, Guangzhou Center for Disease Control and Prevention, Baiyun District Qi De Road in Guangzhou, Guangdong Province, China

[c] Vice Director, Guangzhou Center for Disease Control and Prevention, Baiyun District Qi De Road in Guangzhou, Guangdong Province, China

[*]Corresponding author:

Email: zhoubinzhanggz@21cn.com

Their contributions were the same and could be acted as co-first author

Formatted: Left: 1", Right: 1", Section start: Continuous, Numbering: Continuous

Commented [A1]: Dear author,
Thank you very much for the privilege of reading through and editing this manuscript. In addition to editing for language, grammar, and conciseness, all the formatting requirements of PLOS One have been applied. The entire document has also been spell-checked. Please go through all my changes and comments carefully, and do not hesitate to contact me should your intended meaning not be satisfactorily conveyed, or if you require any clarifications; I will be happy to answer any questions you might have. I wish you all the very best with the submission.

Thank you.

Formatted: Font: 14 pt

Formatted: Font: 14 pt

Formatted: Font: Bold

Formatted: Font: 14 pt

Formatted: Font: 14 pt

Formatted: Font: Bold

Formatted: Font: 14 pt

Formatted: Font: 14 pt

Formatted: Font: 14 pt

Formatted: Font: 14 pt

Formatted: Font: 14 pt

Formatted: Font: 14 pt

Formatted: Font: 14 pt

Formatted: Font: Bold

Formatted: Font: 14 pt, Not Bold

Formatted: Centered, Line spacing: Multiple 1.15 li

*Corresponding author at: Guangzhou Center for Disease Control and Prevention, Baiyun District Qi De Road in Guangzhou, Guangdong Province, 510440, China.
Email:zhoubinzhanggz@21cn.com

Abstract

Mumps, a common childhood disease, ~~had~~ has a high incidence in Guangzhou city, China. It ~~was~~ has been proven that ~~the~~ mumps ~~was~~ is influenced by seasonality. However, the role of meteorological factors among children ~~was~~ is yet to be fully ascertained. This study ~~explores~~ explored the association between meteorological factors and the incidence of mumps among children in Guangzhou. ~~The d~~Distributed lag nonlinear models ~~(DLNMs)~~ were used to evaluate the correlation between meteorological factors and the incidence of mumps ~~in~~ among children from 2014~~-~~–2018. The nonlinear lag effects of some meteorological factors were detected. Mean temperature, atmospheric pressure, and relative humidity were positively correlated with mumps incidence, ~~which was opposite~~contrary to that of ~~the~~ wind speed. Extreme effects of temperature, wind speed, atmospheric pressure, and relative humidity on the incidence of mumps among children in Guangzhou were evaluated ~~by a~~in a subgroup analysis according to ~~of~~ gender and age. Our preliminary results offered fundamental information to ~~get a full understanding about~~better understand the epidemic trends of mumps among ~~in~~ children ~~in order~~ to develop an early warning system, and strengthen the intervention and prevention of mumps.

Keywords: ~~Distributed~~ distributed ~~Lag~~ lag ~~Nonlinear~~ nonlinear ~~Models~~models, ~~Climatic~~ climatic ~~Factors~~factors, ~~Effect~~effect

Introduction

Mumps is a common childhood disease caused by the mumps virus ~~focused on the~~that mostly affects children aged 5~~-~~–9 years~~ old, which was caused by the mumps virus~~(1, 2). ~~The clinical feature was~~It manifests clinically as an ~~inflammatory~~ inflammation of the

parotid gland with precursory fever. Most ~~of the patients~~cases are mild and self-limiting, but ~~it could~~can ~~cause~~ result in some severe complications~~ of infections,~~ such as deafness, aseptic meningitis, and encephalitis (3). The incubation period of mumps is 12~~ to ~~–24 days, with a median ~~as~~ of 19 days (4). Mumps is not only transmitted by droplet spread, but also ~~by~~ via direct contact or contaminated fomites (1).

Mumps can be effectively prevented by ~~vaccine~~vaccination~~,~~. ~~a~~ A sharp reduction in incidence was observed after ~~the~~ routine vaccination (5)~~;~~; however, many countries, including France (6), Ireland (7)~~,~~, UK (8), and USA (9) ~~had~~ have experienced resurgences of mumps and reported large scale outbreaks over the past decade. Since 2007, the mumps vaccine has been integrated into the National Immunization Program in China, and the measles~~-~~, mumps~~-~~, and rubella (MMR) or measles and ~~-~~mumps (MM) vaccine has been provided ~~for~~ free of charge to children aged 18~~–~~–24 months in Guangzhou since 2008 (10). However, the incidence rates of mumps in China rose to 33.9/100,000 and 35.6/ 100,000 during 2011~~–~~–2012 ~~(11),~~ (11); ~~meanwhile~~meanwhile, 10008 and 7856 mumps cases were reported in Guangzhou (10). A ~~O~~one dose vaccine program is ~~not~~ unavailable to fully control the epidemic of mumps, ~~even if~~although the immunization coverage rate~~s~~ is about 80% to 90% nationwide ~~(12),~~ (12). ~~the~~ The two doses vaccination program was recommended by the ~~WHO~~World Health Organization (13) and the polyvalent mumps vaccine was recommended in the USA (9). Hence, mumps is still a serious ~~worldwide~~ public health ~~disease~~ concern ~~for~~ among children not only in Guangzhou, China, but also ~~in the global~~worldwide.

The incidence of mumps ~~revealed~~ showed apparent seasonality in various areas, with the peak in late spring, early summer, and winter. Different seasonal distributions ~~was~~ were even observed in southern and northern China (11, 12). ~~It~~ This hinted that mumps incidence was influenced by seasonal undulation. Therefore, we ~~indicated~~ hypothesized that meteorological factors might play a vital role in the spread of mumps. Recently, the effects of

Formatted

Formatted

Formatted: First line: 0 ch

Formatted

Formatted

Commented [A2]: The original sentence was rather wordy and has been split.

Formatted

Formatted

Formatted

meteorological ~~effects~~ factors on some infectious diseases have been explored extensively ~~explored~~ as early warning signals of possible epidemics, such as those of hand~~-~~, foot~~-~~, and mouth disease (14), varicella (15), and influenza (16). Limited studies ~~had~~ have been ~~done~~ conducted to explore the association between meteorological factors and the incidence of mumps. A study using the ARIMAX model in Beijing, China showed that ~~rising~~ increasing temperature would cause ~~the~~ an increase in the incidence of ~~of~~ mumps ~~incidence~~(17), whereas a study ~~using~~ that used a generalized additive model in Jining ~~exhibited~~ showed that an increasing trend in the incidence of mumps was observed when the temperature rose above 4~~-~~ºC (18). However, a study which applied ~~pearson~~ Pearson's correlation analysis in Czech Republic showed that there was no relationship between ~~the~~ mumps ~~cases had no relationship~~incidence and ~~with~~ drier and warmer weather (19). Besides, a ~~poison~~ Poisson regression model was ~~used~~ applied in a ~~research~~ study on mumps in 10 cities in Guangxi Province (20). However, most of the previous studies only focused on linear effects but not lag effects~~.~~ . A study conducted in Shenzhen city applied the distributed lag nonlinear model (~~dlnm~~DLNM) ~~to explored~~in exploring the relationship between wind velocity and mumps cases ~~(21),~~ (21). ~~studies~~ Studies conducted in Fujian Province~~,~~ ~~and~~ and Guangzhou city applied the ~~dlnm~~ DLNM ~~to explore~~in exploring the nonlinear correlations between meteorological factors and the incidence of mumps (22, 23). However, the nonlinear and lag effects of climatic factors on the transmission among children, who ~~were~~ are the main population ~~of~~ affected by mumps, are still unclear at present. ~~Although a~~A study conducted in Japan explored the linear relationship between meteorological factors and mumps incidence among children ~~with the~~via negative binomial regression analysis (24). What ~~were~~ are the roles of meteorological factors on mumps incidence among children ~~on mumps~~? ~~It is urgent~~There is an urgent need to explore these nonlinear relationships ~~with~~ using lag effects~~.~~.

In this study, an ecological methodology was used to analyze the features of mumps in Guangzhou from 2014 to 2018. We built ~~the distributed lag nonlinear model~~DLNMs (25), which can flexibly depict the nonlinear correlation and describe delayed effects in time-series ~~study~~studies, to analyze the effects of meteorological factors on mumps transmission ~~in~~ among children. The findings of our study may be helpful ~~to~~ in developing an early warning system for mumps epidemics ~~in~~ among children, improvinge ~~the~~ preventative ~~measurement~~ measures, and ~~reduce~~ reducing the risk of mumps infection ~~for~~ among children.

Materials and methods

Study region

Guangzhou~~,~~ is the third largest city in China with a total of 8.97 million registered inhabitants. It ~~has~~ is a typical meteorologically humid subtropical ~~feature of~~area ~~humid subtropical,~~ ~~which~~ and is locatesd ~~at~~ within 112°~~57'E~~ 57′E to 114°3′~~'~~E and ~~at~~ 22°26′~~'~~N to 23°56′~~'~~N (**Fig 1**). In Guangzhou, the summer season is wet and hot, whereas the winter season is dry and cool.

Fig 1. The geographical location of study area in Guangzhou city, Southern China.

Data sources

In this ~~research~~study, ~~the~~ clinically ~~diagnosis~~ diagnosed cases of mumps ~~under~~ among persons younger than 18 years ~~old~~ were obtained from the National Notifiable Disease Report System from January~~,~~ 2014 to Dec~~,~~ember 2018. ~~The clinical feature of~~Clinically, mumps ~~is~~ was defined as an acute onset of bilateral or unilateral tender, ~~with~~ self-limited swelling of the parotid or other salivary glands ~~sustaining~~ lasting for more than 2 days. Mumps ~~was the~~is a notifiable disease in Guangzhou~~,~~; consequently, all clinicians are

Formatted ...
Formatted: Space Before: 12 pt
Formatted
Formatted ...
Formatted: First line: 0 ch
Formatted ...
Formatted: Font: 12 pt
Formatted: Indent: First line: 1 ch, Space Before: 12 pt
Formatted ...
Formatted: Font: Bold
Formatted: Space Before: 12 pt
Formatted ...
Formatted: First line: 0 ch
Formatted ...

required to compulsorily notification is required to report cases to local health authorities within 24 hours through via the National Notifiable Disease Surveillance System for all clinicians.

The dDaily meteorological data, including mean temperature (°C), diurnal temperature range (°C) (it was equal to the highest temperature minus the lowest temperature), atmospheric pressure (HPahPa), aggregate rainfall intensity (mm), wind speed (m/s), relative humidity (%), and sunshine hours duration (hours) were collected simultaneously from the Guangzhou Meteorological Bureau.

Statistical analysis

We used performed a descriptive analysis for of meteorological variables and the occurrence of mumps in Guangzhou. According to theBased on scatter plots, we got a conclusionconcluded that the correlations between the meteorological factors and mumps incidence were were nonlinear. The Mann-–Kendall and Pettitt tests were applied to initially analyze the tendency of the meteorological variables and mumps cases among children. Most of the variables have a risingtended to increase or decreasing tendencye. For Due to the reasons mentioned above, the Distributed Lag Nonlinear Models (DLNMs), were applied to detect the relationships between meteorological variables and the occurrence of mumps. Atmospheric pressure had awas strongly correlation correlated with average temperature (r=-0.82, P<0.01) by the result ofas per the Spearman's correlation analysis;. hence Hence, the two variables was were not put together intocombined in a model to avoid the potential problem of collinearity problem. A Poisson regression, constructed performed with the quasi-Poisson function, was established to analyzeused to assess the over-dispersion in theof daily cases of mumps in the DLNMs. The calculation is shown as belowperformed using the following equation:

Formatted

Formatted

Formatted: Font: Bold

Formatted: Space Before: 12 pt

Formatted: Font: Bold

Formatted

Formatted

Formatted

Formatted

$$~~L~~\log[E(Y_t)] = \alpha + \sum NS_i\,(X_i, df_i) + \delta Dow + \delta Holiday_t + NS(Time, 7\ per\ year),$$

~~W~~where Y_t ~~stands for~~represents the number of ~~daily~~ cases of mumps reported ~~in~~ on day t; both α and δ are the intercepts; NS represents natural cubic spline; X ~~stands for~~represents meteorological variable~~s~~s, such as temperature, diurnal temperature range, rainfall, atmospheric pressure, wind speed, relative humidity, and sunshine hours; df ~~means~~ is the degrees of freedom; Holiday is defined as a binary variable~~,~~ (if day t is a public holiday or school vacation, it is marked as 1, otherwise it ~~equals~~ is marked as 0); Dow represents the day of week effect; and Time denotes long term trends and seasonality. We selected the df of day to control long term trends, seasonality, and meteorological variables ~~by~~ using the Akaike information criterion (AIC). Based on the AICs, we cho~~o~~se df=3 for NS ~~to~~ according to the meteorological variables mentioned above. After ~~we combined with the~~combining the incubation period of mumps, ~~the~~ df=24 was chosen ~~to~~ for mean temperature, atmospheric pressure, and wind speed, and~~the~~ df=14 was chosen ~~to~~ for relative humidity.

~~The~~ Extreme high effects ~~effect~~ were defined as when the value of meteorological variables were beyond the 97.5th percentile~~; was defined as an extreme high effect,~~ while when the value was below the 2.5th percentile, it was defined as an extreme low effect.

All statistical tests were two-sided~~,~~. ~~if the~~Analysis items with P~~-value~~ <0.05 ~~, it had~~were considered statistically ~~significance~~significant. All statistical analyses were performed using R ~~software (~~version 3.5.3 (The R Development Core Team, Vienna, Austria) ~~was applied to all analyses,~~ and the DLNMs were built ~~by~~ using the *dlnm* package ~~named~~in the R environment~~ dlnm~~.

We ~~conducted~~ performed the sensitivity analyses by shifting the df (5–9) per year ~~for time~~ to control for seasonality and long-term trends using time, and changing the df (4–7) for mean temperature, atmospheric pressure, diurnal temperature range, wind speed, accumulated

Formatted: Font: 12 pt

Formatted: Centered

Formatted

Formatted

Formatted: First line: 0 ch

Formatted

Formatted

Formatted

rainfall, relative humidity, and sunshine hours.

Ethical permissionconsiderations

This The work study protocol was granted approved by the Ethics Committee of the Guangzhou Center for Disease Control and Prevention. All data were fully anonymized before we accessed them.

Results

From January 1st, 2014 to December 31st, 2018, a total amount of 9842 mumps cases among persons younger thanunder 18 years old were reported in Guangzhou. During 2014 to 2018, the breakdown of description of mumps cases was 1962, 2077, 1838, 1977, and 1988, respectively. The gender ratio of gender was 1.83 (6362 males to 3480 females). Among the groups aged 0–3 years, 4–6 years, 7–14 years, and 15–17 years, there cases were 2197 (22.32%), 3773 (38.34%), 3672 (37.31%), and 200 (2.03%) cases, respectively. The peak of mumps occurrence in the study period occurred from May to July, with a total of 3087 cases (31.37%) **(Fig 2)**.

Fig 2. The boxplot of mumps cases among children in Guangzhou, 2014–2018.

The A description of meteorological variables and mumps cases were shown as belowis given as follows **(Fig 3)**. The mean daily mean temperature was 22.24ºC, and the means of diurnal temperature range, aggregate rainfall, atmospheric pressure, wind speed, relative humidity, and hours of sunshine were 8.20ºC, 6.04 mm, 1005.41 hPa, 2.16 m/s, 79.19%, and 4.42 hours, respectively. The monthly description distribution was is shown in the monthly boxplot of meteorological factors **(Fig 34)**. The results of the Mann–Kendall and Pettitt tests were are shown in Table 1. Except for the cases, temperature and sunshine hours, most of the variables

tended to increase or decrease~~have a rising or decreasing tendency~~.

Fig 3. The boxplot of meteorological factors and mumps cases among children in Guangzhou, 2014–2018.

Fig ~~3~~4. The monthly boxplot of meteorological factors ~~-~~in Guangzhou, 2014–2018.

Table 1. ~~the~~ The results of the Man-Kendall and Pettitt tests for the meteorological variables and mumps cases among children in Guangzhou, 2014-2018

variables	P	S	change-point number	change-point	P
case	0.3	+	1542	2018/3/22	**<0.01**
temperature	0.19	+	129	2014/5/9	**<0.01**
DTR	**<0.01**	-	414	2015/2/18	**<0.01**
rain	**<0.01**	-	986	2016/9/12	**<0.01**
pressure	**<0.01**	-	482	2015/4/27	**<0.01**
wind	**<0.01**	+	371	2015/1/6	**<0.01**
relative humidity	**<0.01**	+	794	2016/3/4	**<0.01**
Sunshine hours	0.88	-	674	2015/11/5	**0.02**

S: '+' means a rise tendency , '-'means a decrease tendency

~~The~~ **Fig** ~~4~~ 5 ~~provided~~ shows the correlation network between meteorological factors and mumps cases among children. ~~Analysis of~~ Spearman's correlation analyses~~s~~ revealed that the incidence of mumps was positively correlated with mean temperature, relative humidity, aggregate rainfall, and hours of sunshine. ~~To~~ On the contrary, wind speed~~,~~ and atmospheric pressure ~~showed a~~were negatively ~~correlation~~ correlated with the incidence of mumps. Mean temperature ~~had a~~was strongly ~~correlation~~ correlated ~~to~~ with atmospheric pressure (r=-0.82, P<0.01~~).~~).

Fig 5. The correlation network between meteorological factors and mumps cases among children in Guangzhou, 2014–2018.

*cas represents cases; tm represents temperature; prs represents atmospheric pressure;

Formatted

Formatted: Indent: First line: 0 ch, Space Before: 12 pt, After: 12 pt

Formatted: Font: 12 pt

Formatted: Font: 12 pt

Formatted: Space Before: 12 pt, After: 12 pt

Formatted

Formatted

Formatted: Space Before: 12 pt, After: 12 pt

Formatted

Formatted

Formatted: Indent: First line: 3 ch, Space Before: 12 pt, After: 12 pt, Tab stops: 0.01", Left + Not at 0.59"

Formatted

wnd represents wind; dtr represents diurnal temperature range; ran represents rain; rhm represents relative humidity; sun represents sunshine hours. The green line means positive relationship, whereas the red one means negative relationship. The width of the lines dedicates the degree of *r*. The thicker the line, the greater the correlation.~~**Fig 4.** The correlation network between meteorological factors and mumps cases among children in Guangzhou, 2014–2018.~~

~~The~~ **Fig** ~~5~~ 6 ~~illustrated~~ illustrates the correlation between mumps cases and meteorological variables with different lag ~~days~~ periods in Guangzhou. Specific features ~~was~~ were observed in various variables with a nonlinear curve. The highest relative risk (RR) was 1.005 (95% confidence interval [CI]: 1.001–1.010) at lag day 21, when the daily mean temperature was 25.0ºC. When atmospheric pressure was 1027 hPa at lag day 0, the lowest RR was 0.775 (95% CI: 0.655–0.916). ~~For~~ When relative humidity~~, when it~~ was 72.0%, the highest RR was 1.014 (95% CI: 1.003–1.026) at lag day 12. ~~For~~ When wind velocity~~, when it~~ was 8.70 m/s, the highest RR was 1.269 (95% CI: 1.033–1.560) at lag day 11.

Fig ~~5~~6. Plots of the relative risk of climatic factors on the incidence of mumps in children, including mean temperature, diurnal temperature range, atmospheric pressure, wind speed, relative humidity and sunshine hours.

For a better explanation, the one-dimensional curves were plotted about the overall effects of meteorological factors over the corresponding lag days (**Fig** ~~6~~7). In a word, mean temperature,

Formatted: Space Before: 12 pt, After: 12 pt

Formatted ...

Formatted: Font: 12 pt

Formatted: Indent: First line: 0 ch, Space Before: 12 pt, After: 12 pt

Formatted: Font: 12 pt

Formatted: Space Before: 12 pt

Formatted ...

Formatted ...

Formatted ...

Formatted ...

Formatted: Space Before: 12 pt, After: 12 pt

Formatted ...

atmospheric pressure, and relative humidity ~~was~~ were associated with mumps ~~cases~~incidence, ~~which was on the contrary to the~~unlike wind speed. The ~~relative risk (~~RR~~)~~ of mean temperature significantly increased from 10 ºC to 23.6 ºC, whereas ~~the RR~~that of wind speed significantly declined from 1.9 ~~m/s~~ to 4.5 m/s. Atmospheric pressure and relative humidity both showed a reverse U-shaped curve relationship with mumps. However, the RRs just had a significantly ~~rising~~ increasing trend before 992 ~~HPa~~ hPa and 60%, respectively. There ~~was~~ were no significant relationships between other meteorological variables ~~in our study~~ and the incidence of mumps in our study~~—~~.

Formatted

Fig ~~6~~7. The estimated overall effects of mean temperature, diurnal temperature range, atmospheric pressure, wind speed, relative humidity, and sunshine hours along corresponding lag days. The Y lab represents the value of relative risk, the X lab represents the value of relevant variables. The red lines represent mean relative risks and the grey regions represent 95% CIs.The black vertical line represents the medians of the climatic factors, and the dotted lines represent the 2.5 percentile and the 97.5 percentile for the meteorological factors, respectively.

Formatted

The cumulative extreme effects of meteorological factors on mumps along the corresponding lag days ~~were drawn~~are shown in **Fig** ~~7~~8. ~~The~~ As shown in **Table 2**, ~~showed that~~ the extreme low effect of temperature was significant and the lowest RR was 0.88 (95% CI: 0.83–0.93) at lag day 0, whereas the extreme high effect of temperature was not significant. On the contrary, the extreme high effects of atmospheric pressure and wind speed were statistically significant, ~~but~~ whereas the extreme low effects were not. For atmospheric pressure, the lowest RR was 0.88 (95% CI: 0.82–0.94) at lag day 0. For wind speed, the lowest RR was 0.94 (95% CI: 0.89–0.98) at lag day 0. Regarding relative humidity, the RR of the extreme~~ly~~ high effect was 0.96 (95% CI: 0.94–0.99) at lag day 2, and the RR of the

Formatted

Formatted: Indent: First line: 0 ch, Space Before: 12 pt, After: 12 pt, Tab stops: Not at 0.59"

extreme~~ly~~ low effect was 0.96 (95% CI: 0.93–0.99) at lag day 2. For the extreme~~ly~~ high and low effects of sunshine hours, the RRs ~~was~~ were 1.03 (95% CI: 1.02–1.04) at lag day 2 and 1.02 (95% CI: 1.01–1.03) at lag day 17, respectively.

Fig ~~7~~8. The extreme effects of mean temperature, diurnal temperature range, atmospheric pressure, wind speed, relative humidity, and sunshine hours with extreme high effects (97.5%) and extreme low effects (2.5%).The Y lab represents the value of relative risk, the X lab represents the value of lag day. The red lines represent mean relative risks and grey regions represent 95%CIs.

Table 2

The extreme effects of meteorological variables on the incidence of mumps among children along the lag days in Guangzhou, 2014–2018.

Meteorological variables	Lag day	High effect (97.5%)		Lag day	Low effect (2.5%)	
		RR	95%CI		RR	95%CI
Mean temperature	/	/	/	0	0.88	0.83–0.93
Atmospheric pressure	0	088	0.82-0.94	/	/	/
Wind speed	0	0.94	0.89-0.98	/	/	/
Relatively humidity	2	0.96	0.94-0.99	2	0.96	0.93–0.99

In the analyses by subpopulation, the extreme low effects of temperature, atmospheric pressure, and relative humidity, and the extreme high effect of wind speed ~~showed~~ were statisticall~~y~~ ~~significance~~ significant in the female population, but no significant extreme effect

Formatted: ...
Formatted: Font: 12 pt
Formatted: ...
Formatted: Indent: First line: 0 ch
Formatted: ...
Formatted
Formatted
Formatted
Formatted
Formatted: Font: 12 pt
Formatted: Indent: First line: 0 ch, Space Before: 12 pt
Formatted: ...

was observed in the male population. The extreme low effect of temperature was observed ~~between~~ in the 4–6 year olds group (RR=0.25, 95% CI: 0.11–0.60) and 15–17 ~~years~~ year old group (RR=0.01, 95% CI: 0.00–0.57). The extreme high effect of atmospheric pressure was ~~revealed~~ observed in ~~at~~ the 4–6 year~~s~~ age group (RR=0.46, 95% CI: 0.21–0.98) **(Table 3).**

Sensitivity analyses indicated ~~alike~~ similar results after the changes in the df for long-term trends and seasonality. Altering the dfs for temperature, atmospheric pressure, wind speed, and relative humidity, the original models ~~revealed~~ showed a better robustness ~~than alternative ones~~ compared to their alternatives.

Table 3

The cumulative extreme effects of meteorological variables on mumps cases of children by sex and age.

**Variables**	**Mean temperature**				**Wind speed**				**Relative humidity**				**Atmospheric pressure**			
	**low effect**		**high effect**		**low effect**		**high effect**		**low effect**		**high effect**		**low effect**		**high effect**	
	RR	95% CI	RR	95% CI	RR	95% CI	RR	95% CI	RR	95% CI	RR	95% CI	RR	95% CI	RR	95% CI
total	**0.56**	**0.32-0.99**	0.99	0.70-1.40	0.77	0.47-1.28	0.64	0.40-1.01	**0.76**	**0.61-0.94**	0.89	0.67-1.17	**0.70**	**0.49-0.99**	0.75	0.45-1.24
male	0.88	0.44-1.73	0.91	0.59-1.39	0.76	0.41-1.41	0.88	0.50-1.56	0.95	0.73-1.23	1.06	0.75-1.49	0.87	0.56-1.34	0.81	0.43-1.50
female	**0.23**	**0.10-0.62**	1.12	0.66-2.02	0.77	0.74-1.71	**0.34**	**0.16-0.72**	**0.50**	**0.35-0.72**	0.64	0.41-1.00	**0.47**	**0.26-0.83**	0.64	0.28-1.45
< 4years	1.83	0.60-5.65	1.40	0.69-2.83	0.99	0.36-2.71	0.74	0.29-1.87	0.73	0.48-1.11	0.70	0.39-1.24	0.54	0.26-1.09	0.97	0.37-2.55
4-6 years	**0.25**	**0.11-0.60**	1.01	0.58-1.75	0.68	0.31-1.49	0.51	0.25-1.05	0.77	0.56-1.07	1.28	0.83-1.96	0.81	0.46-1.42	**0.46**	**0.21-0.98**
7-13 years	0.82	0.32-2.10	0.83	0.48-1.44	0.79	0.35-1.80	0.81	0.37-1.76	0.76	0.52-1.11	0.72	0.45-1.14	0.73	0.41-1.30	1.25	0.51-3.05
14-17 years	**0.01**	**0.00-0.57**	0.18	0.01-2.74	0.35	0.01-16.28	0.05	0.00-2.18	0.48	0.07-3.29	0.38	0.05-3.09	0.20	0.01-2.66	0.08	0.00-3.08

Discussion

In ~~the~~ this study, the incidence of mumps ~~cases of~~among children in Guangzhou fluctuated seasonally with the epidemic peak from May to July. The trough of mumps ~~cases in~~incidence among children occurred in February, coinciding with the winter holiday in school. However, our result ~~was different from~~differed from that of ~~the~~ a study conducted in Ireland, which ~~dedicated~~ detected a relatively low mumps ~~cases~~ incidence in summer and a peak~~ed~~ in autumn (7), and a study conducted in the Netherlands with two seasonality peaks in spring and autumn (26). In China, the epidemic pattern in Guanghzou ~~was also different from~~also differed from that of Beijing city, Zibo city in Shandong province, and Chongqing city, which showed ~~that~~ two epidemic peaks from April to July and October to January, with an emphasis on the former (17, 27, 28). It indicated that the effect of meteorological factors on the occurrence of mumps may be distinct in various regions with particular~~ly~~ climatic features.

Climatic variables such as temperature, relative humidity, and wind speed have been proven to play a key role in the occurrence of certain infectious diseases (29-31). Recently, ~~it drew~~ more and more attention has been drawn to the effects of meteorological factors on mumps incidence. ~~Previously, s~~Some studies have been ~~done~~ conducted to explore the relationships between meteorological factors and mumps cases;~~,~~ however, most of the ~~studies~~ studies just detected ~~the~~ linear relationships without lag effects. Recently, ~~the~~ DLNMs ~~were~~ have been applied widely ~~applied~~ to evaluate these nonlinear correlations with lag effects (10, 21, 23). However, these relationships among children ~~are~~ still need to be explored.

~~As far as we know~~To the best of our knowledge, ~~it~~ this is the first ~~research~~ study to apply DLNMs to detect the effects of meteorological variables on ~~the~~ mumps ~~cases~~ incidence among~~of~~ children in a subtropical city in China. ~~The n~~Nonlinear relationships were observed in some meteorological variables in our study. When the mean temperature ranged from 10–~~ºC to~~ 23.6~~-~~ºC, the RR showed an increased trend. Our finding was not fully consistent ~~that of~~

Formatted

Formatted: First line: 0 ch

Formatted

Formatted

with previous studies. A study conducted in Mexico ~~dedicated~~ showed ~~that~~ that the mumps ~~cases got~~ aincidence declined markedly ~~decline~~ during the summer (32). However, ~~the~~ a study conducted in Taiwan (33) ~~showed~~ reported that the incidence of –mumps ~~cases~~ ~~began~~started to increase at ~~the~~ a temperature of 20° °C, and started to decline after 25° °C, whereas a study conducted in Jining, China (18) and a study focused on children in Japan (24) both reported linearly positive associations between mumps ~~cases~~ incidence and temperature. A previous study conducted in Guangzhou (22) also revealed a positive correlation between mean temperature and the ~~overall population~~ incidence of mumps in the overall population. However, another study conducted in Jining showed a U-shaped relationship curve between mean temperature and mumps incidence (34). The discrepancy may be due to the differen~~t~~ces in methodolog~~ies~~y, geographic location, and climatic characteristics. The incidence of mumps depended on the contact rate, which might be influenced by social behavior (35). A previous epidemiologic~~y~~ study conducted in Canada showed that adolescents usually ~~join~~ engage in more physical ~~activities~~ activity in the warmer months than in the colder months (36). Children are more likely to come into ~~could~~ contact with each other in warmer weather, which might result in ~~the~~ an increase ~~of~~ in the incidence of mumps ~~cases~~. When the temperature continued to ~~rise~~increase, ~~the children might decrease the~~ outdoor activities among children might decrease, ~~and~~ reduc~~e~~ing the risk of mumps infection~~,~~; ~~but~~ however, the extreme high effect was not statistically significant. For the same reason, the risk of mumps infection will be reduced in ~~the~~ extreme cold conditions~~,~~; however, the accumulated extreme cold effect was only observed ~~in~~ significantly among females ~~significantly~~. These might explain ~~be the explanation for~~ our finding ~~in~~ among children in Guangzhou and ~~it~~ ~~hinted~~ shows that preventative measures and health education should be strengthened in the temperature range mentioned above in our study.

~~Some studies'~~The results of some studies were inconsistent with ours. For example,

Formatted

Formatted: Indent: First line: 0 ch

Formatted

~~the~~ relative humidity was ~~dedicated~~ found to have a negative impact on mumps ~~cases~~ incidence with a threshold of 54% in Jining, China ~~(18),~~ (18). ~~but~~ However, another study conducted in Jining showed that the effect of relative humidity on mumps increased slightly and then decreased quickly, with a threshold of 64% ~~(34),~~ (34). ~~besides~~ Besides, a study conducted in Taiwan (33) ~~pointed out that~~found no correlation ~~was found~~ between relative humidity and mumps ~~cases~~incidence. Our ~~finding~~ study showed a U-shaped ~~curve~~ relationship between relative humidity and mumps incidence among children in Guangzhou, with a statistically significant~~ly~~ increase before a relative humidity ~~the~~ of 60% ~~of relative humidity~~. Meanwhile, ~~the~~ nonlinear extreme high ~~or~~ and low effects of relative humidity on mumps ~~cases~~ incidence were both found ~~in~~ among children. ~~Seldom r~~Studies on~~esearch was available to~~ the mechanism ~~about~~ underlying the effect of relative humidity on mumps incidence are rare~~, it~~ and should be ~~urged to~~ carr~~y~~ied out in the future. One possible ~~reason~~ mechanism might be as follows. Droplet spread is one of the modes of transmission ~~way~~ of the mumps virus (1). When the relative humidity ~~was~~ is low, ~~the~~ water in the droplets ~~evaporated~~ evaporates quickly. The longer the droplet with mumps viruses remains airborne, the easier it is the mumps infect the children (37). As the relative humidity increases, the effects ~~became~~ become insignificant~~,~~. ~~however~~However, an extremely high effect of relative humidity was observed with ~~the~~ an RR ~~as~~ of 0.96 (95% CI: 0.93~~–~~–0.99) at lag day 2, ~~it~~ which may be explained by the fact that high relative humidity often coincides with heavy rain, which will make children more ~~time~~ likely to stay at home (38) and reduce the contact rate. More studies should be carried out to ~~look for~~identify ~~the~~ biological evidence.

Wind could affect the suspension time of viruses and their diffusione distance (39, 40). Our ~~finding~~ study revealed that the RR decreased as the wind speed surpassed 1.9 m/s till 4.9 m/s. Moreover, the high effect of wind speed ~~revealed~~ yielded an RR ~~as~~ of 0.94 (95% CI: 0.89~~–~~–0.98). Our study result was similar to that of a study conducted in Shenzhen, which

Formatted
Formatted
Formatted

depicted a reverse ~~v~~V-shaped relationship between wind velocity and mumps incidence, with the peak at 2 m/s (21), and a study conducted in Fujian province (23), which showed a negative relationship between wind speed and mumps ~~cases~~incidence. In view of the resuspension phenomenon, the concentration of local viruses may be diluted by higher wind velocity (39, 41), which could ~~give a possible explanation for~~possibly explain our finding. In the subpopulation analysis, ~~the~~ an accumulated high effect of wind speed was observed ~~in the~~among females. ~~It~~ This may be explained by the reduction ~~of~~ in outdoor activity ~~for~~ among females in the extreme high wind velocity weather. ~~According to the~~Based on our findings, ~~the strengthen of~~ regular ventilation might be helpful ~~to~~ in protecting ~~the~~ children ~~to avoid infection of~~against infection with mumps.

In our study, ~~the~~ extreme low effects of mean temperature, atmospheric pressure, and relative humidity, and ~~the~~ an extreme high effect of wind speed were found ~~in~~ among females with ~~the~~ RRs values less than 1, whereas no extreme effect was detected ~~in~~ among males. ~~The reason may be due to the~~This may be due to factors such as physiological gender differences~~,~~ and the discrepancy in behavior patterns (42). For example, in ~~the~~ extreme weather conditions, ~~the~~ males ~~were~~ are more active and will be more likely to play with friends, which will increase the contact rate with children infected with mumps. ~~It~~ This ~~gave us a hint~~shows that children should cut down on going to crowded places such as playgrounds in the active period of mumps from May to July in Guangzhou.

Our results showed a positive correlation before 1000.05 ~~HPa~~hPa, after which point~~,~~ the correlation was not statistically significant. However, ~~the~~ previous studies showed different results. For instance, a study conducted in 10 cities in Guangxi province in China (20) ~~dedicated~~ reported that no ~~statistical~~ significant relationship was found between atmospheric pressure and the incidence of mumps, whereas a study conducted in Beijing (17) showed that atmospheric pressure ~~had a~~was negatively ~~association~~ associated with mumps

Formatted

Formatted

Formatted

~~cases~~incidence. Unfortunately, limited ~~study~~ studies are~~was~~ available ~~for~~ regarding the underlying mechanism. ~~An explanation may be as below~~This may be explained as follows. As the atmospheric pressure ~~increased~~ increases in the beginning, the cold air would enter and promote the spread of the virus, ~~which~~ leading to ~~a~~ high virus abundance in the atmosphere. After exceeding a certain value of atmospheric pressure, the effect mentioned above ~~was~~ is offset by high wind speed. However, further studies ~~should be done~~ should be conducted to explore the foundational mechanism in future.

Several limitations of our study should be acknowledged. At first, the data were only obtained in Guangzhou, southern China~~,~~; hence, it cannot stand for other cities or countries. Secondly, we used ~~the~~ meteorological data instead of the ~~individual~~ exposure conditions of individuals~~,~~; ~~so it~~this may lead to bias in our results. ~~What's more~~Further, ~~it was incapable focus to~~we could not ~~remove~~ exclude the possibility that some potential confounding factors existed in our research, including host susceptibility, preventive measures, geographic factors, and population density. Moreover, ~~the~~ data ~~of~~ regarding human serum ~~antibody~~ antibodies against~~of~~ mumps and information regarding ~~the~~ mumps vaccination ~~information was not~~were unavailable in our study. Finally, we did not ~~analyze the the~~assess factors regarding air pollution ~~factors~~ which ~~was~~ have been proven to affect the incidence of mumps (43). Therefore, more studies should be undertaken to take these variables into consideration.

~~5.~~ Conclusions

In general, compared to previous studies that focused on the effect of meteorological factors on mumps incidence, for the first time, we ~~firstly revealed the~~identified a significant nonlinear association between certain climatic variables and mumps ~~cases~~ incidence among children in Guangzhou with lag effects. At a certain corresponding range, mean temperature, atmospheric pressure, and relative humidity ~~was~~ were positively correlated with the incidence

Formatted

Formatted

Formatted: Font: 16 pt

Formatted: Space Before: 12 pt

Formatted: Indent: First line: 0 ch

Formatted: Font: 12 pt

Formatted

of mumps ~~in~~ among children, ~~which was~~ opposite to that of ~~the~~ wind speed. ~~Morever~~Moreover, extreme effects of the variables mentioned above ~~in~~ among mumps cases were detected ~~by~~ in a subgroup analysis according to ~~of~~ gender and age. These findings provide the underlying information to ~~get a~~ better understand~~ing of~~ the epidemic~~al~~ tendency of mumps. ~~According to~~Based on our results, some measures could be ~~done~~ implemented to minimize the occurrence of mumps. For example, preventative measures and health education should be strengthened in the temperature range ~~from~~ of 10–~~°C to~~23.6 °C, and ~~the strengthen of regular~~proper ventilation might be helpful ~~to~~ in protecting ~~the~~ children ~~to avoid~~against infection with~~of~~ mumps. ~~It also gave us a hint~~ We also implied that children should cut down on going to crowded places such as playgrounds in the active period of mumps from May to July in Guangzhou. ~~What we found~~Our findings in this ~~study~~ study will help ~~to~~ improve the early warning system~~,~~ and reinforce the prevention and intervention ~~for~~ of risk factors ~~of~~ for mumps among ~~children~~ children in Guangzhou.

Acknowledgments

We thank the Guangzhou Meteorological Bureau for offering climatic data for our study. We would like to thank Editage (www.editage.cn) for English language editing.

Supporting information files

The data and the R program of our study were uploaded as the supporting information file, which also included the data of subpopulation analyses.

References

1. Hviid A, Rubin S, Muhlemann K. Mumps. Lancet (London, England). 2008;371(9616):932-44.https://doi.org/10.1016/s0140-6736(08)60419-5.
2. Rubin S, Eckhaus M, Rennick LJ, Bamford CG, Duprex WP. Molecular biology, pathogenesis and pathology of mumps virus. The Journal of pathology. 2015;235(2):242-

Formatted

Formatted

Formatted

Formatted

Commented [A3]: As instructed, this list of references has been excluded during this round of editing.

Formatted: English (United States)

Formatted: English (United States), Check spelling and grammar

52.https://doi.org/10.1002/path.4445.

3. Grennan D. Mumps. Jama. 2019;322(10):1022.https://doi.org/10.1001/jama.2019.10982.

4. Richardson M, Elliman D, Maguire H, Simpson J, Nicoll A. Evidence base of incubation periods, periods of infectiousness and exclusion policies for the control of communicable diseases in schools and preschools. The Pediatric infectious disease journal. 2001;20(4):380-91.https://doi.org/10.1097/00006454-200104000-00004.

5. Galazka AM, Robertson SE, Kraigher A. Mumps and mumps vaccine: a global review. Bulletin of the World Health Organization. 1999;77(1):3-14

6. Vygen S, Fischer A, Meurice L, Mounchetrou Njoya I, Gregoris M, Ndiaye B, et al. Waning immunity against mumps in vaccinated young adults, France 2013. Euro Surveill. 2016;21(10):30156.https://doi.org/10.2807/1560-7917.es.2016.21.10.30156.

7. Whyte D, O'Dea F, McDonnell C, O'Connell NH, Callinan S, Brosnan E, et al. Mumps epidemiology in the mid-west of Ireland 2004-2008: increasing disease burden in the university/college setting. Euro Surveill. 2009;14(16)

8. Aasheim ET, Inns T, Trindall A, Emmett L, Brown KE, Williams CJ, et al. Outbreak of mumps in a school setting, United Kingdom, 2013. Hum Vaccin Immunother. 2014;10(8):2446-9.https://doi.org/10.4161/hv.29484.

9. May M, Rieder CA, Rowe RJ. Emergent lineages of mumps virus suggest the need for a polyvalent vaccine. International Journal of Infectious Diseases. 2018;66:1-4.https://doi.org/https://doi.org/10.1016/j.ijid.2017.09.024.

10. Fu C, Xu J, Cai Y, He Q, Zhang C, Chen J, et al. Effectiveness of one dose of mumps vaccine against clinically diagnosed mumps in Guangzhou, China, 2006-2012. Hum Vaccin Immunother. 2013;9(12):2524-8.https://doi.org/10.4161/hv.26113.

11. Su QR, Liu J, Ma C, Fan CX, Wen N, Luo HM, et al. Epidemic profile of mumps in China during 2004-2013. Zhonghua yu fang yi xue za zhi [Chinese journal of preventive medicine]. 2016;50(7):611-4.https://doi.org/10.3760/cma.j.issn.0253-9624.2016.07.009.

12. Cui A, Zhu Z, Hu Y, Deng X, Sun Z, Zhang Y, et al. Mumps Epidemiology and Mumps Virus Genotypes Circulating in Mainland China during 2013-2015. PLoS One. 2017;12(1):e0169561.https://doi.org/10.1371/journal.pone.0169561.

13. WHO. Mumps virus vaccines: WHO position paper. Wkly Epidemiol Rec. 2017;82:51-60

14. Yu G, Li Y, Cai J, Yu D, Tang J, Zhai W, et al. Short-term effects of meteorological factors and air pollution on childhood hand-foot-mouth disease in Guilin, China. Sci Total Environ. 2019;646:460-70.https://doi.org/10.1016/j.scitotenv.2018.07.329.

15. Yang Y, Geng X, Liu X, Wang W, Zhang J. Association between the incidence of varicella and meteorological conditions in Jinan, Eastern China, 2012-2014. BMC Infect Dis. 2016;16:179.https://doi.org/10.1186/s12879-016-1507-1.

16. Pan M, Yang HP, Jian J, Kuang Y, Xu JN, Li TS, et al. Association of meteorological factors with seasonal activity of influenza A subtypes and B lineages in subtropical western China. Epidemiol Infect. 2019;147:e72.https://doi.org/10.1017/s0950268818003485.

17. Hao Y, Wang RR, Han L, Wang H, Zhang X, Tang QL, et al. Time series analysis of mumps and meteorological factors in Beijing, China. BMC Infect Dis. 2019;19(1):435.https://doi.org/10.1186/s12879-019-4011-6.

18. Li R, Lin H, Liang Y, Zhang T, Luo C, Jiang Z, et al. The short-term association between meteorological factors and mumps in Jining, China. Sci Total Environ. 2016;568:1069-75.https://doi.org/10.1016/j.scitotenv.2016.06.158.

19. Hubalek Z. North Atlantic weather oscillation and human infectious diseases in the Czech Republic, 1951-2003. European Journal of Epidemiology. 2005;20(3):263-70.https://doi.org/10.1007/s10654-004-6518-3.

20. Yu G, Yang R, Yu D, Cai J, Tang J, Zhai W, et al. Impact of meteorological factors on mumps and potential effect modifiers: An analysis of 10 cities in Guangxi, Southern China. Environmental research. 2018;166:577-87.https://doi.org/10.1016/j.envres.2018.06.047.

21. Zhou Z, Mei S, Chen J, Li X, Yin L, Liang J, et al. Nonlinear effect of wind velocity on mumps in Shenzhen, China, 2013–2016. Public health. 2020;179:178-85.https://doi.org/https://doi.org/10.1016/j.puhe.2019.10.023.

22. Yang Q, Yang Z, Ding H, Zhang X, Dong Z, Hu W, et al. The relationship between meteorological factors and mumps incidence in Guangzhou, China, 2005-2012. Hum Vaccin Immunother. 2014;10(8):2421-32.https://doi.org/10.4161/hv.29286.

23. Hu W, Li Y, Han W, Xue L, Zhang W, Ma W, et al. Meteorological factors and the incidence of mumps in Fujian Province, China, 2005-2013: Non-linear effects. Sci Total Environ. 2018;619-620:1286-98.https://doi.org/10.1016/j.scitotenv.2017.11.108.

24. Onozuka D, Hashizume M. Effect of weather variability on the incidence of mumps in children: a time-series analysis. Epidemiol Infect. 2011;139(11):1692-700.https://doi.org/10.1017/s0950268810002967.

25. Gasparrini A. Distributed Lag Linear and Non-Linear Models in R: The Package dlnm. J Stat Softw. 2011;43(8):1-20

26. Sane J, Gouma S, Koopmans M, de Melker H, Swaan C, van Binnendijk R, et al. Epidemic of mumps among vaccinated persons, The Netherlands, 2009-2012. Emerg Infect Dis. 2014;20(4):643-8.https://doi.org/10.3201/eid2004.131681.

27. Xu Q, Li R, Liu Y, Luo C, Xu A, Xue F, et al. Forecasting the Incidence of Mumps in Zibo City Based on a SARIMA Model. Int J Environ Res Public Health. 2017;14(8).https://doi.org/10.3390/ijerph14080925.

28. Zhu H, Zhao H, Ou R, Xiang H, Hu L, Jing D, et al. Epidemiological Characteristics and Spatiotemporal Analysis of Mumps from 2004 to 2018 in Chongqing, China. Int J Environ Res Public Health. 2019;16(17).https://doi.org/10.3390/ijerph16173052.

29. Kaffenberger BH, Shetlar D, Norton SA, Rosenbach M. The effect of climate change on skin disease in North America. Journal of the American Academy of Dermatology. 2017;76(1):140-7.https://doi.org/10.1016/j.jaad.2016.08.014.

30. Cheng Q, Bai L, Zhang Y, Zhang H, Wang S, Xie M, et al. Ambient temperature, humidity and

hand, foot, and mouth disease: A systematic review and meta-analysis. Sci Total Environ. 2018;625:828-36.https://doi.org/10.1016/j.scitotenv.2018.01.006.

31. Peci A, Winter AL, Li Y, Gnaneshan S, Liu J, Mubareka S, et al. Effects of Absolute Humidity, Relative Humidity, Temperature, and Wind Speed on Influenza Activity in Toronto, Ontario, Canada. Applied and environmental microbiology. 2019;85(6).https://doi.org/10.1128/aem.02426-18.

32. Mahmud AS, Metcalf CJ, Grenfell BT. Comparative dynamics, seasonality in transmission, and predictability of childhood infections in Mexico. (1469-4409 (Electronic))

33. Ho YC, Su BH, Su HJ, Chang HL, Lin CY, Chen H, et al. The association between the incidence of mumps and meteorological parameters in Taiwan. Hum Vaccin Immunother. 2015;11(6):1406-12.https://doi.org/10.1080/21645515.2015.1029687.

34. Zhang D, Guo Y, Rutherford S, Qi C, Wang X, Wang P, et al. The relationship between meteorological factors and mumps based on Boosted regression tree model. Sci Total Environ. 2019;695:133758.https://doi.org/10.1016/j.scitotenv.2019.133758.

35. Phung D, Nguyen HX, Nguyen HLT, Luong AM, Do CM, Tran QD, et al. The effects of socioecological factors on variation of communicable diseases: A multiple-disease study at the national scale of Vietnam. PloS one. 2018;13(3):e0193246-e.https://doi.org/10.1371/journal.pone.0193246.

36. Belanger M, Gray-Donald K, O'Loughlin J, Paradis G, Hanley J. Influence of weather conditions and season on physical activity in adolescents. Ann Epidemiol. 2009;19(3):180-6.https://doi.org/10.1016/j.annepidem.2008.12.008.

37. Tang JW. The effect of environmental parameters on the survival of airborne infectious agents. J R Soc Interface. 2009;6 Suppl 6:S737-46.https://doi.org/10.1098/rsif.2009.0227.focus.

38. Guo Q, Dong Z, Zeng W, Ma W, Zhao D, Sun X, et al. The effects of meteorological factors on influenza among children in Guangzhou, China. Influenza and other respiratory viruses. 2019;13(2):166-75.https://doi.org/10.1111/irv.12617.

39. Zhong X, Qi, J., Li, H., Dong, L., & Gao, D. Seasonal distribution of microbial activity in bioaerosols in the outdoor environment of the Qingdao coastal region. Atmospheric Environment. 2016;140(06):506-13.https://doi.org/doi.org/10.1016/j.atmosenv.2016.06.034.

40. Jones AM, Harrison RM. The effects of meteorological factors on atmospheric bioaerosol concentrations--a review. Sci Total Environ. 2004;326(1-3):151-80.https://doi.org/10.1016/j.scitotenv.2003.11.021.

41. Sabariego S, Diaz de la Guardia C, Alba F. The effect of meteorological factors on the daily variation of airborne fungal spores in Granada (southern Spain). Int J Biometeorol. 2000;44(1):1-5

42. Yu L, Winter S, Xie D. The child play behavior and activity questionnaire: a parent-report measure of childhood gender-related behavior in China. Archives of sexual behavior. 2010;39(3):807-15.https://doi.org/10.1007/s10508-008-9403-4.

43. Hao J, Yang Z, Huang S, Yang W, Zhu Z, Tian L, et al. The association between short-term exposure to ambient air pollution and the incidence of mumps in Wuhan, China: A time-series study. Environmental research. 2019;177:108660.https://doi.org/10.1016/j.envres.2019.108660.

555

Response to Reviewers

Dear Editor,

We acknowledge with thanks the receipt of the comments of the reviewer. These comments/requirements were highly beneficial in the modification of the manuscript. The manuscript was revised according to all these comments. All modifications and/or corrections are highlighted by using the track changes mode. We would like to thank Editage (www.editage.cn) for English language editing. A point-to-point response to the reviewers' comments was attached. We appreciate you kindly offer the opportunity to transfer our manuscript.

Additional Editor Comments

Question No1: Abstract should be written according to Plos One models;

Response to the question No.1: We re-wrote the Abstract according to Plos One models.

Question No.2： It is necessary to include at the beginning of the Abstract and at the end of the Introduction what are the hypotheses of this research. This can be done in the form of a question;

Response to the question No.2: We added the hypotheses of this research in the article ,L81-86“However, the nonlinear and lag effects of climatic factors on the transmission among children, who are the main population affected by mumps, are still unclear at present. A study conducted in Japan explored the linear relationship between meteorological factors and mumps incidence among children via negative binomial regression analysis (24). What are the roles of meteorological factors on mumps incidence among children? There is an urgent need to explore these nonlinear relationships using lag effects..”

Question No.3： Keywords should be better chosen. They should not contain words that are in the title of the manuscript;

Response to the question No.3: Thanks for your question. As your suggestion, the Keywords were revised as following:“distributed lag nonlinear models, climatic factors, effect”.

Question No.4： There are several manuscripts addressing the theme "meteorological factors on mumps". In a quick search (https://www.sciencedirect.com/search/advanced?qs=meteorological%20factors%20 mumps) I found at least seven similar manuscripts. Therefore, the authors should make clear in the Introduction what is the difference in the work done for papers that are already published. This needs to be improved in the Introduction;

Response to the question No.4: Thanks for your question. As your suggestion, we described the difference of the similar researches on mumps, and the section were revised as **L68-86**.

Question No.5： In the Material and Methods is necessary a map of the study area location;

Response to the question No.5: The map of the study area location was added in the Material and Methods.

Question No.6： Although the authors employed robust statistical analyzes, further testing is still needed to improve the discussion of results. As the authors worked with an 18-year time series, it is necessary to check for changes in this time series with the nonparametric Man-Kendall and Pettitt tests. These tests are easily performed in the software R and may show if there was a tendency to increase or decrease in any meteorological variable and in the cases;

Response to the question No.6: We use the nonparametric Man-Kendall and Pettitt tests to check for changes in our time series data. And the result was shown as Table 1

Question No.7： Table 1 is important, but I would like to see a boxplot for these variables. Use the ggplot2 package;

Response to the question No.7： Thanks for your suggestion. We used the boxplot **(Fig 3.)** to describe these meteorological variables instead of the original table.

Question No.8： It is necessary to inform which test applied the Sperman correlations. In addition, I invite the authors to graphically express the results in Table 2 through the correlation network. It is a didactic way of representing this matrix. Can be done with the "qgraph" package in R;

Response to the question No.8: Thanks for your suggestion. We applied the correlation network**(Fig 5.)**to graphically express the results in the original Table 2.

Question No.9： It is necessary to inform in the legend of Figures 2, 3 and 4 if the adjusted models were significant and which test was used for this.

Response to the question No.9: Based on the AICs, we selected the smallest AIC to different meteorological variables to structure the best models. We showed the RR and 95% confidence interval (95%CI) of the original figure 2，3 and 4. Meantime, we could see the RR and 95% CI in the original figure 3 and 4 (The red lines represent mean relative risks and the grey regions represent 95%CIs.) If the 95% CI include "1" as the RR value, there is not significant difference. Otherwise, it means that it's significantly different. It equals to the P value <0.05.

Question No.10： Discussion is very poor and needs to be improved. Authors should include findings from recent manuscripts I mentioned;

Response to the question No.10: Thanks for your advice. We got some recent manuscripts the reviewer mentioned and other new article into reference added to 43 articles, and revised the discussion.

Question No.11： In the conclusions, it is necessary to inform readers about the scientific advances that this manuscript has obtained in relation to the works already published. What are the proposals to minimize the occurrence of mumps in children by knowing the behavior of meteorological variables?

Response to the question No.11:

In the conclusions, scientific advance was informed as **L358-361** "In general, compared to previous studies that focused on the effect of meteorological factors on mumps incidence, for the first time, we identified a significant nonlinear association between certain climatic variables and mumps incidence among children in Guangzhou with lag effects." And **L363-365**"Moreover, extreme effects of the variables mentioned above among mumps cases were detected in a subgroup analysis according to gender and age." Due to the results of our study, some proposals were recommended to minimize the occurrence of mumps among children. **L367-371** "For example, preventative measures and health education should be strengthened in the temperature range of 10–23.6ºC, and proper ventilation might be helpful in protecting children against infection with mumps. We also implied that children should cut down on going to crowded places such as playgrounds in the active period of mumps from May to July in Guangzhou."

Question No.12： The authors used only 35 references and most of them are old. In addition to being very little, recent references need to be included to demonstrate to readers that while there are many published manuscripts, the topic still needs to be studied.

Response to the question No.12: Thanks for your kindly reminder, We reviewed the recent references and revised the introduction, and demonstrated the scientific advance and the difference between our study and previous researches. Firstly our study use the DLNMs to explore the nonlinear relationships with lag effects between meteorological variables and mumps cases. Secondly we firstly used the DLNMs to analyze the meteorological effects on mumps cases among children. Finally, we analyze the effect by subpopulation by sex and age among children. This detail could be seen in the "introduction and discussion".

"

Reviewer #1:

The subject of the manuscript is interesting and topical, especially for the return of diseases previously eradicated or controlled in the last century. However, I found several weaknesses in the manuscript, for example, presentation of the results needs to improve, the discussion of the results does not cover other continents, only being restricted to Asia and Europe. As well as several gross errors throughout the manuscript, implying that the manuscript was not revised correctly. It is necessary to improve the quality of the figures to make them self explanatory. It is necessary to improve the conclusions of the manuscript.

Response to the question of reviewer 1： Thanks for your kindly suggestion. We review the recent article and add some researches from Canada and Mexico into the discussion. We reviewed English by Editage company to avoid gross errors in our article. We also revised and improved the conclusions of the manuscript, the scientific advance was mentioned in the conclusion(**L358-361, L363-365),** and the control measures to reduce the occurrence of mumps among children were recommended too(**L367-371)**.

Reviewer #2:

The manuscript makes an interesting based on interaction between mumps and meteorological variables. It is believed that more information on the subject can still be extracted, even in face of the limitations described by authors. On this aspect, some points need to be improved throughout the text, I believe they should include the references of R software, as well as dlnm package, both described on manuscript. The introduction and materials and methods are well described. Regarding the results, I have some suggestions for improvement:

Question No.1： Replacement of Figure 1 with a boxplot of mumps cases on an annual and monthly basis.

Response to the Question No.1： Thanks for your suggestion, we replaced the figure 1 with a boxplot of mumps cases on annual and monthly basis**(Fig 2.)**.

Question No.2：

The other variables would be displayed in Figure 2, with monthly boxplots, making the visualization of the results much more understandable in some passages of the text, exploring the maximum and minimum.

Response to the Question No.2： Thanks for your suggestion, we replaced the figure 2 with monthly boxplots, exploring the maximum and minimum**(Fig 3.)**.

Question No.3：

The old Figure 2 (which will be for Figure 3) could include the intensities, not only leaving the color red, this would show the interaction between them.

Response to the Question No.3: Thanks for your suggestion, we tried to change the old figure 3 with the intensities, however, we thought the original one would be better.

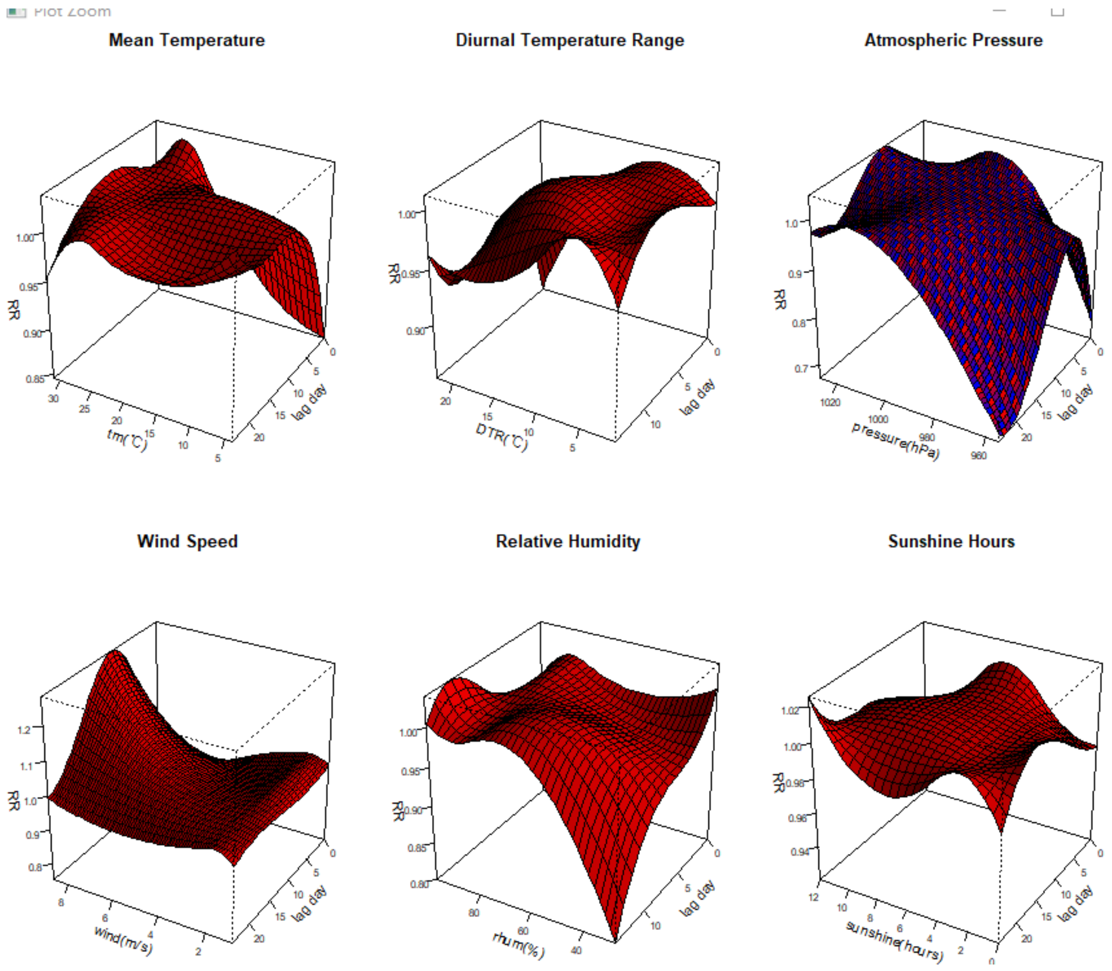

Question No.4:

Improve the image resolution of all images, as they are of low quality.

Response to the Question No.4: thanks for your suggestion, we fix the image resolution of all images due to the guideline in Plos One Journal.

Question No.5:

Paragraph preceding Figure 3, correct the pHa for hPa, on page 11. Well, from these improvements and implementations, the article will be able to be approved.

Response to the Question No.5: Thanks for your advice, We correct the pHa for hPa, on original page 11.
