# Supplementary material for: The role of meteorological factors on mumps incidence among children in Guangzhou, Southern China
Source: PLoS One. 2020 Apr 29;15(4):e0232273. doi: 10.1371/journal.pone.0232273 (PMC7190132; doi:10.1371/journal.pone.0232273)
Supplement: Attachment — Submitted filename: Response to the reviewers.docx [file pone.0232273.s003.docx]

**Response to Reviewers**

Dear Editor,

We acknowledge with thanks the receipt of the comments of the reviewer. These comments/requirements were highly beneficial in the modification of the manuscript. The manuscript was revised according to all these comments. All modifications and/or corrections are highlighted by using the track changes mode. We would like to thank Editage (www.editage.cn) for English language editing. A point-to-point response to the reviewers’ comments was attached. We appreciate you kindly offer the opportunity to transfer our manuscript.

**Additional Editor Comments**

**Question No1:**  Abstract should be written according to Plos One models;

**Response to the question No.1:** We re-wrote the Abstract according to Plos One models.

**Question No.2：**It is necessary to include at the beginning of the Abstract and at the end of the Introduction what are the hypotheses of this research. This can be done in the form of a question;

**Response to the question No.2:** We added the hypotheses of this research in the article ,L81-86“However, the nonlinear and lag effects of climatic factors on the transmission among children, who are the main population affected by mumps, are still unclear at present. A study conducted in Japan explored the linear relationship between meteorological factors and mumps incidence among children via negative binomial regression analysis (24). What are the roles of meteorological factors on mumps incidence among children? There is an urgent need to explore these nonlinear relationships using lag effects..”

**Question No.3：** Keywords should be better chosen. They should not contain words that are in the title of the manuscript;

**Response to the question No.3:** Thanks for your question. As your suggestion, the Keywords were revised as following:“distributed lag nonlinear models, climatic factors, effect”.

**Question No.4：**There are several manuscripts addressing the theme "meteorological factors on mumps". In a quick search (https://www.sciencedirect.com/search/advanced?qs=meteorological%20factors%20mumps) I found at least seven similar manuscripts. Therefore, the authors should make clear in the Introduction what is the difference in the work done for papers that are already published. This needs to be improved in the Introduction;

**Response to the question No.4:** Thanks for your question. As your suggestion, we described the difference of the similar researches on mumps, and the section were revised as **L68-86**.

**Question No.5：** In the Material and Methods is necessary a map of the study area location;

**Response to the question No.5**: The map of the study area location was added in the Material and Methods.

**Question No.6：** Although the authors employed robust statistical analyzes, further testing is still needed to improve the discussion of results. As the authors worked with an 18-year time series, it is necessary to check for changes in this time series with the nonparametric Man-Kendall and Pettitt tests. These tests are easily performed in the software R and may show if there was a tendency to increase or decrease in any meteorological variable and in the cases;

**Response to the question No.6:** We use the nonparametric Man-Kendall and Pettitt tests to check for changes in our time series data. And the result was shown as Table 1

**Question No.7：**Table 1 is important, but I would like to see a boxplot for these variables. Use the ggplot2 package;

**Response to the question No.7：**Thanks for your suggestion. We used the boxplot **(Fig 3.)** to describe these meteorological variables instead of the original table.

**Question No.8：**It is necessary to inform which test applied the Sperman correlations. In addition, I invite the authors to graphically express the results in Table 2 through the correlation network. It is a didactic way of representing this matrix. Can be done with the "qgraph" package in R;

**Response to the question No.8:** Thanks for your suggestion. We applied the correlation network**(Fig 5.)**to graphically express the results in the original Table 2.

**Question No.9：**It is necessary to inform in the legend of Figures 2, 3 and 4 if the adjusted models were significant and which test was used for this.

**Response to the question No.9:** Based on the AICs, we selected the smallest AIC to different meteorological variables to structure the best models. We showed the RR and 95% confidence interval (95%CI) of the original figure 2，3 and 4. Meantime, we could see the RR and 95% CI in the original figure 3 and 4 (The red lines represent mean relative risks and the grey regions represent 95%CIs.) If the 95% CI include “1” as the RR value, there is not significant difference. Otherwise, it means that it’s significantly different. It equals to the P value <0.05.

**Question No.10：**Discussion is very poor and needs to be improved. Authors should include findings from recent manuscripts I mentioned;

**Response to the question No.10:** Thanks for your advice. We got some recent manuscripts the reviewer mentioned and other new article into reference added to 43 articles, and revised the discussion.

**Question No.11：**In the conclusions, it is necessary to inform readers about the scientific advances that this manuscript has obtained in relation to the works already published. What are the proposals to minimize the occurrence of mumps in children by knowing the behavior of meteorological variables?

**Response to the question No.11:**

In the conclusions, scientific advance was informed as **L358-361** “In general, compared to previous studies that focused on the effect of meteorological factors on mumps incidence, for the first time, we identified a significant nonlinear association between certain climatic variables and mumps incidence among children in Guangzhou with lag effects.” And **L363-365**“Moreover, extreme effects of the variables mentioned above among mumps cases were detected in a subgroup analysis according to gender and age.” Due to the results of our study, some proposals were recommended to minimize the occurrence of mumps among children. **L367-371** “For example, preventative measures and health education should be strengthened in the temperature range of 10–23.6ºC, and proper ventilation might be helpful in protecting children against infection with mumps. We also implied that children should cut down on going to crowded places such as playgrounds in the active period of mumps from May to July in Guangzhou.”

**Question No.12：**The authors used only 35 references and most of them are old. In addition to being very little, recent references need to be included to demonstrate to readers that while there are many published manuscripts, the topic still needs to be studied.

**Response to the question No.12:** Thanks for your kindly reminder, We reviewed the recent references and revised the introduction, and demonstrated the scientific advance and the difference between our study and previous researches. Firstly our study use the DLNMs to explore the nonlinear relationships with lag effects between meteorological variables and mumps cases. Secondly we firstly used the DLNMs to analyze the meteorological effects on mumps cases among children. Finally, we analyze the effect by subpopulation by sex and age among children. This detail could be seen in the “introduction and discussion”.

**“**

**Reviewer #1:**

The subject of the manuscript is interesting and topical, especially for the return of diseases previously eradicated or controlled in the last century. However, I found several weaknesses in the manuscript, for example, presentation of the results needs to improve, the discussion of the results does not cover other continents, only being restricted to Asia and Europe. As well as several gross errors throughout the manuscript, implying that the manuscript was not revised correctly. It is necessary to improve the quality of the figures to make them self explanatory. It is necessary to improve the conclusions of the manuscript.

**Response to the question of reviewer 1：**Thanks for your kindly suggestion. We review the recent article and add some researches from Canada and Mexico into the discussion. We reviewed English by Editage company to avoid gross errors in our article. We also revised and improved the conclusions of the manuscript, the scientific advance was mentioned in the conclusion(**L358-361,** **L363-365**)**,** and the control measures to reduce the occurrence of mumps among children were recommended too( **L367-371)**.

**Reviewer #2:**

The manuscript makes an interesting based on interaction between mumps and meteorological variables. It is believed that more information on the subject can still be extracted, even in face of the limitations described by authors. On this aspect, some points need to be improved throughout the text, I believe they should include the references of R software, as well as dlnm package, both described on manuscript. The introduction and materials and methods are well described. Regarding the results, I have some suggestions for improvement:

**Question No.1：**Replacement of Figure 1 with a boxplot of mumps cases on an annual and monthly basis.

**Response to the Question No.1：**Thanks for your suggestion, we replaced the figure 1 with a boxplot of mumps cases on annual and monthly basis**(Fig 2.)**.

**Question No.2：**

The other variables would be displayed in Figure 2, with monthly boxplots, making the visualization of the results much more understandable in some passages of the text, exploring the maximum and minimum.

**Response to the Question No.2：**Thanks for your suggestion, we replaced the figure 2 with monthly boxplots, exploring the maximum and minimum**(Fig 3.)**.

**Question No.3：**

The old Figure 2 (which will be for Figure 3) could include the intensities, not only leaving the color red, this would show the interaction between them.

**Response to the Question No.3：**Thanks for your suggestion, we tried to change the old figure 3 with the intensities, however, we thought the original one would be better.

**Question No.4：**

Improve the image resolution of all images, as they are of low quality.

**Response to the Question No.4：**thanks for your suggestion, we fix the image resolution of all images due to the guideline in Plos One Journal.

**Question No.5：**

Paragraph preceding Figure 3, correct the pHa for hPa, on page 11. Well, from these improvements and implementations, the article will be able to be approved.

**Response to the Question No.5：**Thanks for your advice, We correct the pHa for hPa, on original page 11.
