# Supplementary material for: The role of meteorological factors on mumps incidence among children in Guangzhou, Southern China
Source: PLoS One. 2020 Apr 29;15(4):e0232273. doi: 10.1371/journal.pone.0232273 (PMC7190132; doi:10.1371/journal.pone.0232273)
Supplement: Attachment — Submitted filename: Response to the reviewers.docx [file pone.0232273.s005.docx]

**Response to Reviewers**

Dear Editor,

We acknowledge with thanks the receipt of the comments of the reviewer. These comments/requirements were highly beneficial in the modification of the manuscript. The manuscript was revised according to all these comments. All modifications and/or corrections are highlighted by using the track changes mode. We would like to thank Editage (www.editage.cn) for English language editing. A point-to-point response to the reviewers’ comments was attached. We appreciate you kindly offer the opportunity to transfer our manuscript.

**Reviewer #1:**

Thanks for your comments. We checked the article again and corrected the mistakes. For example, in Figure 8, we corrected the word "effet" for "effect", found in the central part.

**Reviewer #2:**

**Question No.1：**Does the impact of the variables used influence similarly for each age group or sex? It is believed to be interesting information to be explored.

**Response to the Question No.1：**Thanks for your suggestion, we analyzed the cumulative extreme effects of meteorological variables on mumps cases of children by sex and age at table 3. “In the analyses by subpopulation, the extreme low effects of temperature, atmospheric pressure, and relative humidity, and the extreme high effect of wind speed showed statistical significance in the female population, but no significant extreme effect was observed in the male population. The extreme low effect of temperature was observed between 4-6 years group (RR=0.25, 95%CI: 0.11-0.60) and 15-17 years group (RR=0.01, 95%CI: 0.00-0.57). The extreme high effect of atmospheric pressure was revealed at the 4-6 years group (RR=0.46, 95%CI: 0.21-0.98)”

**Question No.2：**

What is the main variable for the development of mumps? The results are presented throughout the text, with the data identified, so that you do not mention the most important variable in this context.

**Response to the Question No.2：**

Thanks for your suggestion. We structured different model to different variables. Each variable had its own characteristic to the development of mumps. So it was hard to say which variable was more important. The strongest interaction was observed between temperature and mumps cases, and tendency was easy for the public to get. Therefore, if we much choose one of the variables as the most important factors, we will choose the temperature.

**Question No.3：**

On line 142, create the article of the dlnm package.

**Response to the Question No.3：**

Thanks for your suggestion, we added the reference about DLNM package into the article.

**Question No.4：**

Table 1 deserves to be better explored, because it was reproduced in the text. And based on the trend signal, which is significant, what is the interaction between meteorological variables and the mumps?

**Response to the Question No.4：**

Thanks for your advice. We add the content to explore the table 1, “The variables of DTR, rain, pressure, wind, relative humidity were statistically significant. Wind and relative humidity had a rising tendency, whereas the DTR, rain and pressure had a decrease tendency. No tendency was observed among the variables of cases, temperature and sunshine hours.”

**Fig 5** shows the interaction network between meteorological factors and mumps cases among children, the strongest interaction was observed between temperature and mumps cases. Temperature, sunshine hour, relative correlation and aggregate rainfall had a positive interaction with mumps cases, whereas atmosphere pressure, wind speed and DTR had a negative interaction with mumps cases.

**Question No.5：**

On line 187, an ideal word would not be correlation, but interaction.

**Response to the Question No.5：**

We change the interaction instead of the correlation on line 197, thanks for your advice.

**Question No.6：**

On lines 204 and 205, how did you get confused? Would the RR be 60%?

**Response to the Question No.6：**

Maybe the expression made confusion. I revised the article as “However, the RRs just had a significantly increasing trend before the atmosphere pressure as 992 hPa and the relative humidity as 60%, respectively.”

**Question No.7：**

In Figure 8, correct the word "effet" for "effect", found in the central part

**Response to the Question No.7：**

We are sorry for this mistake. We revised the figure 8 and correct the word “effect”.

**Question No.8：**

In Table 2, include a valve on high-effect atmospheric pressure, from 088 to 0.88.

**Response to the Question No.8：**

We are sorry for this mistake. We corrected the 088 to 0.88.

Thanks again.
